# Unlocking TRPM7 interactions: A database-driven quest

**DOI:** 10.1016/j.csbj.2025.11.030

**Published:** 2025-11-16

**Authors:** Nicolas Jonckheere, Lise Rodat-Despoix, Isabelle Dhennin-Duthille, Alban Girault, Frédéric Hague, Mathieu Gautier

**Affiliations:** a Univ. Lille, CNRS, Inserm, CHU Lille, UMR9020-U1277—CANTHER—Cancer Heterogeneity Plasticity and Resistance to Therapies, Lille 59000, France; b Université de Picardie Jules Verne, UR-UPJV 4667, Amiens 80000, France

**Keywords:** TRPM7, Protein interactome, BioGRID database, GSEA

## Abstract

Transient receptor potential cation channel subfamily M member 7 (TRPM7) is a dual function protein comprising a non-selective cation channel and an atypical kinase domain. TRPM7 has been involved in many diseases including malignancies. Indeed, TRPM7 is proposed as a promising target for therapeutical drug design. Numerous studies have shown that TRPM7 interacts with proteins involved in regulating intracellular signaling. Therefore, a better understanding of the TRPM7 interactome would provide insight into pathophysiological mechanisms at the cellular and molecular levels. It could also open up new therapeutic avenues for molecules targeting either the proteins of interest directly or protein-protein interactions. In the first part of this work, we present the interaction partners described in the literature for TRPM7 and their potential impacts on cell biology. In the second part of the manuscript, we use public databases and protein interaction modeling tools to characterize the TRPM7 interactome. In particular, the analysis of the TRPM7 interactome using experimental data (BioGRID) and modeling tools (ProteinPrompt) has allowed us to isolate 19 genes of interest mainly related to small GTPase pathways involved in digestive neoplasia such as colorectal and pancreatic cancers. In summary, we provide an extended overview of potential TRPM7 interactors which need to be validated in cellular models. This will provide crucial insights into the molecular mechanisms at the tumor cell membrane, helping us to propose new therapeutic targets for precision medicine.

## Graphical Abstract

**Figure:**
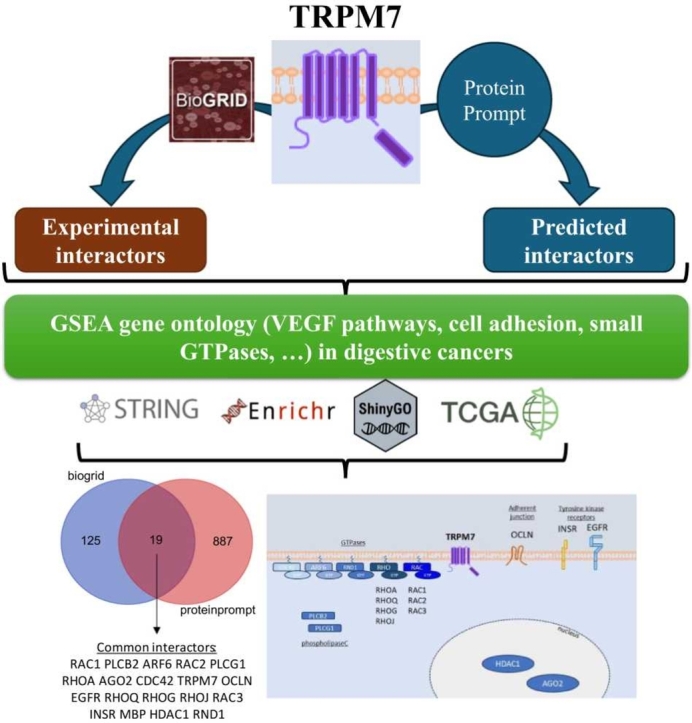

## 1 Introduction

Transient receptor potential cation channel subfamily M member 7 (TRPM7), also named CHAK1, LTRPC7, TRP-PLIK, is a dual function protein comprising a non-selective cation channel and an atypical kinase domain [1], [2], [3]. The TRPM7 subunit, a 212 kDa protein containing 1865 amino acids, can assemble as homotetramers to form a functional ion channel. But TRPM7 can also associate with TRPM6 subunits to form heterotetramers [4]. TRPM7 is ubiquitously expressed in human tissues and is required for cell viability and embryogenesis [5], [6], [7]. TRPM7 physiological roles are mainly due to its channel function. Thus, TRPM7 is essential for Ca^2^^+^ , Mg^2+^ and Zn^2+^ intestinal absorption, renal reabsorption [8], and more generally for cellular Mg^2+^ homeostasis regulation [9]. Electrophysiological experiments conducted on HEK293 and CHOK1 cells expressing TRPM7 have shown that the channel is also permeant for other metal divalent cations including Ni^2+^, Mn^2+^, Co^2+^, Cd^2+^ [10], [11], suggesting that TRPM7 could be involved in contamination by toxic metal traces. TRPM7 is involved in some pathologies related to disruption of Mg^2+^ homeostasis, including hypomagnesemia, macrothrombocytopenia, and trigeminal neuralgia [12]. Moreover, TRPM7 is overexpressed in many cancers where it regulates cell proliferation, migration and invasion through its channel function, but which also involves its kinase domain [13].

TRPM7 is a chanzyme due to its kinase domain located in the intracellular C-terminus. TRPM7 kinase domain is a serine/threonine kinase belonging to the α-kinase family [3], [14], [15]. In addition to TRPM6 and TRPM7, the α-kinase family includes the *Dictyostelium* Myosin Heavy Chain (MHC) kinases A, B, and C, and elongation factor-2 kinase (eEF2k) [14], [16]. In contrast to conventional protein kinases, α-kinases may phosphorylate on serine/threonine residues in α-helical conformations [16]. The crystal structure of the TRPM7 kinase domain has been determined by Yamaguchi *et al.* in 2001 [17]. Each kinase domain monomer is composed of 6 α-helices and 15 β-strands. The central catalytic core of the TRPM7 kinase domain has structural similarities to the classical protein kinase family including cAMP dependent protein kinase (PKA) [17]. In particular, the kinase domain displayed a conserved lysine residue at 1648 position which is absolutely required for catalytic activity, a C-terminal glycine-rich motif which may be involved in peptide substate recognition, and a Zn^2+^ binding module located in the C-terminal lobe which regulates kinase structure stability [17]. Regarding the mechanisms of TRPM7 channel gating, high-resolution structures of closed TRPM7 channels were resolved by cryo-electron microscopy (cryo-EM) [18]. By using a combination of site-directed mutagenesis, electrophysiological recordings, and modelling and molecular dynamics (MD) simulations, Schmidt *et al.* identified a pivotal Mg^2+^ regulatory site located within the lower channel gate at the position N1097, stabilizing the TRPM7 channel in the closed state [19]. They also identify the gain-of-function mutation N1098Q that promotes constitutively opened TRPM7 channel [19], [20]. Similarly, the resolutions of TRPM7 structures in closed and opened states have been made to determine the mechanisms of channel activation and inhibition by pharmacological opener (naltriben) or blockers (NS8593 and VER155008), respectively [20]. This study showed that naltriben stabilizes TRPM7 channel in the open conducting state by tying and pooling the N-terminal domains of the neighboring subunits leading to iris-like transformation of the cytoplasmic domain. Moreover, a vanilloid-type site involved in the stabilization of the closed state by NS8593 and VER155008 has been identified in the TRPM7 structure. Finaly, this vanilloid-type site is also involved in the TRPM7 inhibition by the anticancer agent CCT128930 suggesting a common mechanism for TRPM7 inhibition by pharmacological agents [21]. Interestingly, the TRPM7 kinase domain is regulated by divalent cations. Indeed, using protein phosphorylation assays on Myelin Basic Protein (MBP) and histone H3 as substrates of the purified TRPM7 kinase, Ryazanova *et al.*, showed that Mg^2+^ and Mn^2+^ are required as enzymatic cofactors [3]. Mn^2+^ stimulates TRPM7 kinase activity twice as much as Mg^2+^, while Zn^2+^ and Co^2+^ have an inhibitory effect. In the other hand, Ca^2+^ has no effect on the TRPM7 kinase activity [3]. Under physiological conditions, only intracellular Mg^2+^ divalent cations can regulate the TRPM7 kinase activity [3]. Contrarily to the channel function, the role of the kinase domain is largely unknown. The use of genetically modified mice showed that deletion of the kinase domain induced embryonic lethality identical to full TRPM7 KO mutant [9]. Interestingly, the heterozygous chimeric mice have an altered Mg^2+^ regulation and a lower survival rate when placed on Mg^2+^-deficient diets [9]. On the other hand, the K1648R mutation resulting on non-functional kinase did not induce any lethality nor channel function impairment, and improve survival in mice receiving Mg^2+^-deficient diets [22], [23]. Taken together, these data suggest that although the presence of the TRPM7 kinase domain is required for survival and Mg^2+^ regulation, its phosphotransferase function is not essential. It is possible that TRPM7 exerts biological functions by protein-protein interactions. In this review, the first part will recapitulate the known interactors of TRPM7 and the biological functions of these complexes. The aim of the second part is to propose new TRPM7 interactors by using online databases and computing tools.

## 2 Known interaction partners of TRPM7

### 2.1 AKT

AKT is a serine/threonine kinase activated downstream of phosphoinositide-3-kinase (PI3-K). PI3-K/AKT pathway is frequently dysregulated in numerous malignancies. By using an *in vitro* TRPM7 kinase assay, Hoeger *et al.* showed that AKT1 is a direct substrate of recombinant purified TRPM7 kinase [24]. In chronic myeloid leukemia (CML) cells, they showed that TRPM7 kinase regulates AKT and mothers against decapentaplegic homolog 2 (SMAD2) pathways leading to modulated cyclooxygenase-2 (COX-2) expression [24]. Thus, as a direct AKT-activating kinase, these findings reinforced the importance of TRPM7 in the regulation of oncogenic signaling pathways.

### 2.2 Annexin-1

Annexin-1 operates through interaction with membrane phospholipids in a Ca^2+^-dependent manner leading to numerous signaling pathways involved in inflammation, cell proliferation, differentiation and apoptosis. For example, Annexin-1 can bind with S100 EF-hand Ca^2+^-binding proteins to form complexes promoting membrane crosslink [25]. In 2004, Dorovkov and Ryazanov showed that Annexin-1 may be phosphorylated by the recombinant TRPM7 kinase at a conserved serine residue (Ser5) located within the N-terminal amphipathic α-helix [26]. Furthermore, they highlighted that N-terminal Annexin-1 peptide phosphorylation by TRPM7 impedes the capacity of Annexin-1 peptide to adopt a α-helicoidal conformation, to interact with membranes, and to bind with S100A11 protein [27]. Interestingly, Annexin expression has been found to be altered in many cancers, mainly resulting in a loss of expression in tumor tissue [25]. However, the interaction between Annexin-1 and TRPM7 has not yet been demonstrated in cancer cells, nor in preclinical models.

### 2.3 Ca^2+^-binding proteins: calmodulin and S100A1

It has been also shown that EF-hand Ca^2+^ binding proteins may bind directly to TRPM7 through its N-terminal region. In particular, Bousova *et al.* identified binding sites for calmodulin and S100A1 within the TRPM7 N-terminal region [28]. In this study, a peptide corresponding to the TRPM7 N-terminal region has been synthetized and the interactions with calmodulin and S100A1 were investigated by *in vitro* fluorescence anisotropy and by *in silico* molecular modeling [28]. Both calmodulin and S100A1 are able to form complexes with TRPM7 N-terminal intracellular region at position T523-L535 with dissociation constants in the micromolar level ranges [28]. Nevertheless, this mechanism has not yet been clearly demonstrated at the cellular or whole animal level. Mishra *et al.* showed that TRPM7-like currents are inhibited by increasing cytosolic Ca^2+^ in rat hepatocytes and hepatoma cells [29]. In particular, the study demonstrated that Ca^2+^-inhibition of TRPM7-like currents is partially mediated by the Ca^2+^/calmodulin-dependent protein kinase II (CaMKII) activity. Interestingly, a CaMKII target sequence has been identified within the COOH-terminal domain of rat TRPM7. On the other hand, no consensus sequence for a calmodulin binding site has been revealed, suggesting that calmodulin and Ca^2+^ act indirectly through CaMKII rather than *via* a direct interaction with the channel. TRPM7 has been involved in neuronal death in a mouse model of hypoxic-ischemic brain cell death [30]. Waixenicin A has been used as a potential inhibitor of TRPM7 to prevent brain injury. By proteomic analysis and western-blots, it has been showed that the waixenicin treatment prevented the reduced CaMKII expression induced by brain injury. This study suggests that, CaMKII is activated by Ca^2+^ influx through TRPM7 channels. Taken together, the findings of these studies did not reveal any evidence of direct interaction between Ca^2+^-dependent proteins and TRPM7 channels.

### 2.4 CNNMs, PRL-1 and ARL15

TRPM7 is the major regulator of Mg^2+^ homeostasis in mammals [8], [9]. However, there are several Mg^2+^ channels and transporters which have been identified but their functions are not yet fully understood [31]. Mg^2+^ is equally distributed on both intra- and extracellular compartments, resulting in a lower electrochemical force for membrane diffusion of this cation compared to other cations such as Na^+^ or Ca^2+^. Interestingly, two groups parallelly described a new mechanism of Mg^2+^ inflow through TRPM7 involving interaction with CNNM (Cyclin And CBS Domain Divalent Metal Cation Transport Mediator) proteins [32], [33]. CNNM are transmembrane proteins containing a N-terminal extracellular domain, a transmembrane domain, and two intracellular domains which are a CBS-pair domain (or Bateman domain) and a Cyclic Nucleotide-Binding Homology (CNBH) domain [34], [35]. CNNM have been identified as Mg^2+^ transporters but their role in Mg^2+^ inflow or lowering by extrusion is still under debate [31], [36]. Mass spectroscopy analysis on purified endogenous TRPM7 from rodent brain or on overexpressed TRPM7 in HEK-293T cells identified CNNMs, Phosphatase of Regenerating Liver 1 (PRL-1), and ADP-ribosylation factor-like GTPase 15 (ARL15) as potential interactors [32], [33]. Interaction between TRPM7 and CNNMs was confirmed by co-immunoprecipitation assay (for CNNM1, 2, 3 and 4) and by proximity-dependent biotin identification (BioID) (for CNNM3). Moreover, KO of *CNNM3* or *CNNM4* significantly reduced TRPM7-dependent Mg^2+^ entry, and decreased TRPM7 currents recorded by whole-cell patch-clamp. On the other hand, the Mg^2+^-lowering activities of CNNM occur independently of TRPM7 [32]. Functional co-expression in heterologous expression system, using WT or kinase dead TRPM7, showed that ARL15 strongly affects TRPM7 channel function, while CNNM3 appears to acts as a negative regulator of TRPM7 kinase activity [33].

It has been shown that TRPM7 and CNNM form a trimeric complex with ARL15 [32], [33], [37]. ARL15 binds specifically to CNNM CBS-pair domain and inhibits CNMM and TRPM7 Mg^2+^ transport [34], [37]. Tetteh *et al.* went further inside TRPM7 regulation by CNNMs by showing that CNNM2 CBS-pair domain modulates TRPM7 channel activity through the action of ARL15, whereas CNNM2 CNBH domain binds to TRPM7 kinase domain and enhanced its catalytic activity *in vitro* [38]. Importantly, ARL15 and PRL compete for CNNM binding, regulating CNNM-TRPM7 interaction and Mg^2+^ homeostasis [34]. PRL2 overexpression counteracts ARL15 binding to CNNM3 and enhances TRPM7 function in a magnesium-dependent mechanism, suggesting that PRL2 acts as a magnesium biosensor controlling the CNNM3/TRPM7 interaction [39]. Jolly *et al.* recently proposed two models based on direct interaction between TRPM7 and CNNMs with ARL15/PRL as modulators of CNNM, or indirect interaction through ARL15 [36].

Interestingly, PRL and CNNM have been documented in colorectal and breast cancers. Funato *et al.* showed that PRL3 binds to CNNM4 leading to the inhibition of Mg^2+^ efflux. High Mg^2+^ intracellular levels promote cell energy metabolism and cell proliferation through AMPK/mTOR pathway. Moreover, they showed an overexpression of PRL3 and a downregulation of CNNM4 in colorectal cancers suggesting that CNNM4 prevents tumor progression by controlling low Mg^2+^ intracellular levels and by regulating energy metabolism [40]. Hardy *et al.* showed that PRL2 is overexpressed in human breast cancer tissues and in metastatic lymph nodes, and that PRL2 knockdown decreases cell migration of MDA-MB-231 and mouse mammary tumor-derived cell lines, as well as tumor progression in mice models [41]. They further showed that PRL2 binds to CNNM3 to promote Mg^2+^ entry leading to cell proliferation and tumor growth. Importantly, CNNM3 expression was positively correlated with PRL2 and Ki-67 expressions in human breast cancer tissues [42]. Moreover, CNNMs expression was analyzed in digestive cancers using Genotype Tissue Expression (GTEx) and The Cancer Genome Atlas (TCGA) datasets [43]. CNNM3 is overexpressed in esophageal carcinoma whereas CNNM2 is overexpressed in esophageal and stomach cancers. On the other hand, an overexpression of CNNM4 was observed in esophageal, stomach, pancreatic, colon and rectal cancers. The high expression of CNNM4 is also correlated with high expression of TRPM7 and MAGT1, another magnesium transporter, and this TRPM7/MAGT1/CNNM4 signature is associated with low patient survival in pancreatic adenocarcinoma [43].

The PRL/ARL/CNNM/TRPM complex, recently named the PACT complex [36], has been well described in heterologous overexpression systems and only few studies demonstrated the involvement of PRL and CNNM in cancer. Given that TRPM7 is overexpressed in numerous types of cancers, and involved in cell proliferation, migration and invasion, it is tempting to speculate that the PACT complex may play a significant role in cancer cell fates by regulating their intracellular Mg^2+^ levels.

### 2.5 CREB

Song *et al.* showed that the cAMP Response Element Binding protein (CREB) is an ideal substate of TRPM7 for studying kinase activity by LANCE *Ultra* assay [44]. The ability of purified TRPM7 kinase domain to phosphorylate the CREB peptide led to the identification of TG100–115 as a new inhibitor of TRPM7 kinase activity and breast cancer cell migration [44]. The interaction between CREB and TRPM7 has been confirmed in dental cells. By using a model of TRPM7 kinase-inactive knock-in mutant mice that have normal TRPM7 ion channel functions (TRPM7 KR mice), Ogata *et al.* showed a lower expression of phosphorylated CREB in incisors of TRPM7 KR mice compared to control mice [45]. Moreover, co-immunoprecipitation experiments revealed that TRPM7 and CREB may interact in ameloblast-lineage cells [45]. In cancer and endothelial cells, TRPM7 is essential for cellular glycolysis regulation through CREB-dependent pathway [46]. However, in this study of Wu *et al.*, TRPM7 regulated CREB phosphorylation in an indirect manner, *via* Ca^2+^ influx-induced calcineurin activation [46].

### 2.6 eEF2-k

Eukaryotic elongation factor 2 (eEF2) plays a central role in protein translation by binding to ribosomes. eEF2 is phosphorylated by its kinase eEF2-k, leading to its failure to bind ribosomes. Perraud *et al.* showed that eEF2 phosphorylation is increased under low Mg^2+^ conditions [47]. By GST pulldown assays, they showed that TRPM7 interacts with eEF2-k but not eEF2 to adapt cell metabolism to Mg^2+^ availability [47].

### 2.7 EGFR

Co-immunoprecipitation experiments, proximity ligation assays (PLA) and confocal microscopy showed an interaction between TRPM7 and epidermal growth factor (EGF) receptor (EGFR) in primary vascular smooth muscle cells (VSMC) isolated from rat mesenteric arteries [48]. From a mechanistic point of view, EGF stimulated TRPM7-EGFR interactions in a c-Src-dependent manner, and EGF/EGFR phosphorylated TRPM7 leading to enhanced VSMC proliferation and migration [48]. Interestingly, TRPM7 acted both upstream and downstream of EGF/EGFR pathway because EGFR expression was downregulated in TRPM7 deficient mice leading to vascular remodeling with aortic wall thinning [48].

### 2.8 Histone H3

Histone H3 was one of the first TRPM7 substrate to be identified by using the purified C-terminal part of the protein containing kinase catalytic domain expressed in bacteria [3]. In SV40 MES 13 mouse mesangial cell line, Clapham’s group showed that TRPM7 kinase domain may be cleaved from the transmembrane channel by caspase to form TRPM7 cleaved kinase fragments (M7CKs) [49], [50]. These M7CKs translocated in the nucleus where they bound to nuclear proteins including transcription factors (*see section k*). Interaction between M7CKs and nuclear proteins led to histone H3 at Ser10 and Ser28 phosphorylation which is essential for transcription regulation, DNA repair, and mitotic chromatin condensation [50]. In this model, TRPM7 channel function regulated Zn^2+^ influx which is required for interaction between M7CKs and nuclear proteins containing zinc-finger [50]. Indeed, both channel and kinase functions seem necessary to regulate TRPM7-dependent gene regulation [50].

### 2.9 Myelin basic protein

As for histone H3, Myelin Basic Protein (MBP) has been identified as TRPM7 substrate for its kinase activity [2], [3], [51]. Nevertheless, the physiological significance of interaction between TRPM7 and MBP has not been yet elucidated.

### 2.10 Myosin

Through three complementary studies, van Leeuwen's group has endeavored to decipher the mechanisms by which TRPM7 regulates the assembly and stability of myosin filaments, and consequently the contraction of actomyosin fibers in adhesion processes. Based on the fact that TRPM7 (as TRPM6) encode channel-kinases, they hypothesized that the coupling of a cation channel to an α-kinase in a single polypeptide could explain the close relationship between Ca^2+^ signaling and Ca^2+^-dependent actomyosin remodeling. They firstly established that activation of TRPM7 by bradykinin (BK), a Gq-PLC-coupled receptor agonist that induces Ca^2+^-dependent phosphorylation of the Myosin Heavy Chain (MHC) and promotes actomyosin relaxation in mouse N1E-115 neuroblastoma cells [52], leads to a Ca^2+^- and kinase-dependent interaction with the actomyosin cytoskeleton. Accordingly, overexpression of TRPM7 increases intracellular Ca^2+^ levels accompanied by cell spreading, adhesion and the formation of focal adhesions. Activation of TRPM7 induces the transformation of these focal adhesions into podosomes by a kinase-dependent mechanism, whereas cells expressing kinase-dead TRPM7 failed to induce podosomes in response to BK stimulation. Moreover, they demonstrate that TRPM7 interacts with myosin IIA through its COOH-terminus and requires an active kinase domain [53]. They next demonstrated that TRPM7 directly phosphorylates the mouse and human heavy chain of non-muscle myosin IIA, specifically targeting a short stretch of amino acids within its α-helical tail. This phosphorylation regulates myosin IIA filament stability and cortical localization, thereby influencing cytoskeletal dynamics and cellular contractility [54]. This property could also be found in TRPM6 subtype. While TRPM6 and TRPM7 share electrophysiological properties and cellular functions, these channels are non-redundant genes raising the possibility that the kinases have distinct substrates. In that context, they demonstrate that TRPM6 and TRPM7 phosphorylate the assembly domain of myosin IIA, IIB and IIC on identical residues. Whereas phosphorylation of myosin IIA is restricted to the coiled-coil domain, TRPM6 and TRPM7 also phosphorylate the non-helical tails of myosin IIB and IIC [55].

### 2.11 Nuclear proteins

The detection of M7CKs which were specifically localized in the nucleus prompted the investigation of their potential interaction with nuclear proteins [50]. Krapivinsky *et al.* identified RYBP, Ruvbl1/pontin, Ruvbl2/reptin, DDX3X, DDB1, and DBC1 as TRPM7 potential interactors in a yeast-two hybrid (Y2H) screen of a rat brain library and by using tandem affinity purification with C-terminal TRPM7 fragment [50], [56]. These results were confirmed by co-immunoprecipitation experiments in HEK293T cell co-expressing system, and by GST-pulldown assays [50]. Although TRPM7 kinase interacts with these nuclear proteins, it does not directly phosphorylate them [50].

### 2.12 PAK1

Our recent data showed that TRPM7 interacts with the serine/threonine-protein kinase PAK1 in PANC-1 pancreatic cancer cell line by using co-immunoprecipitation and PLA experiments [57]. PAK1 is a protein kinase involved in intracellular signaling by regulating small GTPase activation that plays an important role in cell adhesion, migration and invasion. The treatment with TG100–115 abolished the formation of PLA complexes which strongly suggests that TRPM7 kinase activity regulates the interaction between PAK1 and TRPM7 in PANC-1 cells [57]. Moreover, the endogenous deletion of TRPM7 kinase domain led to PANC-1 cell migration impairment, epithelial-like phenotype maintaining, and abolition of tumor growth in a model of mouse subcutaneous xenograft [57].

### 2.13 PLC

In 2002, Clapham’s laboratory identified a 146-amino-acid carboxy-terminal segment of the TRPM7 kinase (PLIK; phospholipase C interacting kinase) domain in a Y2H screen of a rat brain library. By GST-pulldown assays, they demonstrated that the PLIK domain of TRPM7 directly interacts with the C2 domain of phospholipase C (PLC-β1,2,3 and γ1) from HEK-293T cells transiently expressing constructs (TRPM7 and PLC-β1–4, PLC-γ1 or PLC-δ1) [58]. Through this interaction, TRPM7 can modulate PLC signaling pathways, thereby affecting PIP₂ metabolism and subsequent Ca²⁺ mobilization. TRPM7 channels are highly permeable to Ca^2+^, and Runnels and collaborators demonstrated that the TRPM7 COOH-terminus associates with phospholipase C (PLC) isoforms and that PLC activation regulates TRPM7 channel opening [2], [58], [59]. For instance, in platelets, alterations in TRPM7 kinase activity have been linked to impaired PIP₂ metabolism, leading to reduced Ca²⁺ responses upon stimulation of major platelet receptors [60]*.* The results of these various studies lead to the conclusion that the PLC substrate phosphatidylinositol 4,5-bisphosphate (PIP_2_) is a key regulator of the TRPM7 signaling.

### 2.14 RhoA

Additionally, TRPM7 has been implicated in the regulation of RhoA activity by Gudermann’s group. Their work established that, treatment during 24 h at 30 µM by NS8593 (a TRPM7 inhibitor) or TRPM7 knock-down of human hepatocyte carcinoma cell line HuH7, decreases RhoA activity by 50 % and drastically reduces both the TRPM7/RhoA interaction and proximity [61]. They set out to further dissect the underlying molecular mechanisms of this interaction by using recombinant TRPM7 kinase and Histone-tagged RhoA to confirm that TRPM7 kinase phosphorylated RhoA at Ser188 in a dose-dependent manner [61]. As a matter of fact, they demonstrated that through its functional coupling *via* its kinase domain, TRPM7 modulates the actomyosin cytoskeleton by affecting RhoA signaling pathways, which are crucial for various cellular processes, including migration and adhesion. Recently, our group confirmed that RhoA co-immunoprecipitates with TRPM7 in a kinase-dependent manner in human pancreatic ductal adenocarcinoma cell line PANC-1 [57].

### 2.15 SMAD2

Mothers against decapentaplegic homolog 2 (SMAD2) protein is an intracellular signal transducer and a transcriptional regulator activated by TGF-β leading to cell activation or differentiation. Romagnani *et al.* showed that TRPM7 regulates TGF-β/SMAD2 signaling pathway in mouse T cells [62]. By *in vitro* kinase assay, their work highlighted that TRPM7 kinase domain directly phosphorylates SMAD2 at the C-terminal Ser465/467 motif. Moreover, they identified close proximity between TRPM7 and SMAD2 in T cells by using PLA assays. These results showed that TRPM7 regulates T cell differentiation by direct phosphorylation of SMAD2 leading to its translocation into the nucleus [62].

### 2.16 STIM2

Faouzi *et al.* showed that the TRPM7 kinase domain is required for the regulation of Store-Operated Calcium Entry (SOCE) in DT40 B-lymphocytes [63]. They showed that TRPM7 channel function contributes to intracellular Ca^2+^ homeostasis by filling the intracellular Ca^2+^ stores. They further showed that SOCE was similarly inhibited in cells overexpressing TRPM7 mutants with kinase domain deletion (Δ-kinase) or inactivation (K1648R), indicating that the TRPM7 kinase domain regulates SOCE in these cells. STIM2 has been suggested as a possible partner of TRPM7 because TRPM7 inhibition by NS8595 did not inhibit SOCE in STIM2-KO cells unlike Orai1-KO and Orai2-KO. However, a direct interaction between STIM and TRPM7 proteins remains to be determined.

### 2.17 Synaptic vesicle proteins: synaptotagmin I, synapsin I, and snapin

Krapivinsky *et al.* identified the presence of TRPM7 in the membrane of synaptic vesicles of sympathetic neurons [56]. Moreover, they showed that snapin protein is a potential interactor of TRPM7 by Y2H screening of a rat brain library. Snapin is highly enriched in synaptic vesicles and its phosphorylation stimulates synaptic transmission. By co-immunoprecipitation experiments, they identified synaptotagmin I and synapsin I as additional interactors of TRPM7 in rat synaptic vesicles. In a physiological point of view, TRPM7 downregulation led to acetylcholine release impairment [56].

### 2.18 Tropomodulin

Tropomodulin 1 has been identified as a potential substrate of the TRPM7 kinase domain [64]. The phosphorylation sites are located in the N-terminal domain including serine and threonine residues in the tropomyosin-binding and actin-capping regions. Thus, it has been hypothesized that the tropomodulin phosphorylation by TRPM7 kinase may regulate the dynamics of actin filaments.

### 2.19 TRPM6/TRPM7

*TRPM6* is the *TRPM7* gene paralog [65]. TRPM6 can assemble with TRPM7 to form heterotetramers whose expression is restricted to the intestine and to the distal convoluted tubule (DCT) of the kidney [66] where they facilitate Mg^2+^ cellular influx, and notably epithelial Mg^2+^ absorption and reabsorption [4]. The TRPM6 and TRPM7 subunits are their own substrates due to the presence of serine/threonine-rich domains upstream of the kinase domain [17]. Clark *et al.*, highlighted that massive autophosphorylation of TRPM7 increases kinase activity and substrate recognition [67]. Li *et al.* assessed the electrophysiological properties of TRPM6, TRPM7 and TRPM6/TRPM7 channels in heterologous expression systems (CHOK1 and HEK293 cells) and mouse distal convoluted tube (MDCT) cells [11]. They firstly demonstrated that the relative permeability to Ni^2+^ is significantly different among these channels with the following sequence: TRPM7 >TRPM6/TRPM7 >TRPM6. Secondly, they showed that inward currents through TRPM6/TRPM7 channels were more sensitive to low extracellular pH than for TRPM6 or TRPM7 channels. Thirdly, the single channel conductance of TRPM6 channel (83.6 pS) was larger than those of TRPM6/TRPM7 (56.6 pS) and TRPM7 (40.1 pS) channels. Finally, 500 µM of 2-aminoethoxydiphenyl borate (2-APB) dramatically increased TRPM6 currents while it decreased TRPM7 and only slightly increased TRPM6/TRPM7 currents. Thus, these channels may play different physiological roles depending of their expression profile.

## 3 New insight from online protein interaction databases

### 3.1 Interactors with experimental evidences from BioGRID

We queried the BioGRID (Biological General Repository for Interaction Datasets) database for protein interactions with human TRPM7. BioGRID is an open-access database that records protein and genetic interactions from multiple species [68]. Protein interactions are determined by mass spectrometry (MS) techniques such as Affinity Capture-MS and Proximity Label-MS. Affinity Capture-MS is based on affinity captured on bait protein contained in cell extracts by polyclonal antibody or epitope tag. Proximity Label-MS method (such as BioID [69]) is based on bait-enzyme fusion protein that modify vicinal proteins. Affinity Capture-MS allows identification of partner in endogenous state (post-translational modification) in cells, and can also show multiprotein complex. BioID can also show transient interactions and is highly sensitive, but may require further biochemical validation. The affinity captured partners can also be validated by western-blots. The purification step is thought to get rid of potential contaminating proteins and provide high confidence interaction partners. Reconstituted Complex method, such as Glutathione-S-transferase (GST) pull-down, relies on recombinant proteins or cellular extracts with purified baits, and represents a more reliable biochemical method.

By querying the BioGRID database, we obtained the list of 144 interactors (Table 1). Low throughput strategies such as two hybrid methods led to identification of interaction between TRPM7 and phospholipase C proteins such as PLCB1, PLCB2 [58] or ITSN1 [70]. TRPM7, HIST3H3 and MBP were shown to be direct substrates of the TRPM7 kinase activity [3]. Moreover, 95 interactors were identified by high throughput Proximity Label MS. Similarly, 55 interactors have been identified by BioID. Several works performed unbiased interrogation of genetic interaction using CRISPR screening and led to identification of SCYL1, EGFR, KRAS, RIT1, FBXW7 and FASN as interactors [71], [72], [73], [74]. However, these genetic interactions are based on dependency regarding cell survival or proliferation and are not necessarily mediated by direct physical interaction.

**Table 1:** List of TRPM7 interactors enlisted in the BioGRID database.

Official Symbol Interactor A	Official Symbol Interactor B	Synonyms Interactor A	Experimental System	Experimental System Type	Source
PLCB2	TRPM7	PLC-beta−2	Two-hybrid	physical	[58]
PLCB1	TRPM7	EIEE12|PI-PLC|PLC−154|PLC-I|PLC154|PLCB1A|PLCB1B	Two-hybrid	physical	[58]
TRPM7	TRPM7	ALSPDC|CHAK|CHAK1|LTRPC7|LTrpC−7|TRP-PLIK	Biochemical Activity	physical	[3]
TRPM7	HIST3H3	ALSPDC|CHAK|CHAK1|LTRPC7|LTrpC−7|TRP-PLIK	Biochemical Activity	physical	[3]
TRPM7	MBP	ALSPDC|CHAK|CHAK1|LTRPC7|LTrpC−7|TRP-PLIK	Biochemical Activity	physical	[3]
TRPM7	PLCG1	ALSPDC|CHAK|CHAK1|LTRPC7|LTrpC−7|TRP-PLIK	Reconstituted Complex	physical	[58]
TRPM7	PLCB1	ALSPDC|CHAK|CHAK1|LTRPC7|LTrpC−7|TRP-PLIK	Reconstituted Complex	physical	[58]
TRPM7	PLCB2	ALSPDC|CHAK|CHAK1|LTRPC7|LTrpC−7|TRP-PLIK	Reconstituted Complex	physical	[58]
TRPM7	PLCB3	ALSPDC|CHAK|CHAK1|LTRPC7|LTrpC−7|TRP-PLIK	Reconstituted Complex	physical	[58]
TRPM7	PLCB2	ALSPDC|CHAK|CHAK1|LTRPC7|LTrpC−7|TRP-PLIK	Affinity Capture-Western	physical	[58]
SNAP29	TRPM7	CEDNIK|SNAP−29	Affinity Capture-MS	physical	[75]
ZACN	TRPM7	L2|LGICZ|LGICZ1|ZAC|ZAC1	Affinity Capture-MS	physical	[75]
TCTN3	TRPM7	C10orf61|JBTS18|OFD4|TECT3	Proximity Label-MS	physical	[76]
HDAC1	TRPM7	GON−10|HD1|RPD3|RPD3L1	Affinity Capture-MS	physical	[77]
RAB7A	TRPM7	PRO2706|RAB7	Affinity Capture-MS	physical	[77]
VAPA	TRPM7	VAP−33|VAP-A|VAP33|hVAP−33	Affinity Capture-MS	physical	[77]
Bub1	TRPM7	AL022991|Bub1a|C80208|D2Xrf87	Affinity Capture-MS	physical	[77]
C1qbp	TRPM7	AA407365|AA986492|D11Wsu182e|HABP1|P32|gC1qBP	Affinity Capture-MS	physical	[77]
Eef1a1	TRPM7	-	Affinity Capture-MS	physical	[77]
Flot1	TRPM7	reggie−2	Affinity Capture-MS	physical	[77]
Timeless	TRPM7	C77407|Debt69|tim	Affinity Capture-MS	physical	[77]
Tmed2	TRPM7	1110032D12Rik|1810020N21Rik|Rnp24|Sid394|p24beta1	Affinity Capture-MS	physical	[77]
Cep152	TRPM7	AI851464|mKIAA0912	Affinity Capture-MS	physical	[77]
ARL15	TRPM7	ARFRP2	Affinity Capture-MS	physical	[78]
TEX28	TRPM7	CXorf2|MRX99|TEX28P1|TEX28P2|fTEX	Affinity Capture-MS	physical	[78]
SNAP29	TRPM7	CEDNIK|SNAP−29	Affinity Capture-MS	physical	[78]
PNLDC1	TRPM7	-	Affinity Capture-MS	physical	[78]
ZACN	TRPM7	L2|LGICZ|LGICZ1|ZAC|ZAC1	Affinity Capture-MS	physical	[78]
TRIM25	TRPM7	EFP|RNF147|Z147|ZNF147	Affinity Capture-RNA	physical	[79]
K8.1	TRPM7	-	Affinity Capture-MS	physical	[80]
SCYL1	TRPM7	GKLP|NKTL|NTKL|P105|TAPK|TEIF|TRAP	Negative Genetic	genetic	[71]
TRPM7	TRPM7	ALSPDC|CHAK|CHAK1|LTRPC7|LTrpC−7|TRP-PLIK	Negative Genetic	genetic	[71]
HRAS	TRPM7	C-BAS/HAS|C-H-RAS|C-HA-RAS1|CTLO|H-RASIDX|HAMSV|HRAS1|RASH1|p21ras	Proximity Label-MS	physical	[81]
KRAS	TRPM7	C-K-RAS|CFC2|K-RAS2A|K-RAS2B|K-RAS4A|K-RAS4B|KI-RAS|KRAS1|KRAS2|NS|NS3|RASK2	Proximity Label-MS	physical	[81]
NRAS	TRPM7	ALPS4|CMNS|N-ras|NCMS|NRAS1|NS6	Proximity Label-MS	physical	[81]
CANX	TRPM7	CNX|IP90|P90	Proximity Label-MS	physical	[82]
LAMP1	TRPM7	CD107a|LAMPA|LGP120	Proximity Label-MS	physical	[82]
CA9	TRPM7	CAIX|MN	Proximity Label-MS	physical	[83]
RHBDD1	TRPM7	RRP4	Proximity Label-MS	physical	[84]
FAM105A	TRPM7	NET20	Proximity Label-MS	physical	[85]
RPL27	TRPM7	L27	Proximity Label-MS	physical	[86]
E	TRPM7	env|envelope|SARS-CoV2 E|E protein|emp|SARS-CoV−2 E|VEMP_SARS2|PRO_0000449651	Proximity Label-MS	physical	[87]
M	TRPM7	mem|membrane|SARS-CoV2 M|M protein|SARS-CoV−2 M|VME1_SARS2|PRO_0000449652	Proximity Label-MS	physical	[87]
nsp4	TRPM7	ORF1ab|ORF1ab-nsp4|SARS-CoV2 nsp4|SARS-CoV−2 nsp4|R1AB_SARS2|PRO_0000449622	Proximity Label-MS	physical	[87]
nsp6	TRPM7	ORF1ab|ORF1ab-nsp6|SARS-CoV2 nsp6|SARS-CoV−2 nsp6|R1AB_SARS2|PRO_0000449624	Proximity Label-MS	physical	[87]
ORF14	TRPM7	SARS-CoV2 ORF14|SARS-CoV−2 ORF14|14|Y14_SARS2|PRO_0000449658	Proximity Label-MS	physical	[87]
ORF7a	TRPM7	SARS-CoV2 ORF7a|SARS-CoV−2 ORF7a|Protein 7a|7a|NS7A_SARS2|PRO_0000449654	Proximity Label-MS	physical	[87]
ORF7b	TRPM7	SARS-CoV2 ORF7b|SARS-CoV−2 ORF7b|7b|NS7B_SARS2|PRO_0000449799	Proximity Label-MS	physical	[87]
S	TRPM7	spike|SARS-CoV2 S|SARS-CoV2 spike|S protein|surface|SARS-CoV−2 spike|SARS-CoV−2 S|SPIKE_SARS2|PRO_0000449646	Proximity Label-MS	physical	[87]
ORF7a	TRPM7	SARS-CoV2 ORF7a|SARS-CoV−2 ORF7a|Protein 7a|7a|NS7A_SARS2|PRO_0000449654	Proximity Label-MS	physical	[88]
E	TRPM7	env|envelope|SARS-CoV2 E|E protein|emp|SARS-CoV−2 E|VEMP_SARS2|PRO_0000449651	Proximity Label-MS	physical	[88]
ORF8	TRPM7	SARS-CoV2 ORF8|SARS-CoV−2 ORF8|8|NS7B_SARS2|PRO_0000449655	Proximity Label-MS	physical	[88]
M	TRPM7	mem|membrane|SARS-CoV2 M|M protein|SARS-CoV−2 M|VME1_SARS2|PRO_0000449652	Proximity Label-MS	physical	[88]
S	TRPM7	spike|SARS-CoV2 S|SARS-CoV2 spike|S protein|surface|SARS-CoV−2 spike|SARS-CoV−2 S|SPIKE_SARS2|PRO_0000449646	Proximity Label-MS	physical	[88]
ORF6	TRPM7	SARS-CoV2 ORF6|SARS-CoV−2 ORF6|6|NS6_SARS2|PRO_0000449653	Proximity Label-MS	physical	[88]
nsp6	TRPM7	ORF1ab|ORF1ab-nsp6|SARS-CoV2 nsp6|SARS-CoV−2 nsp6|R1AB_SARS2|PRO_0000449624	Proximity Label-MS	physical	[88]
nsp4	TRPM7	ORF1ab|ORF1ab-nsp4|SARS-CoV2 nsp4|SARS-CoV−2 nsp4|R1AB_SARS2|PRO_0000449622	Proximity Label-MS	physical	[88]
ST7	TRPM7	ETS7q|FAM4A|FAM4A1|HELG|RAY1|SEN4|TSG7	Proximity Label-MS	physical	[89]
FKBP8	TRPM7	FKBP38|FKBPr38	Proximity Label-MS	physical	[90]
PTPN1	TRPM7	PTP1B	Proximity Label-MS	physical	[90]
RHOT2	TRPM7	ARHT2|C16orf39|MIRO−2|MIRO2|RASL	Proximity Label-MS	physical	[90]
SLC25A46	TRPM7	-	Proximity Label-MS	physical	[90]
FASN	TRPM7	FAS|OA−519|SDR27X1	Positive Genetic	genetic	[74]
RPN1	TRPM7	OST1|RBPH1	Proximity Label-MS	physical	[91]
DNAJC16	TRPM7	-	Proximity Label-MS	physical	[92]
DNAJC1	TRPM7	DNAJL1|ERdj1|HTJ1|MTJ1	Proximity Label-MS	physical	[92]
SEC63	TRPM7	DNAJC23|ERdj2|PRO2507|SEC63L	Proximity Label-MS	physical	[92]
AGO2	TRPM7	EIF2C2|Q10	Affinity Capture-RNA	physical	[93]
ARF6	TRPM7	-	Proximity Label-MS	physical	[94]
ATP2A1	TRPM7	ATP2A|SERCA1	Proximity Label-MS	physical	[94]
B3GAT1	TRPM7	CD57|GLCATP|GLCUATP|HNK1|LEU7|NK−1|NK1	Proximity Label-MS	physical	[94]
BCAP31	TRPM7	6C6-AG|BAP31|CDM|DDCH|DXS1357E	Proximity Label-MS	physical	[94]
CEP135	TRPM7	CEP4|KIAA0635|MCPH8	Proximity Label-MS	physical	[94]
CKAP4	TRPM7	CLIMP−63|ERGIC−63|p63	Proximity Label-MS	physical	[94]
CYP2C9	TRPM7	CPC9|CYP2C|CYP2C10|CYPIIC9|P450IIC9	Proximity Label-MS	physical	[94]
DHFRL1	TRPM7	DHFRP4	Proximity Label-MS	physical	[94]
DIRAS3	TRPM7	ARHI|NOEY2	Proximity Label-MS	physical	[94]
ELOVL5	TRPM7	HELO1|SCA38|dJ483K16.1	Proximity Label-MS	physical	[94]
EMD	TRPM7	EDMD|LEMD5|STA	Proximity Label-MS	physical	[94]
ERGIC1	TRPM7	ERGIC−32|ERGIC32|NET24	Proximity Label-MS	physical	[94]
ERGIC2	TRPM7	Erv41|PTX1|cd002	Proximity Label-MS	physical	[94]
GJA1	TRPM7	AVSD3|CMDR|CX43|GJAL|HLHS1|HSS|ODDD	Proximity Label-MS	physical	[94]
GJD3	TRPM7	CX31.9|Cx30.2|GJA11|GJC1	Proximity Label-MS	physical	[94]
HSD17B11	TRPM7	17-BETA-HSD11|17-BETA-HSDXI|17BHSD11|DHRS8|PAN1B|RETSDR2|SDR16C2	Proximity Label-MS	physical	[94]
HSD3B7	TRPM7	CBAS1|PFIC4|SDR11E3	Proximity Label-MS	physical	[94]
KRT18	TRPM7	CYK18|K18	Proximity Label-MS	physical	[94]
KRT19	TRPM7	CK19|K19|K1CS	Proximity Label-MS	physical	[94]
LAMP1	TRPM7	CD107a|LAMPA|LGP120	Proximity Label-MS	physical	[94]
LAMP2	TRPM7	CD107b|LAMP−2|LAMPB|LGP110	Proximity Label-MS	physical	[94]
LAMP3	TRPM7	CD208|DC LAMP|DC-LAMP|DCLAMP|LAMP|LAMP−3|TSC403	Proximity Label-MS	physical	[94]
LMAN1	TRPM7	ERGIC−53|ERGIC53|F5F8D|FMFD1|MCFD1|MR60|gp58	Proximity Label-MS	physical	[94]
KIAA1715	TRPM7	LNP|LNP1|Ul|ulnaless	Proximity Label-MS	physical	[94]
LRRC59	TRPM7	p34	Proximity Label-MS	physical	[94]
METTL7A	TRPM7	AAM-B	Proximity Label-MS	physical	[94]
NDC80	TRPM7	HEC|HEC1|HsHec1|KNTC2|TID3|hsNDC80	Proximity Label-MS	physical	[94]
NUP155	TRPM7	ATFB15|N155	Proximity Label-MS	physical	[94]
OCLN	TRPM7	BLCPMG|PPP1R115	Proximity Label-MS	physical	[94]
PANX1	TRPM7	MRS1|PX1|UNQ2529	Proximity Label-MS	physical	[94]
PXMP2	TRPM7	PMP22	Proximity Label-MS	physical	[94]
RAB2A	TRPM7	LHX|RAB2	Proximity Label-MS	physical	[94]
RAB35	TRPM7	H-ray|RAB1C|RAY	Proximity Label-MS	physical	[94]
RAB5C	TRPM7	L1880|RAB5CL|RAB5L|RABL	Proximity Label-MS	physical	[94]
RAB9A	TRPM7	RAB9	Proximity Label-MS	physical	[94]
RHOB	TRPM7	ARH6|ARHB|MST081|MSTP081|RHOH6	Proximity Label-MS	physical	[94]
RPN1	TRPM7	OST1|RBPH1	Proximity Label-MS	physical	[94]
RPN2	TRPM7	RIBIIR|RPN-II|RPNII|SWP1	Proximity Label-MS	physical	[94]
SEC61B	TRPM7	-	Proximity Label-MS	physical	[94]
SEC62	TRPM7	Dtrp1|HTP1|TLOC1|TP−1	Proximity Label-MS	physical	[94]
SSR1	TRPM7	TRAPA	Proximity Label-MS	physical	[94]
STX7	TRPM7	-	Proximity Label-MS	physical	[94]
SYNE3	TRPM7	C14orf49|NET53|Nesp3	Proximity Label-MS	physical	[94]
PNLDC1	TRPM7	-	Affinity Capture-MS	physical	[95]
ZACN	TRPM7	L2|LGICZ|LGICZ1|ZAC|ZAC1	Affinity Capture-MS	physical	[95]
RABEP2	TRPM7	FRA	Affinity Capture-MS	physical	[95]
RYK	TRPM7	D3S3195|JTK5|JTK5A|RYK1	Affinity Capture-MS	physical	[95]
TEX28	TRPM7	CXorf2|MRX99|TEX28P1|TEX28P2|fTEX	Affinity Capture-MS	physical	[95]
NKAIN1	TRPM7	FAM77C	Affinity Capture-MS	physical	[95]
SNAP29	TRPM7	CEDNIK|SNAP−29	Affinity Capture-MS	physical	[95]
CD3D	TRPM7	CD3-DELTA|IMD19|T3D	Affinity Capture-MS	physical	[95]
PSCA	TRPM7	PRO232	Affinity Capture-MS	physical	[95]
PTP4A1	TRPM7	HH72|PRL−1|PRL1|PTP(CAAX1)|PTPCAAX1	Affinity Capture-MS	physical	[95]
PKD2L2	TRPM7	TRPP5	Affinity Capture-MS	physical	[95]
TTYH1	TRPM7	-	Affinity Capture-MS	physical	[95]
EIF2B5	TRPM7	CACH|CLE|EIF−2B|EIF2Bepsilon|LVWM	Affinity Capture-MS	physical	[95]
ARL15	TRPM7	ARFRP2	Affinity Capture-MS	physical	[95]
C3orf52	TRPM7	TTMP	Affinity Capture-MS	physical	[95]
CCDC107	TRPM7	PSEC0222	Affinity Capture-MS	physical	[95]
ANPEP	TRPM7	APN|CD13|GP150|LAP1|P150|PEPN	Proximity Label-MS	physical	[96]
TMPRSS11B	TRPM7	-	Proximity Label-MS	physical	[96]
CLEC4D	TRPM7	CLEC−6|CLEC6|CLECSF8|MCL|MPCL	Proximity Label-MS	physical	[96]
CLEC4E	TRPM7	CLECSF9|MINCLE	Proximity Label-MS	physical	[96]
RHOG	TRPM7	ARHG	Affinity Capture-MS	physical	[97]
RHOA	TRPM7	ARH12|ARHA|RHO12|RHOH12	Affinity Capture-MS	physical	[97]
CDC42	TRPM7	CDC42Hs|G25K	Affinity Capture-MS	physical	[97]
RHOQ	TRPM7	ARHQ|HEL-S−42|RASL7A|TC10|TC10A	Affinity Capture-MS	physical	[97]
RHOB	TRPM7	ARH6|ARHB|MST081|MSTP081|RHOH6	Affinity Capture-MS	physical	[97]
RHOD	TRPM7	ARHD|RHOHP1|RHOM|Rho	Affinity Capture-MS	physical	[97]
RHOF	TRPM7	ARHF|RIF	Affinity Capture-MS	physical	[97]
RHOU	TRPM7	ARHU|CDC42L1|G28K|WRCH1|hG28K	Affinity Capture-MS	physical	[97]
RHOH	TRPM7	ARHH|TTF	Affinity Capture-MS	physical	[97]
RND1	TRPM7	ARHS|RHO6|RHOS	Affinity Capture-MS	physical	[97]
RND2	TRPM7	ARHN|RHO7|RhoN	Affinity Capture-MS	physical	[97]
RND3	TRPM7	ARHE|Rho8|RhoE|memB	Affinity Capture-MS	physical	[97]
RHOJ	TRPM7	ARHJ|RASL7B|TC10B|TCL	Affinity Capture-MS	physical	[97]
RAC1	TRPM7	Rac−1|TC−25|p21-Rac1	Affinity Capture-MS	physical	[97]
RAC2	TRPM7	EN−7|Gx|HSPC022|p21-Rac2	Affinity Capture-MS	physical	[97]
RAC3	TRPM7	-	Affinity Capture-MS	physical	[97]
RHOC	TRPM7	ARH9|ARHC|H9|RHOH9	Affinity Capture-MS	physical	[97]
RHOV	TRPM7	ARHV|CHP|WRCH2	Affinity Capture-MS	physical	[97]
EGFR	TRPM7	ERBB|ERBB1|HER1|NISBD2|PIG61|mENA	Negative Genetic	genetic	[72]
KRAS	TRPM7	C-K-RAS|CFC2|K-RAS2A|K-RAS2B|K-RAS4A|K-RAS4B|KI-RAS|KRAS1|KRAS2|NS|NS3|RASK2	Negative Genetic	genetic	[72]
RIT1	TRPM7	NS8|RIBB|RIT|ROC1	Negative Genetic	genetic	[72]
RAB7A	TRPM7	PRO2706|RAB7	Proximity Label-MS	physical	[98]
ARL15	TRPM7	ARFRP2	Proximity Label-MS	physical	[99]
INSR	TRPM7	CD220|HHF5	Proximity Label-MS	physical	[100]
SFN	TRPM7	YWHAS	Proximity Label-MS	physical	[101]
YWHAB	TRPM7	GW128|HEL-S−1|HS1|KCIP−1|YWHAA	Proximity Label-MS	physical	[101]
YWHAE	TRPM7	14–3−3E|HEL2|KCIP−1|MDCR|MDS	Proximity Label-MS	physical	[101]
YWHAG	TRPM7	14–3–3GAMMA|PPP1R170	Proximity Label-MS	physical	[101]
YWHAH	TRPM7	YWHA1	Proximity Label-MS	physical	[101]
YWHAQ	TRPM7	14–3–3|1C5|HS1	Proximity Label-MS	physical	[101]
YWHAZ	TRPM7	14–3–3-zeta|HEL-S−3|HEL4|KCIP−1|YWHAD	Proximity Label-MS	physical	[101]
Rab18	TRPM7	AA959686	Proximity Label-MS	physical	[102]
FBXW7	TRPM7	AGO|CDC4|FBW6|FBW7|FBX30|FBXO30|FBXW6|SEL−10|SEL10|hAgo|hCdc4	Negative Genetic	genetic	[73]
ESYT1	TRPM7	FAM62A|MBC2	Proximity Label-MS	physical	[103]
ITSN1	TRPM7	ITSN|SH3D1A|SH3P17	Two-hybrid	physical	[70]
MCAM	TRPM7	CD146|MUC18	Proximity Label-MS	physical	[104]

**Fig. 1:**
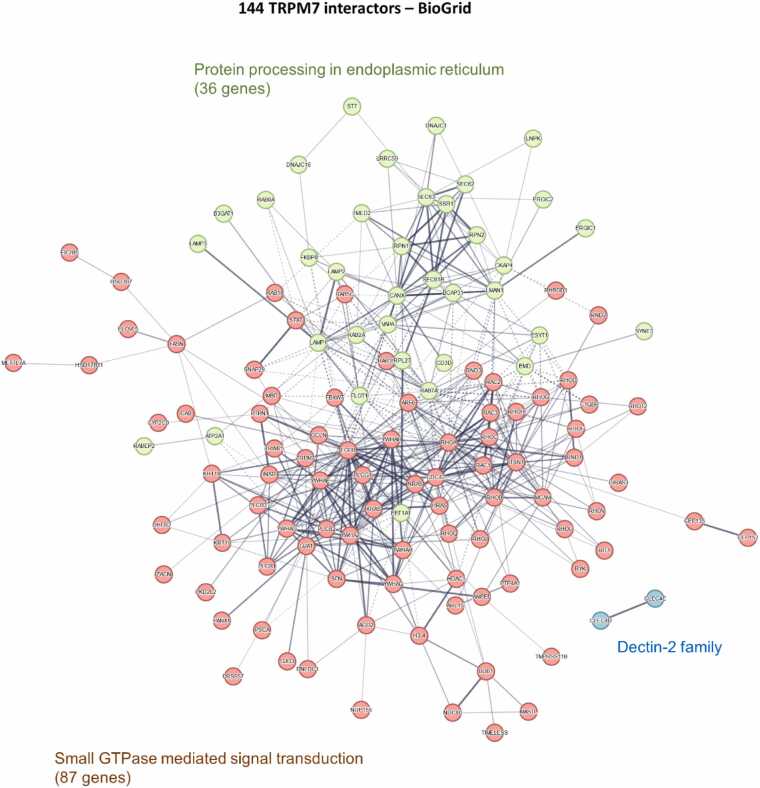
STRING protein-protein network of TRPM7 interactors from BioGRID database. 144 TRPM7 interactors were retrieved from BioGRID database. STRING network was realized using https://string-db.org/. Three k-means clusters were separated based on their centroids. Description of the overall ontology of clusters is indicated.

The interaction network was generated using STRING tool [105]. Two major subnetworks were identified and illustrated in the Fig. 1: 87 genes were mostly associated with Small GTPase mediated signal transduction and 36 genes were described as involved in protein processing in endoplasmic reticulum.

**Fig. 2:**
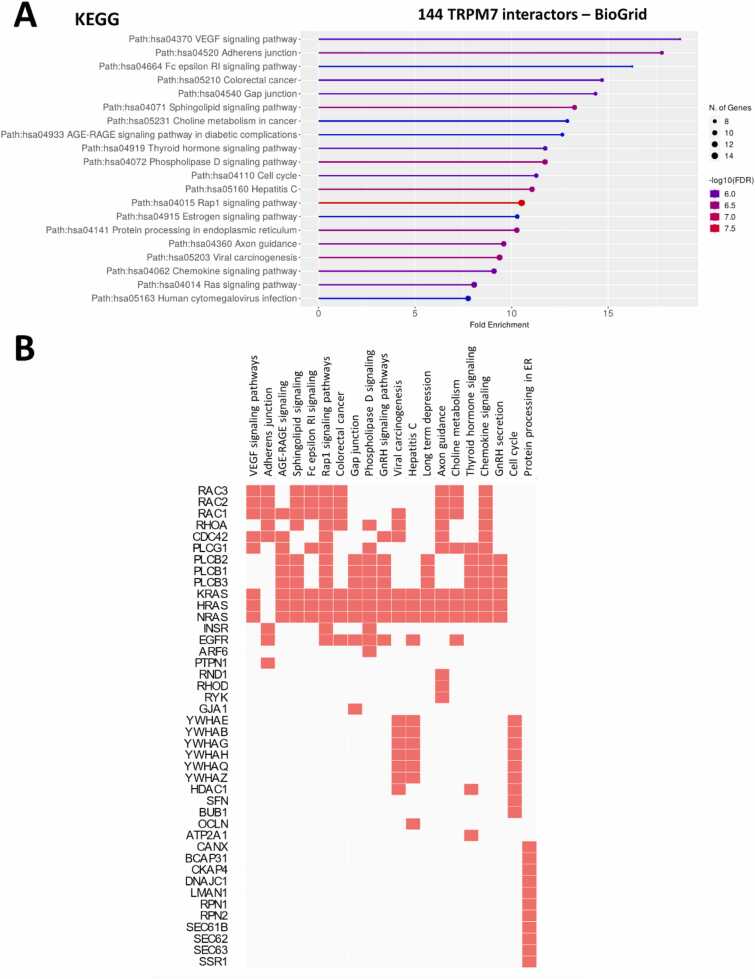
Ontology of TRPM7 interactors from BioGRID. (**A**) Dot plot of Gene-set enrichment analysis using ShinyGO v0.81 tool. Dot points are sized by the proportion of all proteins annotated with the KEGG corresponding term and coloured by enrichment confidence (FDR). (**B**) Clustergram of 144 TRPM7 interactors from BioGRID was realized using Enrichr tool (https://maayanlab.cloud/Enrichr/enrich). Enriched Terms are displayed as columns and input genes as rows. Genes associated with a term are indicated as red boxes in the matrix.

We performed a gene-set enrichment analysis (GSEA) using ShinyGO tool [106]. Kyoto Encyclopedia of Genes and Genomes (KEGG) terms corresponding to the VEGF signaling pathway (hsa04370), Adherens junction (hsa04520), gap junction (hsa04540), sphingolipid signaling pathway (hsa04071), and phospholipase D signaling pathway (hsa04072) were significantly enriched (Fig. 2). The biological processes associated with cell morphology (GO:0030865 cortical cytoskeleton organization, GO:0008360 reg. of cell shape, GO:0022604 reg. of cell morphogenesis), cell signaling (GO:0007264 small GTPase mediated signal transduction, GO:0007265 Ras protein signal transduction) or protein trafficking (GO:0006886 intracellular protein transport, GO:0034613 cellular protein localization) were significantly highlighted. VEGF signaling pathway and adherens/gap junction terms were significantly associated with GTPase such as RAC1/2/3, RHOA, CDC42 or HRAS/KRAS/NRAS (Fig. 2).

### 3.2 Predicted TRPM7 interactome

The protein interactome was predicted using ProteinPrompt that is based on machine learning using the sequences of protein pairs as input to estimate their tendency to bind [107]. We selected a threshold of 0.75 as it corresponds with the lowest false positive rate. Q96QT4 amino acid sequence which is the canonical sequence of TRPM7 was used as input. We identified 906 proteins that are predicted to interact with TRPM7 (Table S1). Interestingly, TRPM7 is predicted to form a homomeric protein (ProteinPrompt score = 1.0). Among the best prediction, TRPM6 is a genuine partner (ProteinPrompt score = 0.9360). Most of the known interactors described in this manuscript showed fair ProteinPrompt scores with the exception of PACT network formed by PRL1/PTP4A1, CNNMs and ARL15 proteins (Table 2). Additionally, among the list of TRPM7 interaction partners identified by Bai *et al.* that are not enlisted in BioGRID database, only IRAK1 (ProteinPrompt score = 0.77) belongs to the predicted list of TRPM7 interactors, suggesting a potential mechanistic link between TRPM7 and Toll-like receptor (TLR)/Interleukin-1 receptor (IL-1R) signaling pathway in cancer cells. Moreover, IRAK1 activation is frequently associated with progression and therapeutic resistance of cancers [108].

**Table 2:** List of TRPM7 known interactors and interaction prediction using ProteinPrompt.

Protein	Interation_score (interaction = 1)	Uniprot ID	Full description
AKT1	0.8787	P31749	RAC-alpha serine/threonine-protein kinase
AKT2	0.7280	P31751	RAC-beta serine/threonine-protein kinase
AKT3	0.6200	Q9Y243	RAC-gamma serine/threonine-protein kinase
Q5TZZ9	0.6720	Q5TZZ9	ANXA1 {ECO:0000313|EMBL:ADZ76495.1}
E7ETZ0	0.7293	E7ETZ0	CALM1 {ECO:0000313|Ensembl:ENSP00000403491}
S10A1	0.7960	P23297	Protein S100-A1
CNNM1	0.4360	Q9NRU3	Metal transporter CNNM1
CNNM2	0.4000	Q9H8M5	Metal transporter CNNM2
CNNM3	0.4133	Q8NE01	Metal transporter CNNM3
CNNM4	0.5080	Q6P4Q7	Metal transporter CNNM4
TP4A1	0.4440	Q93096	Protein tyrosine phosphatase type IVA 1 precursor
ARL15	0.5200	Q9NXU5	ADP-ribosylation factor-like protein 15
Q5U0J5	0.7720	Q5U0J5	CREB1 {ECO:0000313|EMBL:EAW70405.1}
EF2	0.9907	P13639	Elongation factor 2
EGFR	0.8173	P00533	Epidermal growth factor receptor precursor
H31T	0.9733	Q16695	Histone H3.1t
H31	0.9520	P68431	Histone H3.1
H32	0.9093	Q71DI3	Histone H3.2
H33	0.9013	P84243	Histone H3.3
B2R4P9	0.9013	B2R4P9	Histone H3
MBP	0.7893	P02686	Myelin basic protein
MYH1	0.6373	P12882	Myosin−1
MYH2	0.6907	Q9UKX2	Myosin−2
MYH3	0.5400	P11055	Myosin−3
MYO3A	0.5880	Q8NEV4	Myosin-IIIa
MYO3B	0.5440	Q8WXR4	Myosin-IIIb
MYH4	0.5787	Q9Y623	Myosin−4
Q7Z7A5	0.6387	Q7Z7A5	Myosin 5B
MYH6	0.5387	P13533	Myosin−6
MYH7	0.5680	P12883	Myosin−7
MYH7B	0.3987	A7E2Y1	Myosin−7B
MYH8	0.6120	P13535	Myosin−8
MYH9	0.7600	P35579	Myosin−9
MYH10	0.7560	P35580	Myosin−10
MYH11	0.6107	P35749	Myosin−11
MYH13	0.5720	Q9UKX3	Myosin−13
MYH14	0.5107	Q7Z406	Myosin−14
MYH15	0.4693	Q9Y2K3	Myosin−15
PAK1	0.7480	Q13153	Serine/threonine-protein kinase PAK 1
PLCB2	0.9333	Q00722	1-phosphatidylinositol 4,5-bisphosphate phosphodiesterase beta−2
PLCG1	0.9280	P19174	1-phosphatidylinositol 4,5-bisphosphate phosphodiesterase gamma−1
PLCG2	0.7427	P16885	1-phosphatidylinositol 4,5-bisphosphate phosphodiesterase gamma−2
PLCB3	0.7307	Q01970	1-phosphatidylinositol 4,5-bisphosphate phosphodiesterase beta−3
PLCB1	0.6933	Q9NQ66	1-phosphatidylinositol 4,5-bisphosphate phosphodiesterase beta−1
PLCH2	0.5960	O75038	1-phosphatidylinositol 4,5-bisphosphate phosphodiesterase eta−2
PLCB4	0.5760	Q15147	1-phosphatidylinositol 4,5-bisphosphate phosphodiesterase beta−4
RHOA	0.8747	P61586	Transforming protein RhoA precursor
SMAD2	0.8747	Q15796	Mothers against decapentaplegic homolog 2
SNAPN	0.7760	O95295	SNARE-associated protein Snapin
SYN1	0.7333	P17600	Synapsin−1
SYT1	0.6773	P21579	Synaptotagmin−1

Syndecan-2 (SDC2), activating signal cointegrator 1 (TRIP4), lamin-B1, AT-rich interactive domain-containing protein 2 (ARID2) did not reach the 0.75 cut off but still displayed fair interaction score (0.6147–0.7027), suggesting either a weak physical interaction or the need of cofactors to stabilize the protein complexes. Direct proof of physical interaction such as co-immunoprecipitation remains necessary to validate these TRPM7 interactors. Alternatively, instead of forming a tightly bound complex, TRPM7 and partners with mild/low interaction scores may be localized in the vicinity to regulate TRPM7 biological function.

**Fig. 3:**
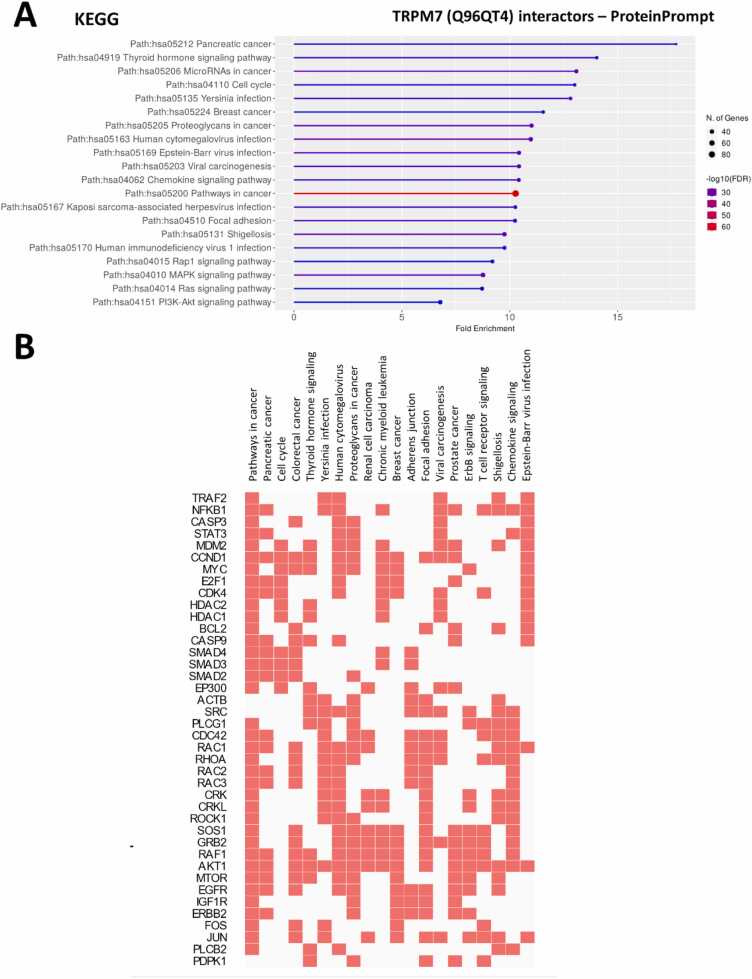
Ontology of the TRPM7 predicted interactome. (**A**) The protein interactome was determined using Q96Q4T amino acid sequence (canonical sequence of TRPM7) as input. Gene-set enrichment analyses were performed using ShinyGO v0.81 tool. (**B**) Clustergram of TRPM7 predicted interactors from ProteinPrompt was realized using Enrichr tool (https://maayanlab.cloud/Enrichr/enrich). Enriched Terms are displayed as columns and input genes as rows. The genes associated with a term are indicated as red boxes in the matrix.

By performing similar GSEA using ShinyGO tool, we observed a significant enrichment of genes associated with pancreatic cancer (hsa05212), microRNAs in cancer (hsa05206), cell cycle (hsa04110), breast cancer (hsa05224), proteoglycans in cancer (hsa05205), chemokine signaling pathway (hsa04062), pathways in cancer (hsa05200), focal adhesion (hsa04510) and different signaling pathways (hsa04015 Rap1, hsa04010 MAPK, hsa04014 Ras and hsa04151 PI3K-Akt (Fig. 3). These enrichments are mostly related with genes belonging to the RAC, PLC and RAS families.

**Fig. 4:**
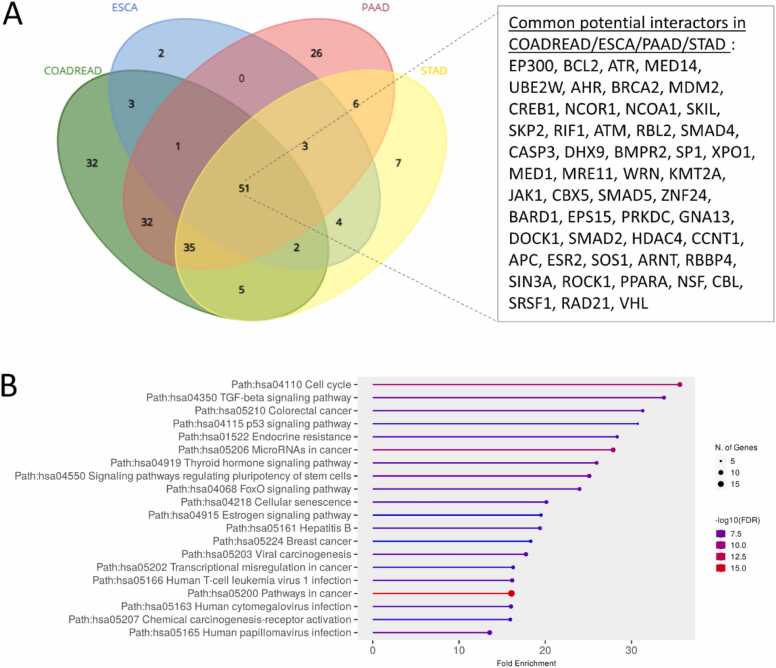
Venn diagram of *bona fide* TRPM7 interactors that are co-expressed in TCGA datasets and ontology analysis. (**A**) Venn diagram of coexpressed/predicted TRPM7 interactors in esophageal, gastric, colorectal and pancreatic cancers (ESCA, STAD, COADREAD and PAAD). Common genes are enlisted on the right panel. (**B**) Dot plot of Gene-set enrichment analysis using ShinyGO v0.81 tool.

The mRNA expression information can be combined with protein-protein interaction network analysis in order to identify *bona fide* partner. Indeed, physical interactions require co-expression of both proteins in the same cell or in the surrounding cells (for paracrine interaction). To this aim, we retrieved quantification data from RNAseq (RSEM) for TRPM7 using cbioportal in every TCGA datasets available and identified a list of genes that are correlated with TRPM7 (Spearman correlation, corrected q value <0.05) (Table S2). We observed approximatively 100 potential interactors in most of the TCGA datasets. Interestingly, we observed 51 common predicted and correlated interactors in digestive cancers, including esophageal, gastric, pancreatic and colorectal cancers (ESCA, STAD, PAAD and COADREAD) (Fig. 4A). GSEA using ShinyGO tool showed significant enrichments of KEGG pathways such as cell cycle (hsa04110), TGFβ, p53, thyroid hormone, FoxO, estrogen signaling pathways (hsa04350, hsa04115, has04919, hsa04068, 04915, hsa05200), cellular senescence (hsa04218) (Fig. 4B).

### 3.3 Predicted interactors that also have experimental evidence: could these be new candidates for TRPM7 interaction?

**Fig. 5:**
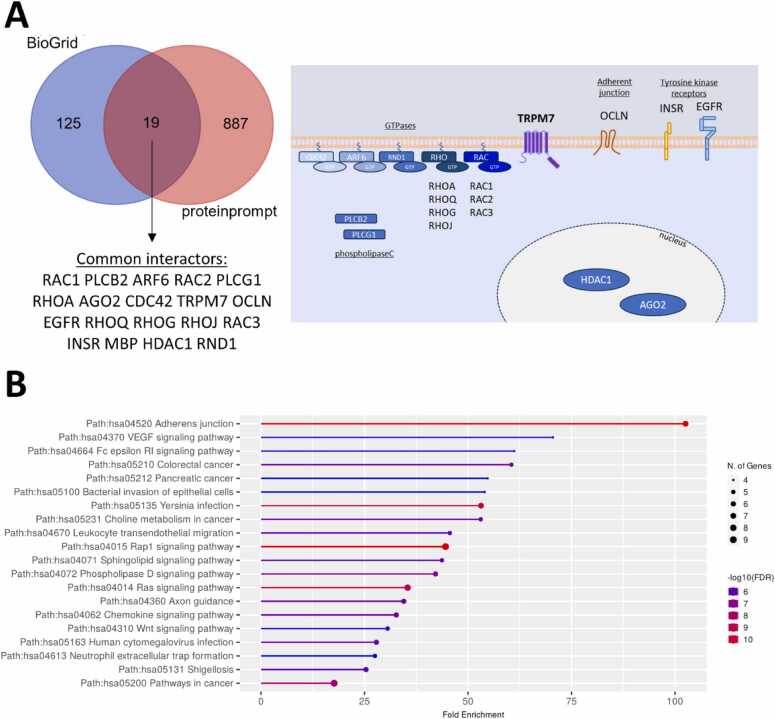
Venn diagram of *bona fide* TRPM7 interactors and ontology analysis. (**A**) Venn diagram of 144 TRPM7 interactors with experimental evidence (from BioGRID database) and 906 predicted interactors (from ProteinPrompt analysis) showed 19 common genes illustrated on the right panel. (**B**) Dot plot of Gene-set enrichment analysis using ShinyGO v0.81 tool.

We observed 19 predicted interactors that also harbored experimental evidences (illustrated in Fig. 5A). Interestingly, we found 7 different GTPases and 2 phospholipase C proteins. We also highlighted the interaction between TRPM7 and two tyrosine kinase receptors (EGFR and INSR). Their downstream signaling pathways play major roles in cancer by regulating biological processes such as proliferation, survival, differentiation, and metastasis. Altogether this reinforces the mechanistic link between TRPM7 and cell signaling. Finally, TRPM7 also interacts with occludin that is a component of cell junction. Its alteration is commonly associated with increased epithelial–mesenchymal transition (EMT) and tumor progression. Surprisingly, TRPM7 was shown to interact with nuclear proteins HDAC1 and AGO2. In their previous work, Krapinvinsky *et al.* have already identified nuclear proteins as interactors of TRPM7 cleaved kinase fragments (M7CKs) [50]. HDAC1 may interact with M7CKs as we observed a fair interaction ProteinPrompt score (0.6067) with restricted amino acid sequence corresponding to the alpha kinase domain (AA1594–1824). We hypothesize that TRPM7 could act as a connector between signaling pathways and gene silencing machinery. This remains to be fully deciphered in future investigations.

## 4 Perspectives in drug discovery

TRPM7 has been implicated in numerous diseases, including cancer. Its dual function as a cation channel and kinase, as well as its ubiquitous nature, complicate its multiple interactions with proteins involved in signal transduction and the regulation of cellular properties. Targeting the TRPM7 protein appears difficult given its essential role in regulating intracellular magnesium homeostasis and the current lack of specific pharmacological blockers. However, the anticancer agent CCT128930 has been recently identified as a potent inhibitor of TRPM7 channels [21], [109]. Many TRPM7 interactors in the BioGRID database were initially found in large scale mass spectrometry experiments and still require specific evidences. Moreover, ProteinPrompt prediction analysis may help to prioritize subset of novel interactors for experimental validation. Therefore, a better understanding of TRPM7's interaction partners and the underlying intracellular signaling mechanisms would enable the development of new therapeutic strategies based on the destabilization of these protein complexes or by use of bispecific antibodies allowing the direct targeting of particular cell types. Several GTPase-modulating drugs have been described recently. Notably KRAS drugs have been developed. Sotorasib is a potent and highly selective inhibitor of KRAS^G12C^ and is used in clinical trials for lung cancer harboring this mutation. Several works also focused on strategy of blocking interaction between KRAS and its partners. Deltarasin is blocking KRAS–PDEδ interaction and alter location of KRAS [110]). Finally, BI-2852 blocks the interaction KRAS with GEF effector [111]. Combination of such small GTPase inhibitors with TRPM7 blockers may be proposed as a promising therapeutical strategy to target the oncoprotein complexes involving TRPM7 in cancer cells.

Moreover, protein-protein interaction inhibitors disrupt critical molecular interfaces that regulate oncogenic signaling and associated cancer cell aggressiveness [112]. In order to develop such drugs (chemical compound/peptide) targeting TRPM7 interactions, further biochemical evidences, notably knowledge of the protein structure of both partner at the cell membrane as well as the interaction surface need to be obtained.

## 5 Concluding remarks

In this work, we have compiled an inventory of the numerous interaction partners of TRPM7, revealing the diversity and complexity of the mechanisms involving this protein. The use of computational and modeling tools has enabled us to identify new potential partners, opening up new therapeutic prospects for the development of drugs targeting these intracellular signaling protein complexes.

## CRediT authorship contribution statement

**Nicolas Jonckheere:** Writing – review & editing, Writing – original draft, Methodology, Funding acquisition, Formal analysis, Data curation, Conceptualization. **Lise Rodat-Despoix:** Writing – review & editing, Writing – original draft. **GAUTIER Mathieu:** Writing – review & editing, Writing – original draft, Supervision, Project administration, Funding acquisition, Conceptualization. **Frédéric Hague:** Writing – review & editing. **Isabelle Dhennin-Duthille:** Writing – review & editing. **Alban Girault:** Writing – review & editing.

## Author’s statements

NJ did the BioGRID and Proteinpromt analyses and the figures, and wrote the paragraphs 3 and 4; LRD wrote the paragraphs 2j and 2 n; IDD wrote the paragraphs 2d; AG and FH proofread and corrected the manuscript; MG coordinated the work and wrote the manuscript. All the authors corrected the manuscript.

## Funding

This work has been founded by The French National Cancer Institute (INCA) (PLBIO2024 grant INCa-19414), by the Cancéropôle Nord-Ouest (CNO), by the Ligue Contre le Cancer (Septentrion and Somme’s committees), and by the MOSOPS project. The MOSOPS project has received financial support from the French State, Hauts-de-France region, INSERM and A2U Alliance’s Universities. This work was also supported by grants from Contrat de Plan Etat-Région CPER Cancer 2015-2020 and Oncolille Institute.

## Declaration of Competing interest

The authors declare that they have no known competing financial interests or personal relationships that could have appeared to influence the work reported in this paper.
