# Supplementary material for: Unlocking TRPM7 interactions: A database-driven quest
Source: Comput Struct Biotechnol J. 2025 Nov 16;27:5375–90. doi: 10.1016/j.csbj.2025.11.030 (PMC12699267; doi:10.1016/j.csbj.2025.11.030)
Supplement: Table S1 — Supplementary material [file mmc1.docx]

Special Issue: “Mechanistic insights into membrane protein dynamics and allostery: implications in drug discovery”

TITLE: Unlocking TRPM7 Interactions: A Database-Driven Quest

Nicolas Jonckheere^1🖂^, Lise Rodat-Despoix^2^, Isabelle Dhennin-Duthille^2^, Alban Girault^2^, Frédéric Hague^2^, Mathieu Gautier^2🖂^

Affiliations:

^1^Univ. Lille, CNRS, Inserm, CHU Lille, UMR9020-U1277—CANTHER—Cancer Heterogeneity Plasticity and Resistance to Therapies, F-59000 Lille, France.

^2^Université de Picardie Jules Verne, UR-UPJV 4667, F-80000 Amiens, France.

^🖂^ To whom correspondence should be addressed:

nicolas.jonckheere@inserm.fr

mathieu.gautier@u-picardie.fr

Abstract:

Transient receptor potential cation channel subfamily M member 7 (TRPM7) is a dual function protein comprising a non-selective cation channel and an atypical kinase domain. TRPM7 has been involved in many diseases including malignancies. Indeed, TRPM7 is proposed as a promising target for therapeutical drug design. Numerous studies have shown that TRPM7 interacts with proteins involved in regulating intracellular signaling. Therefore, a better understanding of the TRPM7 interactome would provide insight into pathophysiological mechanisms at the cellular and molecular levels. It could also open up new therapeutic avenues for molecules targeting either the proteins of interest directly or protein-protein interactions.

In the first part of this work, we present the interaction partners described in the literature for TRPM7 and their potential impacts on cell biology. In the second part of the manuscript, we use public databases and protein interaction modeling tools to characterize the TRPM7 interactome. In particular, the analysis of the TRPM7 interactome using experimental data (BioGRID) and modeling tools (ProteinPrompt) has allowed us to isolate 19 genes of interest mainly related to small GTPase pathways involved in digestive neoplasia such as colorectal and pancreatic cancers.

In summary, we provide an extended overview of potential TRPM7 interactors which need to be validated in cellular models. This will provide crucial insights into the molecular mechanisms at the tumor cell membrane, helping us to propose new therapeutic targets for precision medicine.

Keywords: TRPM7, protein interactome, BioGRID database, GSEA.

**1. Introduction:**

Transient receptor potential cation channel subfamily M member 7 (TRPM7), also named CHAK1, LTRPC7, TRP-PLIK, is a dual function protein comprising a non-selective cation channel and an atypical kinase domain [1-3]. The TRPM7 subunit, a 212 kDa protein containing 1865 amino acids, can assemble as homotetramers to form a functional ion channel. But TRPM7 can also associate with TRPM6 subunits to form heterotetramers [4]. TRPM7 is ubiquitously expressed in human tissues and is required for cell viability and embryogenesis [5-7]. TRPM7 physiological roles are mainly due to its channel function. Thus, TRPM7 is essential for Ca^2+^, Mg^2+^ and Zn^2+^ intestinal absorption, renal reabsorption [8], and more generally for cellular Mg^2+^ homeostasis regulation [9]. Electrophysiological experiments conducted on HEK293 and CHOK1 cells expressing TRPM7 have shown that the channel is also permeant for other metal divalent cations including Ni^2+^, Mn^2+^, Co^2+^, Cd^2+^ [10, 11], suggesting that TRPM7 could be involved in contamination by toxic metal traces. TRPM7 is involved in some pathologies related to disruption of Mg^2+^ homeostasis, including hypomagnesemia, macrothrombocytopenia, and trigeminal neuralgia [12]. Moreover, TRPM7 is overexpressed in many cancers where it regulates cell proliferation, migration and invasion through its channel function, but which also involves its kinase domain [13].

TRPM7 is a chanzyme due to its kinase domain located in the intracellular C-terminus. TRPM7 kinase domain is a serine/threonine kinase belonging to the α-kinase family [3, 14, 15]. In addition to TRPM6 and TRPM7, the α-kinase family includes the *Dictyostelium* Myosin Heavy Chain (MHC) kinases A, B, and C, and elongation factor-2 kinase (eEF2k) [14, 16]. In contrast to conventional protein kinases, α-kinases may phosphorylate on serine/threonine residues in α-helical conformations [16]. The crystal structure of the TRPM7 kinase domain has been determined by Yamaguchi *et al.* in 2001 [17]. Each kinase domain monomer is composed of 6 α-helices and 15 β-strands. The central catalytic core of the TRPM7 kinase domain has structural similarities to the classical protein kinase family including cAMP dependent protein kinase (PKA) [17]. In particular, the kinase domain displayed a conserved lysine residue at 1648 position which is absolutely required for catalytic activity, a C-terminal glycine-rich motif which may be involved in peptide substate recognition, and a Zn^2+^ binding module located in the C-terminal lobe which regulates kinase structure stability [17]. Regarding the mechanisms of TRPM7 channel gating, high-resolution structures of closed TRPM7 channels were resolved by cryo-electron microscopy (cryo-EM) [18]. By using a combination of site-directed mutagenesis, electrophysiological recordings, and modelling and molecular dynamics (MD) simulations, Schmidt *et al.* identified a pivotal Mg^2+^ regulatory site located within the lower channel gate at the position N1097, stabilizing the TRPM7 channel in the closed state [19]. They also identify the gain-of-function mutation N1098Q that promotes constitutively opened TRPM7 channel [19, 20]. Similarly, the resolutions of TRPM7 structures in closed and opened states have been made to determine the mechanisms of channel activation and inhibition by pharmacological opener (naltriben) or blockers (NS8593 and VER155008), respectively [20]. This study showed that naltriben stabilizes TRPM7 channel in the open conducting state by tying and pooling the N-terminal domains of the neighboring subunits leading to iris-like transformation of the cytoplasmic domain. Moreover, a vanilloid-type site involved in the stabilization of the closed state by NS8593 and VER155008 has been identified in the TRPM7 structure. Finaly, this vanilloid-type site is also involved in the TRPM7 inhibition by the anticancer agent CCT128930 suggesting a common mechanism for TRPM7 inhibition by pharmacological agents [21]. Interestingly, the TRPM7 kinase domain is regulated by divalent cations. Indeed, using protein phosphorylation assays on Myelin Basic Protein (MBP) and histone H3 as substrates of the purified TRPM7 kinase, Ryazanova *et al.*, showed that Mg^2+^ and Mn^2+^ are required as enzymatic cofactors [3]. Mn^2+^ stimulates TRPM7 kinase activity twice as much as Mg^2+^, while Zn^2+^ and Co^2+^ have an inhibitory effect. In the other hand, Ca^2+^ has no effect on the TRPM7 kinase activity [3]. Under physiological conditions, only intracellular Mg^2+^ divalent cations can regulate the TRPM7 kinase activity [3]. Contrarily to the channel function, the role of the kinase domain is largely unknown. The use of genetically modified mice showed that deletion of the kinase domain induced embryonic lethality identical to full TRPM7 KO mutant [9]. Interestingly, the heterozygous chimeric mice have an altered Mg^2+^ regulation and a lower survival rate when placed on Mg^2+^-deficient diets [9]. On the other hand, the K1648R mutation resulting on non-functional kinase did not induce any lethality nor channel function impairment, and improve survival in mice receiving Mg^2+^-deficient diets [22, 23]. Taken together, these data suggest that although the presence of the TRPM7 kinase domain is required for survival and Mg^2+^ regulation, its phosphotransferase function is not essential. It is possible that TRPM7 exerts biological functions by protein-protein interactions. In this review, the first part will recapitulate the known interactors of TRPM7 and the biological functions of these complexes. The aim of the second part is to propose new TRPM7 interactors by using online databases and computing tools.

**2. Known interaction partners of TRPM7:**

a. AKT

AKT is a serine/threonine kinase activated downstream of phosphoinositide-3-kinase (PI3-K). PI3-K/AKT pathway is frequently dysregulated in numerous malignancies. By using an *in vitro* TRPM7 kinase assay, Hoeger *et al.* showed that AKT1 is a direct substrate of recombinant purified TRPM7 kinase [24]. In chronic myeloid leukemia (CML) cells, they showed that TRPM7 kinase regulates AKT and mothers against decapentaplegic homolog 2 (SMAD2) pathways leading to modulated cyclooxygenase-2 (COX-2) expression [24]. Thus, as a direct AKT-activating kinase, these findings reinforced the importance of TRPM7 in the regulation of oncogenic signaling pathways.

b. Annexin-1

Annexin-1 operates through interaction with membrane phospholipids in a Ca^2+^-dependent manner leading to numerous signaling pathways involved in inflammation, cell proliferation, differentiation and apoptosis. For example, Annexin-1 can bind with S100 EF-hand Ca^2+^-binding proteins to form complexes promoting membrane crosslink [25]. In 2004, Dorovkov and Ryazanov showed that Annexin-1 may be phosphorylated by the recombinant TRPM7 kinase at a conserved serine residue (Ser5) located within the N-terminal amphipathic α-helix [26]. Furthermore, they highlighted that N-terminal Annexin-1 peptide phosphorylation by TRPM7 impedes the capacity of Annexin-1 peptide to adopt a α-helicoidal conformation, to interact with membranes, and to bind with S100A11 protein [27]. Interestingly, Annexin expression has been found to be altered in many cancers, mainly resulting in a loss of expression in tumor tissue [25]. However, the interaction between Annexin-1 and TRPM7 has not yet been demonstrated in cancer cells, nor in preclinical models.

c. Ca^2+^-binding proteins: Calmodulin and S100A1

It has been also shown that EF-hand Ca^2+^ binding proteins may bind directly to TRPM7 through its N-terminal region. In particular, Bousova *et al.* identified binding sites for calmodulin and S100A1 within the TRPM7 N-terminal region [28]. In this study, a peptide corresponding to the TRPM7 N-terminal region has been synthetized and the interactions with calmodulin and S100A1 were investigated by *in vitro* fluorescence anisotropy and by *in silico* molecular modeling [28]. Both calmodulin and S100A1 are able to form complexes with TRPM7 N-terminal intracellular region at position T523-L535 with dissociation constants in the micromolar level ranges [28]. Nevertheless, this mechanism has not yet been clearly demonstrated at the cellular or whole animal level. Mishra *et al.* showed that TRPM7-like currents are inhibited by increasing cytosolic Ca^2+^ in rat hepatocytes and hepatoma cells [29]. In particular, the study demonstrated that Ca^2+^-inhibition of TRPM7-like currents is partially mediated by the Ca^2+^/calmodulin-dependent protein kinase II (CaMKII) activity. Interestingly, a CaMKII target sequence has been identified within the COOH-terminal domain of rat TRPM7. On the other hand, no consensus sequence for a calmodulin binding site has been revealed, suggesting that calmodulin and Ca^2+^ act indirectly through CaMKII rather than *via* a direct interaction with the channel. TRPM7 has been involved in neuronal death in a mouse model of hypoxic-ischemic brain cell death [30]. Waixenicin A has been used as a potential inhibitor of TRPM7 to prevent brain injury. By proteomic analysis and western-blots, it has been showed that the waixenicin treatment prevented the reduced CaMKII expression induced by brain injury. This study suggests that, CaMKII is activated by Ca^2+^ influx through TRPM7 channels. Taken together, the findings of these studies did not reveal any evidence of direct interaction between Ca^2+^-dependent proteins and TRPM7 channels.

d. CNNMs, PRL-1 and ARL15

TRPM7 is the major regulator of Mg^2+^ homeostasis in mammals [8, 9]. However, there are several Mg^2+^ channels and transporters which have been identified but their functions are not yet fully understood [31]. Mg^2+^ is equally distributed on both intra- and extracellular compartments, resulting in a lower electrochemical force for membrane diffusion of this cation compared to other cations such as Na^+^ or Ca^2+^. Interestingly, two groups parallelly described a new mechanism of Mg^2+^ inflow through TRPM7 involving interaction with CNNM (Cyclin And CBS Domain Divalent Metal Cation Transport Mediator) proteins [32, 33]. CNNM are transmembrane proteins containing a N-terminal extracellular domain, a transmembrane domain, and two intracellular domains which are a CBS-pair domain (or Bateman domain) and a Cyclic Nucleotide-Binding Homology (CNBH) domain [34, 35]. CNNM have been identified as Mg^2+^ transporters but their role in Mg^2+^ inflow or lowering by extrusion is still under debate [31, 36]. Mass spectroscopy analysis on purified endogenous TRPM7 from rodent brain or on overexpressed TRPM7 in HEK-293T cells identified CNNMs, Phosphatase of Regenerating Liver 1 (PRL-1), and ADP-ribosylation factor-like GTPase 15 (ARL15) as potential interactors [32, 33]. Interaction between TRPM7 and CNNMs was confirmed by co-immunoprecipitation assay (for CNNM1, 2, 3 and 4) and by proximity-dependent biotin identification (BioID) (for CNNM3). Moreover, KO of *CNNM3* or *CNNM4* significantly reduced TRPM7-dependent Mg^2+^ entry, and decreased TRPM7 currents recorded by whole-cell patch-clamp. On the other hand, the Mg^2+^-lowering activities of CNNM occur independently of TRPM7 [32]. Functional co-expression in heterologous expression system, using WT or kinase dead TRPM7, showed that ARL15 strongly affects TRPM7 channel function, while CNNM3 appears to acts as a negative regulator of TRPM7 kinase activity [33].

It has been shown that TRPM7 and CNNM form a trimeric complex with ARL15 [32, 33, 37]. ARL15 binds specifically to CNNM CBS-pair domain and inhibits CNMM and TRPM7 Mg^2+^ transport [34, 37]. Tetteh *et al.* went further inside TRPM7 regulation by CNNMs by showing that CNNM2 CBS-pair domain modulates TRPM7 channel activity through the action of ARL15, whereas CNNM2 CNBH domain binds to TRPM7 kinase domain and enhanced its catalytic activity *in vitro* [38]. Importantly, ARL15 and PRL compete for CNNM binding, regulating CNNM-TRPM7 interaction and Mg^2+^ homeostasis [34]. PRL2 overexpression counteracts ARL15 binding to CNNM3 and enhances TRPM7 function in a magnesium-dependent mechanism, suggesting that PRL2 acts as a magnesium biosensor controlling the CNNM3/TRPM7 interaction [39]. Jolly *et al.* recently proposed two models based on direct interaction between TRPM7 and CNNMs with ARL15/PRL as modulators of CNNM, or indirect interaction through ARL15 [36].

Interestingly, PRL and CNNM have been documented in colorectal and breast cancers. Funato *et al.* showed that PRL3 binds to CNNM4 leading to the inhibition of Mg^2+^ efflux. High Mg^2+^ intracellular levels promote cell energy metabolism and cell proliferation through AMPK/mTOR pathway. Moreover, they showed an overexpression of PRL3 and a downregulation of CNNM4 in colorectal cancers suggesting that CNNM4 prevents tumor progression by controlling low Mg^2+^ intracellular levels and by regulating energy metabolism [40]. Hardy *et al.* showed that PRL2 is overexpressed in human breast cancer tissues and in metastatic lymph nodes, and that PRL2 knockdown decreases cell migration of MDA-MB-231 and mouse mammary tumor-derived cell lines, as well as tumor progression in mice models [41]. They further showed that PRL2 binds to CNNM3 to promote Mg^2+^ entry leading to cell proliferation and tumor growth. Importantly, CNNM3 expression was positively correlated with PRL2 and Ki-67 expressions in human breast cancer tissues [42]. Moreover, CNNMs expression was analyzed in digestive cancers using Genotype Tissue Expression (GTEx) and The Cancer Genome Atlas (TCGA) datasets [43]. CNNM3 is overexpressed in esophageal carcinoma whereas CNNM2 is overexpressed in esophageal and stomach cancers. On the other hand, an overexpression of CNNM4 was observed in esophageal, stomach, pancreatic, colon and rectal cancers. The high expression of CNNM4 is also correlated with high expression of TRPM7 and MAGT1, another magnesium transporter, and this TRPM7/MAGT1/CNNM4 signature is associated with low patient survival in pancreatic adenocarcinoma [43].

The PRL/ARL/CNNM/TRPM complex, recently named the PACT complex [36], has been well described in heterologous overexpression systems and only few studies demonstrated the involvement of PRL and CNNM in cancer. Given that TRPM7 is overexpressed in numerous types of cancers, and involved in cell proliferation, migration and invasion, it is tempting to speculate that the PACT complex may play a significant role in cancer cell fates by regulating their intracellular Mg^2+^ levels.

e. CREB

Song *et al.* showed that the cAMP Response Element Binding protein (CREB) is an ideal substate of TRPM7 for studying kinase activity by LANCE *Ultra* assay [44]. The ability of purified TRPM7 kinase domain to phosphorylate the CREB peptide led to the identification of TG100-115 as a new inhibitor of TRPM7 kinase activity and breast cancer cell migration [44]. The interaction between CREB and TRPM7 has been confirmed in dental cells. By using a model of TRPM7 kinase-inactive knock-in mutant mice that have normal TRPM7 ion channel functions (TRPM7 KR mice), Ogata *et al.* showed a lower expression of phosphorylated CREB in incisors of TRPM7 KR mice compared to control mice [45]. Moreover, co-immunoprecipitation experiments revealed that TRPM7 and CREB may interact in ameloblast-lineage cells [45]. In cancer and endothelial cells, TRPM7 is essential for cellular glycolysis regulation through CREB-dependent pathway [46]. However, in this study of Wu *et al.*, TRPM7 regulated CREB phosphorylation in an indirect manner, *via* Ca^2+^ influx-induced calcineurin activation [46].

f. eEF2-k

Eukaryotic elongation factor 2 (eEF2) plays a central role in protein translation by binding to ribosomes. eEF2 is phosphorylated by its kinase eEF2-k, leading to its failure to bind ribosomes. Perraud *et al.* showed that eEF2 phosphorylation is increased under low Mg^2+^ conditions [47]. By GST pulldown assays, they showed that TRPM7 interacts with eEF2-k but not eEF2 to adapt cell metabolism to Mg^2+^ availability [47].

g. EGFR

Co-immunoprecipitation experiments, proximity ligation assays (PLA) and confocal microscopy showed an interaction between TRPM7 and epidermal growth factor (EGF) receptor (EGFR) in primary vascular smooth muscle cells (VSMC) isolated from rat mesenteric arteries [48]. From a mechanistic point of view, EGF stimulated TRPM7-EGFR interactions in a c-Src-dependent manner, and EGF/EGFR phosphorylated TRPM7 leading to enhanced VSMC proliferation and migration [48]. Interestingly, TRPM7 acted both upstream and downstream of EGF/EGFR pathway because EGFR expression was downregulated in TRPM7 deficient mice leading to vascular remodeling with aortic wall thinning [48].

h. Histone H3

Histone H3 was one of the first TRPM7 substrate to be identified by using the purified C-terminal part of the protein containing kinase catalytic domain expressed in bacteria [3]. In SV40 MES 13 mouse mesangial cell line, Clapham’s group showed that TRPM7 kinase domain may be cleaved from the transmembrane channel by caspase to form TRPM7 cleaved kinase fragments (M7CKs) [49, 50]. These M7CKs translocated in the nucleus where they bound to nuclear proteins including transcription factors (*see section k*). Interaction between M7CKs and nuclear proteins led to histone H3 at Ser10 and Ser28 phosphorylation which is essential for transcription regulation, DNA repair, and mitotic chromatin condensation [50]. In this model, TRPM7 channel function regulated Zn^2+^ influx which is required for interaction between M7CKs and nuclear proteins containing zinc-finger [50]. Indeed, both channel and kinase functions seem necessary to regulate TRPM7-dependent gene regulation [50].

i. Myelin Basic Protein

As for histone H3, Myelin Basic Protein (MBP) has been identified as TRPM7 substrate for its kinase activity [2, 3, 51]. Nevertheless, the physiological significance of interaction between TRPM7 and MBP has not been yet elucidated.

j. Myosin

Through three complementary studies, van Leeuwen's group has endeavored to decipher the mechanisms by which TRPM7 regulates the assembly and stability of myosin filaments, and consequently the contraction of actomyosin fibers in adhesion processes. Based on the fact that TRPM7 (as TRPM6) encode channel-kinases, they hypothesized that the coupling of a cation channel to an α-kinase in a single polypeptide could explain the close relationship between Ca^2+^ signaling and Ca^2+^-dependent actomyosin remodeling. They firstly established that activation of TRPM7 by bradykinin (BK), a Gq-PLC-coupled receptor agonist that induces Ca^2+^-dependent phosphorylation of the Myosin Heavy Chain (MHC) and promotes actomyosin relaxation in mouse N1E-115 neuroblastoma cells [52], leads to a Ca^2+^- and kinase-dependent interaction with the actomyosin cytoskeleton. Accordingly, overexpression of TRPM7 increases intracellular Ca^2+^ levels accompanied by cell spreading, adhesion and the formation of focal adhesions. Activation of TRPM7 induces the transformation of these focal adhesions into podosomes by a kinase-dependent mechanism, whereas cells expressing kinase-dead TRPM7 failed to induce podosomes in response to BK stimulation. Moreover, they demonstrate that TRPM7 interacts with myosin IIA through its COOH-terminus and requires an active kinase domain [53]. They next demonstrated that TRPM7 directly phosphorylates the mouse and human heavy chain of non-muscle myosin IIA, specifically targeting a short stretch of amino acids within its α-helical tail. This phosphorylation regulates myosin IIA filament stability and cortical localization, thereby influencing cytoskeletal dynamics and cellular contractility [54]. This property could also be found in TRPM6 subtype. While TRPM6 and TRPM7 share electrophysiological properties and cellular functions, these channels are non-redundant genes raising the possibility that the kinases have distinct substrates. In that context, they demonstrate that TRPM6 and TRPM7 phosphorylate the assembly domain of myosin IIA, IIB and IIC on identical residues. Whereas phosphorylation of myosin IIA is restricted to the coiled-coil domain, TRPM6 and TRPM7 also phosphorylate the non-helical tails of myosin IIB and IIC [55].

k. Nuclear proteins

The detection of M7CKs which were specifically localized in the nucleus prompted the investigation of their potential interaction with nuclear proteins [50]. Krapivinsky *et al.* identified RYBP, Ruvbl1/pontin, Ruvbl2/reptin, DDX3X, DDB1, and DBC1 as TRPM7 potential interactors in a yeast-two hybrid (Y2H) screen of a rat brain library and by using tandem affinity purification with C-terminal TRPM7 fragment [50, 56]. These results were confirmed by co-immunoprecipitation experiments in HEK293T cell co-expressing system, and by GST-pulldown assays [50]. Although TRPM7 kinase interacts with these nuclear proteins, it does not directly phosphorylate them [50].

l. PAK1

Our recent data showed that TRPM7 interacts with the serine/threonine-protein kinase PAK1 in PANC-1 pancreatic cancer cell line by using co-immunoprecipitation and PLA experiments [57]. PAK1 is a protein kinase involved in intracellular signaling by regulating small GTPase activation that plays an important role in cell adhesion, migration and invasion. The treatment with TG100-115 abolished the formation of PLA complexes which strongly suggests that TRPM7 kinase activity regulates the interaction between PAK1 and TRPM7 in PANC-1 cells [57]. Moreover, the endogenous deletion of TRPM7 kinase domain led to PANC-1 cell migration impairment, epithelial-like phenotype maintaining, and abolition of tumor growth in a model of mouse subcutaneous xenograft [57].

m. PLC

In 2002, Clapham’s laboratory identified a 146-amino-acid carboxy-terminal segment of the TRPM7 kinase (PLIK; phospholipase C interacting kinase) domain in a Y2H screen of a rat brain library. By GST-pulldown assays, they demonstrated that the PLIK domain of TRPM7 directly interacts with the C2 domain of phospholipase C (PLC-β1,2,3 and γ1) from HEK-293T cells transiently expressing constructs (TRPM7 and PLC-β1–4, PLC-γ1 or PLC-δ1) [58]. Through this interaction, TRPM7 can modulate PLC signaling pathways, thereby affecting PIP₂ metabolism and subsequent Ca²⁺ mobilization. TRPM7 channels are highly permeable to Ca^2+^, and Runnels and collaborators demonstrated that the TRPM7 COOH‐terminus associates with phospholipase C (PLC) isoforms and that PLC activation regulates TRPM7 channel opening [2, 58, 59]. For instance, in platelets, alterations in TRPM7 kinase activity have been linked to impaired PIP₂ metabolism, leading to reduced Ca²⁺ responses upon stimulation of major platelet receptors [60]*.* The results of these various studies lead to the conclusion that the PLC substrate phosphatidylinositol 4,5-bisphosphate (PIP_2_) is a key regulator of the TRPM7 signaling.

n. RhoA

Additionally, TRPM7 has been implicated in the regulation of RhoA activity by Gudermann’s group. Their work established that, treatment during 24h at 30µM by NS8593 (a TRPM7 inhibitor) or TRPM7 knock-down of human hepatocyte carcinoma cell line HuH7, decreases RhoA activity by 50% and drastically reduces both the TRPM7/RhoA interaction and proximity [61]. They set out to further dissect the underlying molecular mechanisms of this interaction by using recombinant TRPM7 kinase and Histone-tagged RhoA to confirm that TRPM7 kinase phosphorylated RhoA at Ser188 in a dose-dependent manner [61]. As a matter of fact, they demonstrated that through its functional coupling *via* its kinase domain, TRPM7 modulates the actomyosin cytoskeleton by affecting RhoA signaling pathways, which are crucial for various cellular processes, including migration and adhesion. Recently, our group confirmed that RhoA co-immunoprecipitates with TRPM7 in a kinase-dependent manner in human pancreatic ductal adenocarcinoma cell line PANC-1 [57].

o. SMAD2

Mothers against decapentaplegic homolog 2 (SMAD2) protein is an intracellular signal transducer and a transcriptional regulator activated by TGF-β leading to cell activation or differentiation. Romagnani *et al.* showed that TRPM7 regulates TGF-β/SMAD2 signaling pathway in mouse T cells [62]. By *in vitro* kinase assay, their work highlighted that TRPM7 kinase domain directly phosphorylates SMAD2 at the C-terminal Ser465/467 motif. Moreover, they identified close proximity between TRPM7 and SMAD2 in T cells by using PLA assays. These results showed that TRPM7 regulates T cell differentiation by direct phosphorylation of SMAD2 leading to its translocation into the nucleus [62].

p. STIM2

Faouzi *et al.* showed that the TRPM7 kinase domain is required for the regulation of Store-Operated Calcium Entry (SOCE) in DT40 B-lymphocytes [63]. They showed that TRPM7 channel function contributes to intracellular Ca^2+^ homeostasis by filling the intracellular Ca^2+^ stores. They further showed that SOCE was similarly inhibited in cells overexpressing TRPM7 mutants with kinase domain deletion (Δ-kinase) or inactivation (K1648R), indicating that the TRPM7 kinase domain regulates SOCE in these cells. STIM2 has been suggested as a possible partner of TRPM7 because TRPM7 inhibition by NS8595 did not inhibit SOCE in STIM2-KO cells unlike Orai1-KO and Orai2-KO. However, a direct interaction between STIM and TRPM7 proteins remains to be determined.

q. Synaptic vesicle proteins: synaptotagmin I, synapsin I, and snapin

Krapivinsky *et al.* identified the presence of TRPM7 in the membrane of synaptic vesicles of sympathetic neurons [56]. Moreover, they showed that snapin protein is a potential interactor of TRPM7 by Y2H screening of a rat brain library. Snapin is highly enriched in synaptic vesicles and its phosphorylation stimulates synaptic transmission. By co-immunoprecipitation experiments, they identified synaptotagmin I and synapsin I as additional interactors of TRPM7 in rat synaptic vesicles. In a physiological point of view, TRPM7 downregulation led to acetylcholine release impairment [56].

r. Tropomodulin

Tropomodulin 1 has been identified as a potential substrate of the TRPM7 kinase domain [64]. The phosphorylation sites are located in the N-terminal domain including serine and threonine residues in the tropomyosin-binding and actin-capping regions. Thus, it has been hypothesized that the tropomodulin phosphorylation by TRPM7 kinase may regulate the dynamics of actin filaments.

s. TRPM6/TRPM7

*TRPM6* is the *TRPM7* gene paralog [65]. TRPM6 can assemble with TRPM7 to form heterotetramers whose expression is restricted to the intestine and to the distal convoluted tubule (DCT) of the kidney [66] where they facilitate Mg^2+^ cellular influx, and notably epithelial Mg^2+^ absorption and reabsorption [4]. The TRPM6 and TRPM7 subunits are their own substrates due to the presence of serine/threonine-rich domains upstream of the kinase domain [17]. Clark *et al.*, highlighted that massive autophosphorylation of TRPM7 increases kinase activity and substrate recognition [67]. Li *et al.* assessed the electrophysiological properties of TRPM6, TRPM7 and TRPM6/TRPM7 channels in heterologous expression systems (CHOK1 and HEK293 cells) and mouse distal convoluted tube (MDCT) cells [11]. They firstly demonstrated that the relative permeability to Ni^2+^ is significantly different among these channels with the following sequence: TRPM7>TRPM6/TRPM7>TRPM6. Secondly, they showed that inward currents through TRPM6/TRPM7 channels were more sensitive to low extracellular pH than for TRPM6 or TRPM7 channels. Thirdly, the single channel conductance of TRPM6 channel (83.6 pS) was larger than those of TRPM6/TRPM7 (56.6 pS) and TRPM7 (40.1 pS) channels. Finally, 500 µM of 2-aminoethoxydiphenyl borate (2-APB) dramatically increased TRPM6 currents while it decreased TRPM7 and only slightly increased TRPM6/TRPM7 currents. Thus, these channels may play different physiological roles depending of their expression profile.

**3. New insight from online protein interaction databases:**

a. Interactors with experimental evidences from BioGRID

We queried the BioGRID (Biological General Repository for Interaction Datasets) database for protein interactions with human TRPM7. BioGRID is an open-access database that records protein and genetic interactions from multiple species [68]. Protein interactions are determined by mass spectrometry (MS) techniques such as Affinity Capture-MS and Proximity Label-MS. Affinity Capture-MS is based on affinity captured on bait protein contained in cell extracts by polyclonal antibody or epitope tag. Proximity Label-MS method (such as BioID [69]) is based on bait-enzyme fusion protein that modify vicinal proteins. Affinity Capture-MS allows identification of partner in endogenous state (post-translational modification) in cells, and can also show multiprotein complex. BioID can also show transient interactions and is highly sensitive, but may require further biochemical validation. The affinity captured partners can also be validated by western-blots. The purification step is thought to get rid of potential contaminating proteins and provide high confidence interaction partners. Reconstituted Complex method, such as Glutathione-S-transferase (GST) pull-down, relies on recombinant proteins or cellular extracts with purified baits, and represents a more reliable biochemical method.

By querying the BioGRID database, we obtained the list of 144 interactors (Table 1). Low throughput strategies such as two hybrid methods led to identification of interaction between TRPM7 and phospholipase C proteins such as PLCB1, PLCB2 [58] or ITSN1 [70]. TRPM7, HIST3H3 and MBP were shown to be direct substrates of the TRPM7 kinase activity [3]. Moreover, 95 interactors were identified by high throughput Proximity Label MS. Similarly, 55 interactors have been identified by BioID. Several works performed unbiased interrogation of genetic interaction using CRISPR screening and led to identification of SCYL1, EGFR, KRAS, RIT1, FBXW7 and FASN as interactors [71-74]. However, these genetic interactions are based on dependency regarding cell survival or proliferation and are not necessarily mediated by direct physical interaction.

**Table 1:** List of TRPM7 interactors enlisted in the BioGRID database.

| Official Symbol Interactor A | Official Symbol Interactor B | Synonyms Interactor A | Experimental System | Experimental System Type | Source |
| --- | --- | --- | --- | --- | --- |
| PLCB2 | TRPM7 | PLC-beta-2 | Two-hybrid | physical | [58] |
| PLCB1 | TRPM7 | EIEE12\|PI-PLC\|PLC-154\|PLC-I\|PLC154\|PLCB1A\|PLCB1B | Two-hybrid | physical | [58] |
| TRPM7 | TRPM7 | ALSPDC\|CHAK\|CHAK1\|LTRPC7\|LTrpC-7\|TRP-PLIK | Biochemical Activity | physical | [3] |
| TRPM7 | HIST3H3 | ALSPDC\|CHAK\|CHAK1\|LTRPC7\|LTrpC-7\|TRP-PLIK | Biochemical Activity | physical | [3] |
| TRPM7 | MBP | ALSPDC\|CHAK\|CHAK1\|LTRPC7\|LTrpC-7\|TRP-PLIK | Biochemical Activity | physical | [3] |
| TRPM7 | PLCG1 | ALSPDC\|CHAK\|CHAK1\|LTRPC7\|LTrpC-7\|TRP-PLIK | Reconstituted Complex | physical | [58] |
| TRPM7 | PLCB1 | ALSPDC\|CHAK\|CHAK1\|LTRPC7\|LTrpC-7\|TRP-PLIK | Reconstituted Complex | physical | [58] |
| TRPM7 | PLCB2 | ALSPDC\|CHAK\|CHAK1\|LTRPC7\|LTrpC-7\|TRP-PLIK | Reconstituted Complex | physical | [58] |
| TRPM7 | PLCB3 | ALSPDC\|CHAK\|CHAK1\|LTRPC7\|LTrpC-7\|TRP-PLIK | Reconstituted Complex | physical | [58] |
| TRPM7 | PLCB2 | ALSPDC\|CHAK\|CHAK1\|LTRPC7\|LTrpC-7\|TRP-PLIK | Affinity Capture-Western | physical | [58] |
| SNAP29 | TRPM7 | CEDNIK\|SNAP-29 | Affinity Capture-MS | physical | [75] |
| ZACN | TRPM7 | L2\|LGICZ\|LGICZ1\|ZAC\|ZAC1 | Affinity Capture-MS | physical | [75] |
| TCTN3 | TRPM7 | C10orf61\|JBTS18\|OFD4\|TECT3 | Proximity Label-MS | physical | [76] |
| HDAC1 | TRPM7 | GON-10\|HD1\|RPD3\|RPD3L1 | Affinity Capture-MS | physical | [77] |
| RAB7A | TRPM7 | PRO2706\|RAB7 | Affinity Capture-MS | physical | [77] |
| VAPA | TRPM7 | VAP-33\|VAP-A\|VAP33\|hVAP-33 | Affinity Capture-MS | physical | [77] |
| Bub1 | TRPM7 | AL022991\|Bub1a\|C80208\|D2Xrf87 | Affinity Capture-MS | physical | [77] |
| C1qbp | TRPM7 | AA407365\|AA986492\|D11Wsu182e\|HABP1\|P32\|gC1qBP | Affinity Capture-MS | physical | [77] |
| Eef1a1 | TRPM7 | - | Affinity Capture-MS | physical | [77] |
| Flot1 | TRPM7 | reggie-2 | Affinity Capture-MS | physical | [77] |
| Timeless | TRPM7 | C77407\|Debt69\|tim | Affinity Capture-MS | physical | [77] |
| Tmed2 | TRPM7 | 1110032D12Rik\|1810020N21Rik\|Rnp24\|Sid394\|p24beta1 | Affinity Capture-MS | physical | [77] |
| Cep152 | TRPM7 | AI851464\|mKIAA0912 | Affinity Capture-MS | physical | [77] |
| ARL15 | TRPM7 | ARFRP2 | Affinity Capture-MS | physical | [78] |
| TEX28 | TRPM7 | CXorf2\|MRX99\|TEX28P1\|TEX28P2\|fTEX | Affinity Capture-MS | physical | [78] |
| SNAP29 | TRPM7 | CEDNIK\|SNAP-29 | Affinity Capture-MS | physical | [78] |
| PNLDC1 | TRPM7 | - | Affinity Capture-MS | physical | [78] |
| ZACN | TRPM7 | L2\|LGICZ\|LGICZ1\|ZAC\|ZAC1 | Affinity Capture-MS | physical | [78] |
| TRIM25 | TRPM7 | EFP\|RNF147\|Z147\|ZNF147 | Affinity Capture-RNA | physical | [79] |
| K8.1 | TRPM7 | - | Affinity Capture-MS | physical | [80] |
| SCYL1 | TRPM7 | GKLP\|NKTL\|NTKL\|P105\|TAPK\|TEIF\|TRAP | Negative Genetic | genetic | [71] |
| TRPM7 | TRPM7 | ALSPDC\|CHAK\|CHAK1\|LTRPC7\|LTrpC-7\|TRP-PLIK | Negative Genetic | genetic | [71] |
| HRAS | TRPM7 | C-BAS/HAS\|C-H-RAS\|C-HA-RAS1\|CTLO\|H-RASIDX\|HAMSV\|HRAS1\|RASH1\|p21ras | Proximity Label-MS | physical | [81] |
| KRAS | TRPM7 | C-K-RAS\|CFC2\|K-RAS2A\|K-RAS2B\|K-RAS4A\|K-RAS4B\|KI-RAS\|KRAS1\|KRAS2\|NS\|NS3\|RASK2 | Proximity Label-MS | physical | [81] |
| NRAS | TRPM7 | ALPS4\|CMNS\|N-ras\|NCMS\|NRAS1\|NS6 | Proximity Label-MS | physical | [81] |
| CANX | TRPM7 | CNX\|IP90\|P90 | Proximity Label-MS | physical | [82] |
| LAMP1 | TRPM7 | CD107a\|LAMPA\|LGP120 | Proximity Label-MS | physical | [82] |
| CA9 | TRPM7 | CAIX\|MN | Proximity Label-MS | physical | [83] |
| RHBDD1 | TRPM7 | RRP4 | Proximity Label-MS | physical | [84] |
| FAM105A | TRPM7 | NET20 | Proximity Label-MS | physical | [85] |
| RPL27 | TRPM7 | L27 | Proximity Label-MS | physical | [86] |
| E | TRPM7 | env\|envelope\|SARS-CoV2 E\|E protein\|emp\|SARS-CoV-2 E\|VEMP_SARS2\|PRO_0000449651 | Proximity Label-MS | physical | [87] |
| M | TRPM7 | mem\|membrane\|SARS-CoV2 M\|M protein\|SARS-CoV-2 M\|VME1_SARS2\|PRO_0000449652 | Proximity Label-MS | physical | [87] |
| nsp4 | TRPM7 | ORF1ab\|ORF1ab-nsp4\|SARS-CoV2 nsp4\|SARS-CoV-2 nsp4\|R1AB_SARS2\|PRO_0000449622 | Proximity Label-MS | physical | [87] |
| nsp6 | TRPM7 | ORF1ab\|ORF1ab-nsp6\|SARS-CoV2 nsp6\|SARS-CoV-2 nsp6\|R1AB_SARS2\|PRO_0000449624 | Proximity Label-MS | physical | [87] |
| ORF14 | TRPM7 | SARS-CoV2 ORF14\|SARS-CoV-2 ORF14\|14\|Y14_SARS2\|PRO_0000449658 | Proximity Label-MS | physical | [87] |
| ORF7a | TRPM7 | SARS-CoV2 ORF7a\|SARS-CoV-2 ORF7a\|Protein 7a\|7a\|NS7A_SARS2\|PRO_0000449654 | Proximity Label-MS | physical | [87] |
| ORF7b | TRPM7 | SARS-CoV2 ORF7b\|SARS-CoV-2 ORF7b\|7b\|NS7B_SARS2\|PRO_0000449799 | Proximity Label-MS | physical | [87] |
| S | TRPM7 | spike\|SARS-CoV2 S\|SARS-CoV2 spike\|S protein\|surface\|SARS-CoV-2 spike\|SARS-CoV-2 S\|SPIKE_SARS2\|PRO_0000449646 | Proximity Label-MS | physical | [87] |
| ORF7a | TRPM7 | SARS-CoV2 ORF7a\|SARS-CoV-2 ORF7a\|Protein 7a\|7a\|NS7A_SARS2\|PRO_0000449654 | Proximity Label-MS | physical | [88] |
| E | TRPM7 | env\|envelope\|SARS-CoV2 E\|E protein\|emp\|SARS-CoV-2 E\|VEMP_SARS2\|PRO_0000449651 | Proximity Label-MS | physical | [88] |
| ORF8 | TRPM7 | SARS-CoV2 ORF8\|SARS-CoV-2 ORF8\|8\|NS7B_SARS2\|PRO_0000449655 | Proximity Label-MS | physical | [88] |
| M | TRPM7 | mem\|membrane\|SARS-CoV2 M\|M protein\|SARS-CoV-2 M\|VME1_SARS2\|PRO_0000449652 | Proximity Label-MS | physical | [88] |
| S | TRPM7 | spike\|SARS-CoV2 S\|SARS-CoV2 spike\|S protein\|surface\|SARS-CoV-2 spike\|SARS-CoV-2 S\|SPIKE_SARS2\|PRO_0000449646 | Proximity Label-MS | physical | [88] |
| ORF6 | TRPM7 | SARS-CoV2 ORF6\|SARS-CoV-2 ORF6\|6\|NS6_SARS2\|PRO_0000449653 | Proximity Label-MS | physical | [88] |
| nsp6 | TRPM7 | ORF1ab\|ORF1ab-nsp6\|SARS-CoV2 nsp6\|SARS-CoV-2 nsp6\|R1AB_SARS2\|PRO_0000449624 | Proximity Label-MS | physical | [88] |
| nsp4 | TRPM7 | ORF1ab\|ORF1ab-nsp4\|SARS-CoV2 nsp4\|SARS-CoV-2 nsp4\|R1AB_SARS2\|PRO_0000449622 | Proximity Label-MS | physical | [88] |
| ST7 | TRPM7 | ETS7q\|FAM4A\|FAM4A1\|HELG\|RAY1\|SEN4\|TSG7 | Proximity Label-MS | physical | [89] |
| FKBP8 | TRPM7 | FKBP38\|FKBPr38 | Proximity Label-MS | physical | [90] |
| PTPN1 | TRPM7 | PTP1B | Proximity Label-MS | physical | [90] |
| RHOT2 | TRPM7 | ARHT2\|C16orf39\|MIRO-2\|MIRO2\|RASL | Proximity Label-MS | physical | [90] |
| SLC25A46 | TRPM7 | - | Proximity Label-MS | physical | [90] |
| FASN | TRPM7 | FAS\|OA-519\|SDR27X1 | Positive Genetic | genetic | [74] |
| RPN1 | TRPM7 | OST1\|RBPH1 | Proximity Label-MS | physical | [91] |
| DNAJC16 | TRPM7 | - | Proximity Label-MS | physical | [92] |
| DNAJC1 | TRPM7 | DNAJL1\|ERdj1\|HTJ1\|MTJ1 | Proximity Label-MS | physical | [92] |
| SEC63 | TRPM7 | DNAJC23\|ERdj2\|PRO2507\|SEC63L | Proximity Label-MS | physical | [92] |
| AGO2 | TRPM7 | EIF2C2\|Q10 | Affinity Capture-RNA | physical | [93] |
| ARF6 | TRPM7 | - | Proximity Label-MS | physical | [94] |
| ATP2A1 | TRPM7 | ATP2A\|SERCA1 | Proximity Label-MS | physical | [94] |
| B3GAT1 | TRPM7 | CD57\|GLCATP\|GLCUATP\|HNK1\|LEU7\|NK-1\|NK1 | Proximity Label-MS | physical | [94] |
| BCAP31 | TRPM7 | 6C6-AG\|BAP31\|CDM\|DDCH\|DXS1357E | Proximity Label-MS | physical | [94] |
| CEP135 | TRPM7 | CEP4\|KIAA0635\|MCPH8 | Proximity Label-MS | physical | [94] |
| CKAP4 | TRPM7 | CLIMP-63\|ERGIC-63\|p63 | Proximity Label-MS | physical | [94] |
| CYP2C9 | TRPM7 | CPC9\|CYP2C\|CYP2C10\|CYPIIC9\|P450IIC9 | Proximity Label-MS | physical | [94] |
| DHFRL1 | TRPM7 | DHFRP4 | Proximity Label-MS | physical | [94] |
| DIRAS3 | TRPM7 | ARHI\|NOEY2 | Proximity Label-MS | physical | [94] |
| ELOVL5 | TRPM7 | HELO1\|SCA38\|dJ483K16.1 | Proximity Label-MS | physical | [94] |
| EMD | TRPM7 | EDMD\|LEMD5\|STA | Proximity Label-MS | physical | [94] |
| ERGIC1 | TRPM7 | ERGIC-32\|ERGIC32\|NET24 | Proximity Label-MS | physical | [94] |
| ERGIC2 | TRPM7 | Erv41\|PTX1\|cd002 | Proximity Label-MS | physical | [94] |
| GJA1 | TRPM7 | AVSD3\|CMDR\|CX43\|GJAL\|HLHS1\|HSS\|ODDD | Proximity Label-MS | physical | [94] |
| GJD3 | TRPM7 | CX31.9\|Cx30.2\|GJA11\|GJC1 | Proximity Label-MS | physical | [94] |
| HSD17B11 | TRPM7 | 17-BETA-HSD11\|17-BETA-HSDXI\|17BHSD11\|DHRS8\|PAN1B\|RETSDR2\|SDR16C2 | Proximity Label-MS | physical | [94] |
| HSD3B7 | TRPM7 | CBAS1\|PFIC4\|SDR11E3 | Proximity Label-MS | physical | [94] |
| KRT18 | TRPM7 | CYK18\|K18 | Proximity Label-MS | physical | [94] |
| KRT19 | TRPM7 | CK19\|K19\|K1CS | Proximity Label-MS | physical | [94] |
| LAMP1 | TRPM7 | CD107a\|LAMPA\|LGP120 | Proximity Label-MS | physical | [94] |
| LAMP2 | TRPM7 | CD107b\|LAMP-2\|LAMPB\|LGP110 | Proximity Label-MS | physical | [94] |
| LAMP3 | TRPM7 | CD208\|DC LAMP\|DC-LAMP\|DCLAMP\|LAMP\|LAMP-3\|TSC403 | Proximity Label-MS | physical | [94] |
| LMAN1 | TRPM7 | ERGIC-53\|ERGIC53\|F5F8D\|FMFD1\|MCFD1\|MR60\|gp58 | Proximity Label-MS | physical | [94] |
| KIAA1715 | TRPM7 | LNP\|LNP1\|Ul\|ulnaless | Proximity Label-MS | physical | [94] |
| LRRC59 | TRPM7 | p34 | Proximity Label-MS | physical | [94] |
| METTL7A | TRPM7 | AAM-B | Proximity Label-MS | physical | [94] |
| NDC80 | TRPM7 | HEC\|HEC1\|HsHec1\|KNTC2\|TID3\|hsNDC80 | Proximity Label-MS | physical | [94] |
| NUP155 | TRPM7 | ATFB15\|N155 | Proximity Label-MS | physical | [94] |
| OCLN | TRPM7 | BLCPMG\|PPP1R115 | Proximity Label-MS | physical | [94] |
| PANX1 | TRPM7 | MRS1\|PX1\|UNQ2529 | Proximity Label-MS | physical | [94] |
| PXMP2 | TRPM7 | PMP22 | Proximity Label-MS | physical | [94] |
| RAB2A | TRPM7 | LHX\|RAB2 | Proximity Label-MS | physical | [94] |
| RAB35 | TRPM7 | H-ray\|RAB1C\|RAY | Proximity Label-MS | physical | [94] |
| RAB5C | TRPM7 | L1880\|RAB5CL\|RAB5L\|RABL | Proximity Label-MS | physical | [94] |
| RAB9A | TRPM7 | RAB9 | Proximity Label-MS | physical | [94] |
| RHOB | TRPM7 | ARH6\|ARHB\|MST081\|MSTP081\|RHOH6 | Proximity Label-MS | physical | [94] |
| RPN1 | TRPM7 | OST1\|RBPH1 | Proximity Label-MS | physical | [94] |
| RPN2 | TRPM7 | RIBIIR\|RPN-II\|RPNII\|SWP1 | Proximity Label-MS | physical | [94] |
| SEC61B | TRPM7 | - | Proximity Label-MS | physical | [94] |
| SEC62 | TRPM7 | Dtrp1\|HTP1\|TLOC1\|TP-1 | Proximity Label-MS | physical | [94] |
| SSR1 | TRPM7 | TRAPA | Proximity Label-MS | physical | [94] |
| STX7 | TRPM7 | - | Proximity Label-MS | physical | [94] |
| SYNE3 | TRPM7 | C14orf49\|NET53\|Nesp3 | Proximity Label-MS | physical | [94] |
| PNLDC1 | TRPM7 | - | Affinity Capture-MS | physical | [95] |
| ZACN | TRPM7 | L2\|LGICZ\|LGICZ1\|ZAC\|ZAC1 | Affinity Capture-MS | physical | [95] |
| RABEP2 | TRPM7 | FRA | Affinity Capture-MS | physical | [95] |
| RYK | TRPM7 | D3S3195\|JTK5\|JTK5A\|RYK1 | Affinity Capture-MS | physical | [95] |
| TEX28 | TRPM7 | CXorf2\|MRX99\|TEX28P1\|TEX28P2\|fTEX | Affinity Capture-MS | physical | [95] |
| NKAIN1 | TRPM7 | FAM77C | Affinity Capture-MS | physical | [95] |
| SNAP29 | TRPM7 | CEDNIK\|SNAP-29 | Affinity Capture-MS | physical | [95] |
| CD3D | TRPM7 | CD3-DELTA\|IMD19\|T3D | Affinity Capture-MS | physical | [95] |
| PSCA | TRPM7 | PRO232 | Affinity Capture-MS | physical | [95] |
| PTP4A1 | TRPM7 | HH72\|PRL-1\|PRL1\|PTP(CAAX1)\|PTPCAAX1 | Affinity Capture-MS | physical | [95] |
| PKD2L2 | TRPM7 | TRPP5 | Affinity Capture-MS | physical | [95] |
| TTYH1 | TRPM7 | - | Affinity Capture-MS | physical | [95] |
| EIF2B5 | TRPM7 | CACH\|CLE\|EIF-2B\|EIF2Bepsilon\|LVWM | Affinity Capture-MS | physical | [95] |
| ARL15 | TRPM7 | ARFRP2 | Affinity Capture-MS | physical | [95] |
| C3orf52 | TRPM7 | TTMP | Affinity Capture-MS | physical | [95] |
| CCDC107 | TRPM7 | PSEC0222 | Affinity Capture-MS | physical | [95] |
| ANPEP | TRPM7 | APN\|CD13\|GP150\|LAP1\|P150\|PEPN | Proximity Label-MS | physical | [96] |
| TMPRSS11B | TRPM7 | - | Proximity Label-MS | physical | [96] |
| CLEC4D | TRPM7 | CLEC-6\|CLEC6\|CLECSF8\|MCL\|MPCL | Proximity Label-MS | physical | [96] |
| CLEC4E | TRPM7 | CLECSF9\|MINCLE | Proximity Label-MS | physical | [96] |
| RHOG | TRPM7 | ARHG | Affinity Capture-MS | physical | [97] |
| RHOA | TRPM7 | ARH12\|ARHA\|RHO12\|RHOH12 | Affinity Capture-MS | physical | [97] |
| CDC42 | TRPM7 | CDC42Hs\|G25K | Affinity Capture-MS | physical | [97] |
| RHOQ | TRPM7 | ARHQ\|HEL-S-42\|RASL7A\|TC10\|TC10A | Affinity Capture-MS | physical | [97] |
| RHOB | TRPM7 | ARH6\|ARHB\|MST081\|MSTP081\|RHOH6 | Affinity Capture-MS | physical | [97] |
| RHOD | TRPM7 | ARHD\|RHOHP1\|RHOM\|Rho | Affinity Capture-MS | physical | [97] |
| RHOF | TRPM7 | ARHF\|RIF | Affinity Capture-MS | physical | [97] |
| RHOU | TRPM7 | ARHU\|CDC42L1\|G28K\|WRCH1\|hG28K | Affinity Capture-MS | physical | [97] |
| RHOH | TRPM7 | ARHH\|TTF | Affinity Capture-MS | physical | [97] |
| RND1 | TRPM7 | ARHS\|RHO6\|RHOS | Affinity Capture-MS | physical | [97] |
| RND2 | TRPM7 | ARHN\|RHO7\|RhoN | Affinity Capture-MS | physical | [97] |
| RND3 | TRPM7 | ARHE\|Rho8\|RhoE\|memB | Affinity Capture-MS | physical | [97] |
| RHOJ | TRPM7 | ARHJ\|RASL7B\|TC10B\|TCL | Affinity Capture-MS | physical | [97] |
| RAC1 | TRPM7 | Rac-1\|TC-25\|p21-Rac1 | Affinity Capture-MS | physical | [97] |
| RAC2 | TRPM7 | EN-7\|Gx\|HSPC022\|p21-Rac2 | Affinity Capture-MS | physical | [97] |
| RAC3 | TRPM7 | - | Affinity Capture-MS | physical | [97] |
| RHOC | TRPM7 | ARH9\|ARHC\|H9\|RHOH9 | Affinity Capture-MS | physical | [97] |
| RHOV | TRPM7 | ARHV\|CHP\|WRCH2 | Affinity Capture-MS | physical | [97] |
| EGFR | TRPM7 | ERBB\|ERBB1\|HER1\|NISBD2\|PIG61\|mENA | Negative Genetic | genetic | [72] |
| KRAS | TRPM7 | C-K-RAS\|CFC2\|K-RAS2A\|K-RAS2B\|K-RAS4A\|K-RAS4B\|KI-RAS\|KRAS1\|KRAS2\|NS\|NS3\|RASK2 | Negative Genetic | genetic | [72] |
| RIT1 | TRPM7 | NS8\|RIBB\|RIT\|ROC1 | Negative Genetic | genetic | [72] |
| RAB7A | TRPM7 | PRO2706\|RAB7 | Proximity Label-MS | physical | [98] |
| ARL15 | TRPM7 | ARFRP2 | Proximity Label-MS | physical | [99] |
| INSR | TRPM7 | CD220\|HHF5 | Proximity Label-MS | physical | [100] |
| SFN | TRPM7 | YWHAS | Proximity Label-MS | physical | [101] |
| YWHAB | TRPM7 | GW128\|HEL-S-1\|HS1\|KCIP-1\|YWHAA | Proximity Label-MS | physical | [101] |
| YWHAE | TRPM7 | 14-3-3E\|HEL2\|KCIP-1\|MDCR\|MDS | Proximity Label-MS | physical | [101] |
| YWHAG | TRPM7 | 14-3-3GAMMA\|PPP1R170 | Proximity Label-MS | physical | [101] |
| YWHAH | TRPM7 | YWHA1 | Proximity Label-MS | physical | [101] |
| YWHAQ | TRPM7 | 14-3-3\|1C5\|HS1 | Proximity Label-MS | physical | [101] |
| YWHAZ | TRPM7 | 14-3-3-zeta\|HEL-S-3\|HEL4\|KCIP-1\|YWHAD | Proximity Label-MS | physical | [101] |
| Rab18 | TRPM7 | AA959686 | Proximity Label-MS | physical | [102] |
| FBXW7 | TRPM7 | AGO\|CDC4\|FBW6\|FBW7\|FBX30\|FBXO30\|FBXW6\|SEL-10\|SEL10\|hAgo\|hCdc4 | Negative Genetic | genetic | [73] |
| ESYT1 | TRPM7 | FAM62A\|MBC2 | Proximity Label-MS | physical | [103] |
| ITSN1 | TRPM7 | ITSN\|SH3D1A\|SH3P17 | Two-hybrid | physical | [70] |
| MCAM | TRPM7 | CD146\|MUC18 | Proximity Label-MS | physical | [104] |

The interaction network was generated using STRING tool [105]. Two major subnetworks were identified and illustrated in the Figure 1: 87 genes were mostly associated with Small GTPase mediated signal transduction and 36 genes were described as involved in protein processing in endoplasmic reticulum.


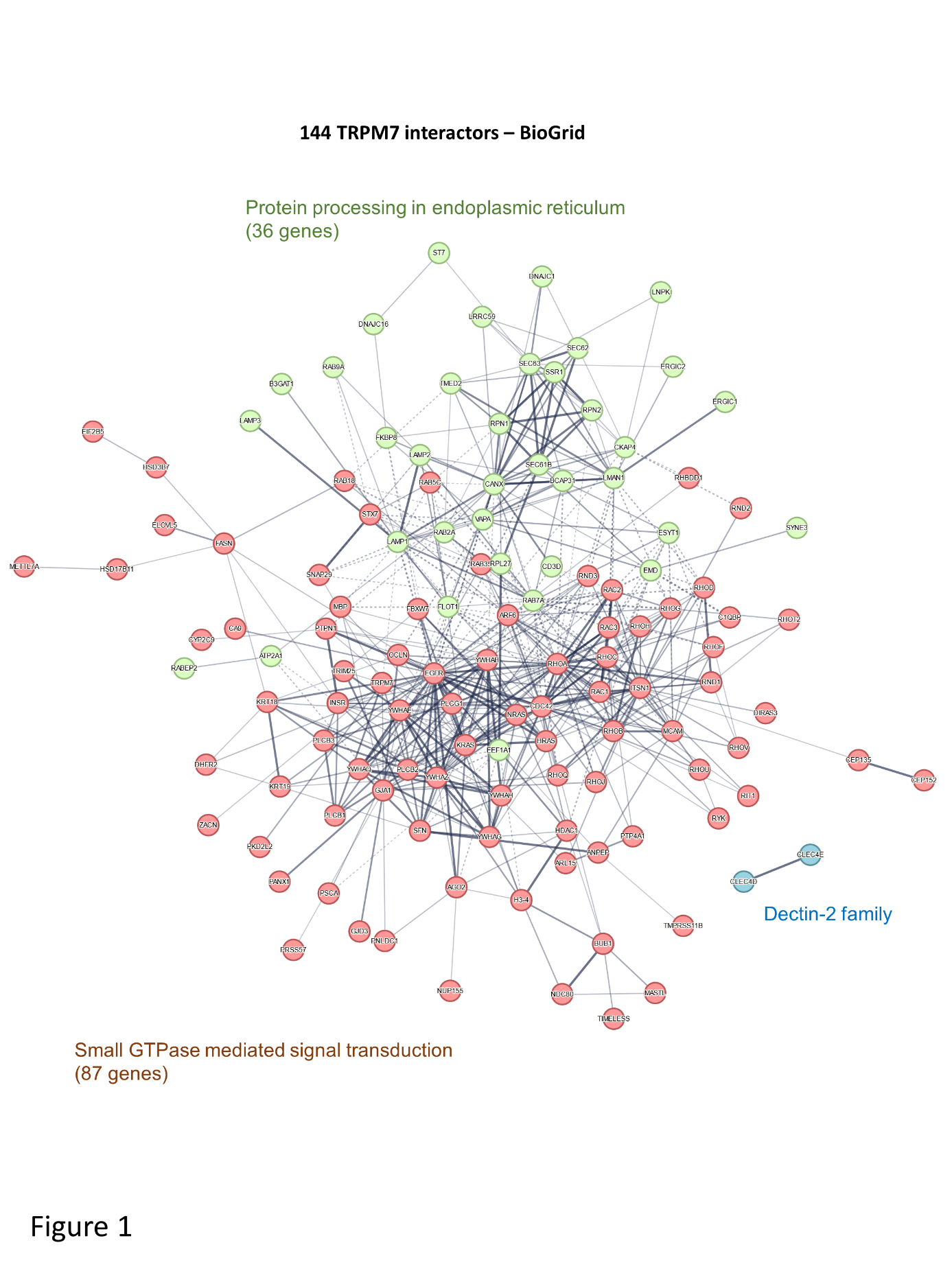


**Figure 1:** STRING protein-protein network of TRPM7 interactors from BioGRID database. 144 TRPM7 interactors were retrieved from BioGRID database. STRING network was realized using https://string-db.org/. Three k-means clusters were separated based on their centroids. Description of the overall ontology of clusters is indicated.

We performed a gene-set enrichment analysis (GSEA) using ShinyGO tool [106]. Kyoto Encyclopedia of Genes and Genomes (KEGG) terms corresponding to the VEGF signaling pathway (hsa04370), Adherens junction (hsa04520), gap junction (hsa04540), sphingolipid signaling pathway (hsa04071), and phospholipase D signaling pathway (hsa04072) were significantly enriched (Figure 2). The biological processes associated with cell morphology (GO:0030865 cortical cytoskeleton organization, GO:0008360 reg. of cell shape, GO:0022604 reg. of cell morphogenesis), cell signaling (GO:0007264 small GTPase mediated signal transduction, GO:0007265 Ras protein signal transduction) or protein trafficking (GO:0006886 intracellular protein transport, GO:0034613 cellular protein localization) were significantly highlighted. VEGF signaling pathway and adherens/gap junction terms were significantly associated with GTPase such as RAC1/2/3, RHOA, CDC42 or HRAS/KRAS/NRAS (Figure 2).


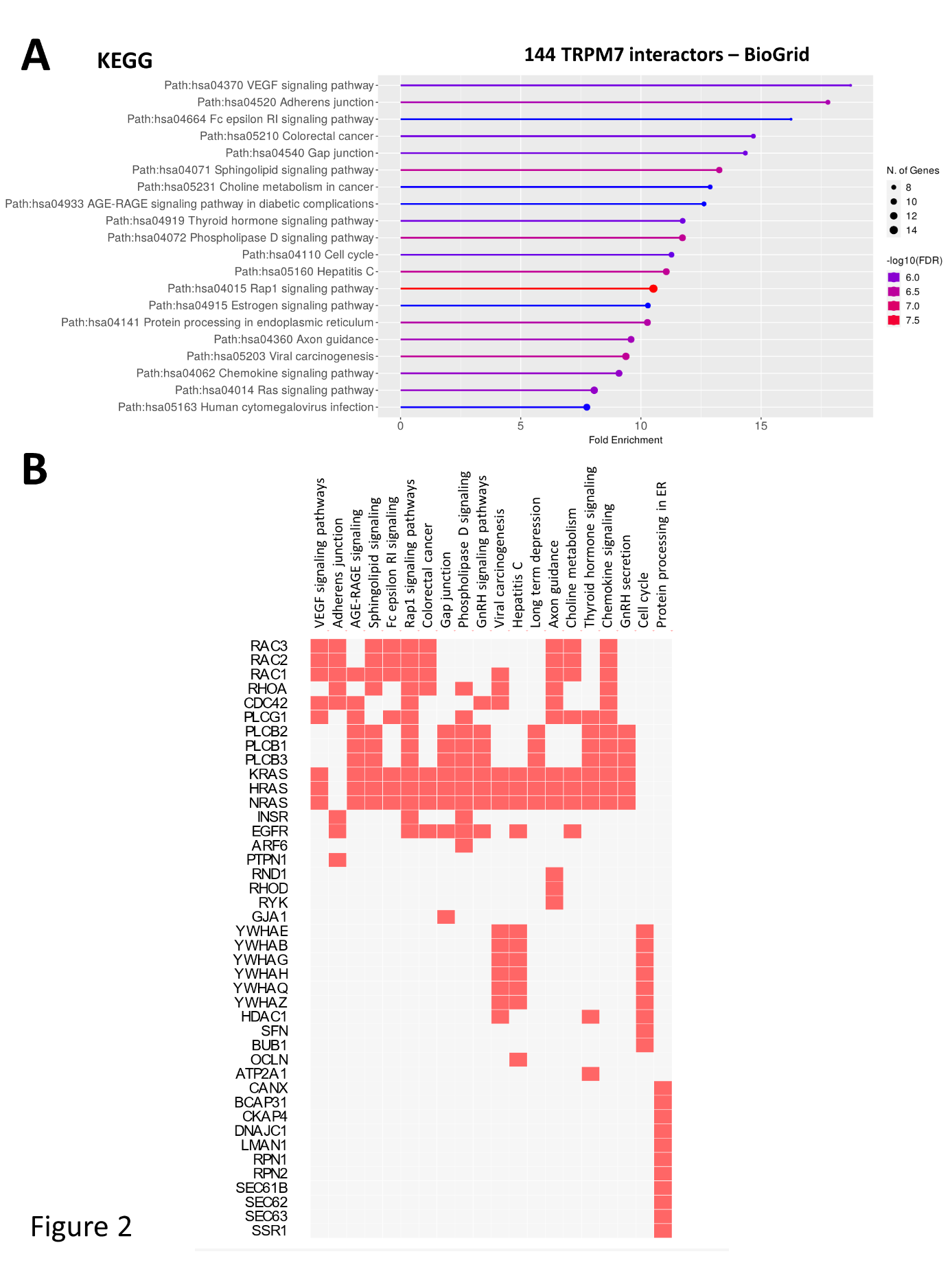


**Figure 2:** Ontology of TRPM7 interactors from BioGRID. (**A**) Dot plot of Gene-set enrichment analysis using ShinyGO v0.81 tool. Dot points are sized by the proportion of all proteins annotated with the KEGG corresponding term and coloured by enrichment confidence (FDR). (**B**) Clustergram of 144 TRPM7 interactors from BioGRID was realized using Enrichr tool (https://maayanlab.cloud/Enrichr/enrich). Enriched Terms are displayed as columns and input genes as rows. Genes associated with a term are indicated as red boxes in the matrix.

b. Predicted TRPM7 interactome

The protein interactome was predicted using ProteinPrompt that is based on machine learning using the sequences of protein pairs as input to estimate their tendency to bind [107]. We selected a threshold of 0.75 as it corresponds with the lowest false positive rate. Q96QT4 amino acid sequence which is the canonical sequence of TRPM7 was used as input. We identified 906 proteins that are predicted to interact with TRPM7 (Table S1). Interestingly, TRPM7 is predicted to form a homomeric protein (ProteinPrompt score = 1.0). Among the best prediction, TRPM6 is a genuine partner (ProteinPrompt score = 0.9360). Most of the known interactors described in this manuscript showed fair ProteinPrompt scores with the exception of PACT network formed by PRL1/PTP4A1, CNNMs and ARL15 proteins (Table 2). Additionally, among the list of TRPM7 interaction partners identified by Bai *et al.* that are not enlisted in BioGRID database, only IRAK1 (ProteinPrompt score = 0.77) belongs to the predicted list of TRPM7 interactors, suggesting a potential mechanistic link between TRPM7 and Toll-like receptor (TLR)/Interleukin-1 receptor (IL-1R) signaling pathway in cancer cells. Moreover, IRAK1 activation is frequently associated with progression and therapeutic resistance of cancers [108].

Syndecan-2 (SDC2), activating signal cointegrator 1 (TRIP4), lamin-B1, AT-rich interactive domain-containing protein 2 (ARID2) did not reach the 0.75 cut off but still displayed fair interaction score (0.6147-0.7027), suggesting either a weak physical interaction or the need of cofactors to stabilize the protein complexes. Direct proof of physical interaction such as co-immunoprecipitation remains necessary to validate these TRPM7 interactors. Alternatively, instead of forming a tightly bound complex, TRPM7 and partners with mild/low interaction scores may be localized in the vicinity to regulate TRPM7 biological function.

**Table 2:** List of TRPM7 known interactors and interaction prediction using ProteinPrompt.

| Protein | Interation_score (interaction = 1) | Uniprot ID | Full description |
| --- | --- | --- | --- |
| AKT1 | 0.8787 | P31749 | RAC-alpha serine/threonine-protein kinase |
| AKT2 | 0.7280 | P31751 | RAC-beta serine/threonine-protein kinase |
| AKT3 | 0.6200 | Q9Y243 | RAC-gamma serine/threonine-protein kinase |
| Q5TZZ9 | 0.6720 | Q5TZZ9 | ANXA1 {ECO:0000313\|EMBL:ADZ76495.1} |
| E7ETZ0 | 0.7293 | E7ETZ0 | CALM1 {ECO:0000313\|Ensembl:ENSP00000403491} |
| S10A1 | 0.7960 | P23297 | Protein S100-A1 |
| CNNM1 | 0.4360 | Q9NRU3 | Metal transporter CNNM1 |
| CNNM2 | 0.4000 | Q9H8M5 | Metal transporter CNNM2 |
| CNNM3 | 0.4133 | Q8NE01 | Metal transporter CNNM3 |
| CNNM4 | 0.5080 | Q6P4Q7 | Metal transporter CNNM4 |
| TP4A1 | 0.4440 | Q93096 | Protein tyrosine phosphatase type IVA 1 precursor |
| ARL15 | 0.5200 | Q9NXU5 | ADP-ribosylation factor-like protein 15 |
| Q5U0J5 | 0.7720 | Q5U0J5 | CREB1 {ECO:0000313\|EMBL:EAW70405.1} |
| EF2 | 0.9907 | P13639 | Elongation factor 2 |
| EGFR | 0.8173 | P00533 | Epidermal growth factor receptor precursor |
| H31T | 0.9733 | Q16695 | Histone H3.1t |
| H31 | 0.9520 | P68431 | Histone H3.1 |
| H32 | 0.9093 | Q71DI3 | Histone H3.2 |
| H33 | 0.9013 | P84243 | Histone H3.3 |
| B2R4P9 | 0.9013 | B2R4P9 | Histone H3 |
| MBP | 0.7893 | P02686 | Myelin basic protein |
| MYH1 | 0.6373 | P12882 | Myosin-1 |
| MYH2 | 0.6907 | Q9UKX2 | Myosin-2 |
| MYH3 | 0.5400 | P11055 | Myosin-3 |
| MYO3A | 0.5880 | Q8NEV4 | Myosin-IIIa |
| MYO3B | 0.5440 | Q8WXR4 | Myosin-IIIb |
| MYH4 | 0.5787 | Q9Y623 | Myosin-4 |
| Q7Z7A5 | 0.6387 | Q7Z7A5 | Myosin 5B |
| MYH6 | 0.5387 | P13533 | Myosin-6 |
| MYH7 | 0.5680 | P12883 | Myosin-7 |
| MYH7B | 0.3987 | A7E2Y1 | Myosin-7B |
| MYH8 | 0.6120 | P13535 | Myosin-8 |
| MYH9 | 0.7600 | P35579 | Myosin-9 |
| MYH10 | 0.7560 | P35580 | Myosin-10 |
| MYH11 | 0.6107 | P35749 | Myosin-11 |
| MYH13 | 0.5720 | Q9UKX3 | Myosin-13 |
| MYH14 | 0.5107 | Q7Z406 | Myosin-14 |
| MYH15 | 0.4693 | Q9Y2K3 | Myosin-15 |
| PAK1 | 0.7480 | Q13153 | Serine/threonine-protein kinase PAK 1 |
| PLCB2 | 0.9333 | Q00722 | 1-phosphatidylinositol 4,5-bisphosphate phosphodiesterase beta-2 |
| PLCG1 | 0.9280 | P19174 | 1-phosphatidylinositol 4,5-bisphosphate phosphodiesterase gamma-1 |
| PLCG2 | 0.7427 | P16885 | 1-phosphatidylinositol 4,5-bisphosphate phosphodiesterase gamma-2 |
| PLCB3 | 0.7307 | Q01970 | 1-phosphatidylinositol 4,5-bisphosphate phosphodiesterase beta-3 |
| PLCB1 | 0.6933 | Q9NQ66 | 1-phosphatidylinositol 4,5-bisphosphate phosphodiesterase beta-1 |
| PLCH2 | 0.5960 | O75038 | 1-phosphatidylinositol 4,5-bisphosphate phosphodiesterase eta-2 |
| PLCB4 | 0.5760 | Q15147 | 1-phosphatidylinositol 4,5-bisphosphate phosphodiesterase beta-4 |
| RHOA | 0.8747 | P61586 | Transforming protein RhoA precursor |
| SMAD2 | 0.8747 | Q15796 | Mothers against decapentaplegic homolog 2 |
| SNAPN | 0.7760 | O95295 | SNARE-associated protein Snapin |
| SYN1 | 0.7333 | P17600 | Synapsin-1 |
| SYT1 | 0.6773 | P21579 | Synaptotagmin-1 |

By performing similar GSEA using ShinyGO tool, we observed a significant enrichment of genes associated with pancreatic cancer (hsa05212), microRNAs in cancer (hsa05206), cell cycle (hsa04110), breast cancer (hsa05224), proteoglycans in cancer (hsa05205), chemokine signaling pathway (hsa04062), pathways in cancer (hsa05200), focal adhesion (hsa04510) and different signaling pathways (hsa04015 Rap1, hsa04010 MAPK, hsa04014 Ras and hsa04151 PI3K-Akt (Figure 3). These enrichments are mostly related with genes belonging to the RAC, PLC and RAS families.


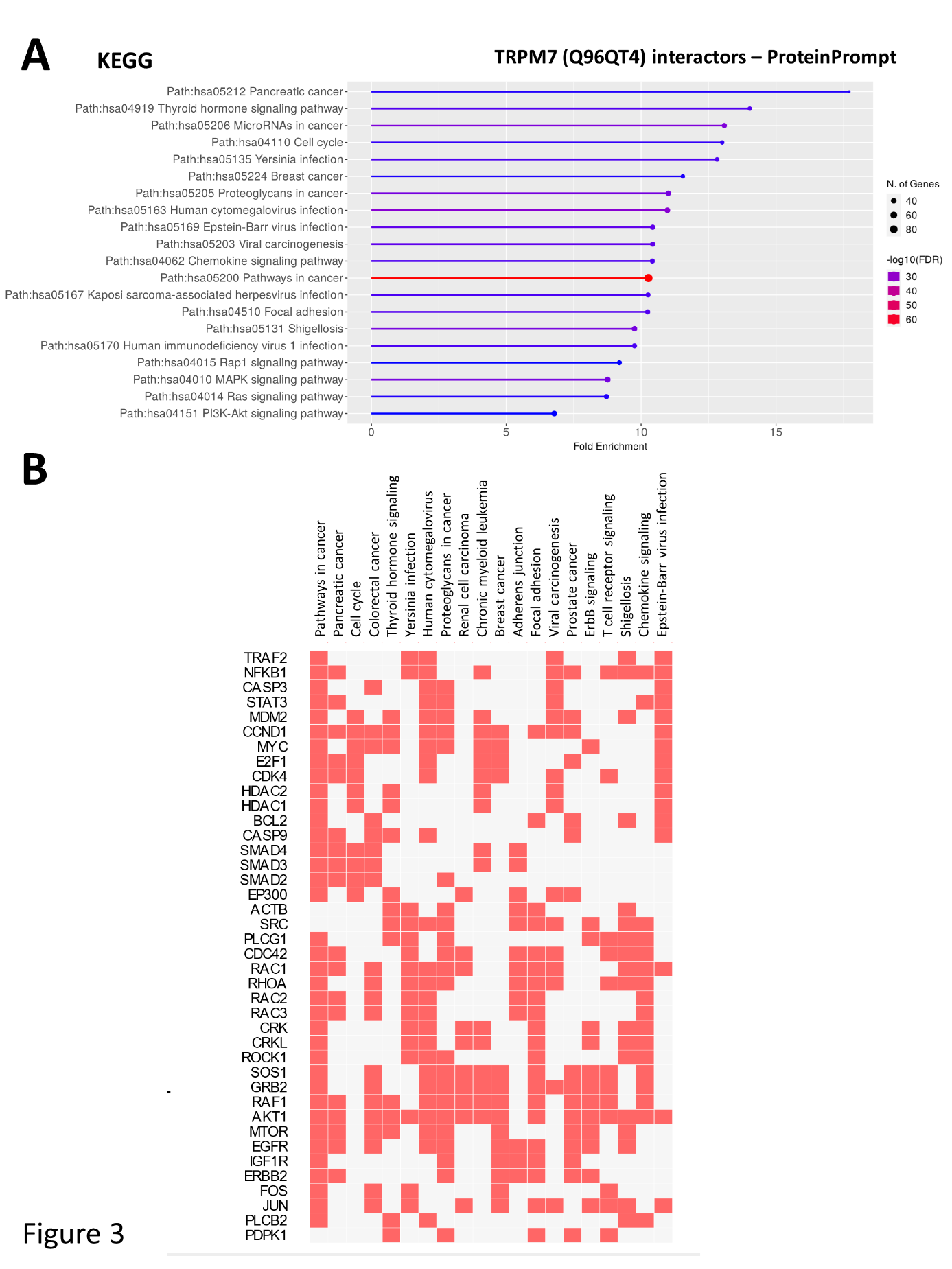


**Figure 3:** Ontology of the TRPM7 predicted interactome. (**A**) The protein interactome was determined using Q96Q4T amino acid sequence (canonical sequence of TRPM7) as input. Gene-set enrichment analyses were performed using ShinyGO v0.81 tool. (**B**) Clustergram of TRPM7 predicted interactors from ProteinPrompt was realized using Enrichr tool (https://maayanlab.cloud/Enrichr/enrich). Enriched Terms are displayed as columns and input genes as rows. The genes associated with a term are indicated as red boxes in the matrix.

The mRNA expression information can be combined with protein-protein interaction network analysis in order to identify *bona fide* partner. Indeed, physical interactions require co-expression of both proteins in the same cell or in the surrounding cells (for paracrine interaction). To this aim, we retrieved quantification data from RNAseq (RSEM) for TRPM7 using cbioportal in every TCGA datasets available and identified a list of genes that are correlated with TRPM7 (Spearman correlation, corrected q value <0.05) (Table S2). We observed approximatively 100 potential interactors in most of the TCGA datasets. Interestingly, we observed 51 common predicted and correlated interactors in digestive cancers, including esophageal, gastric, pancreatic and colorectal cancers (ESCA, STAD, PAAD and COADREAD) (Figure 4A). GSEA using ShinyGO tool showed significant enrichments of KEGG pathways such as cell cycle (hsa04110), TGFβ, p53, thyroid hormone, FoxO, estrogen signaling pathways (hsa04350, hsa04115, has04919, hsa04068, 04915, hsa05200), cellular senescence (hsa04218) (Figure 4B).


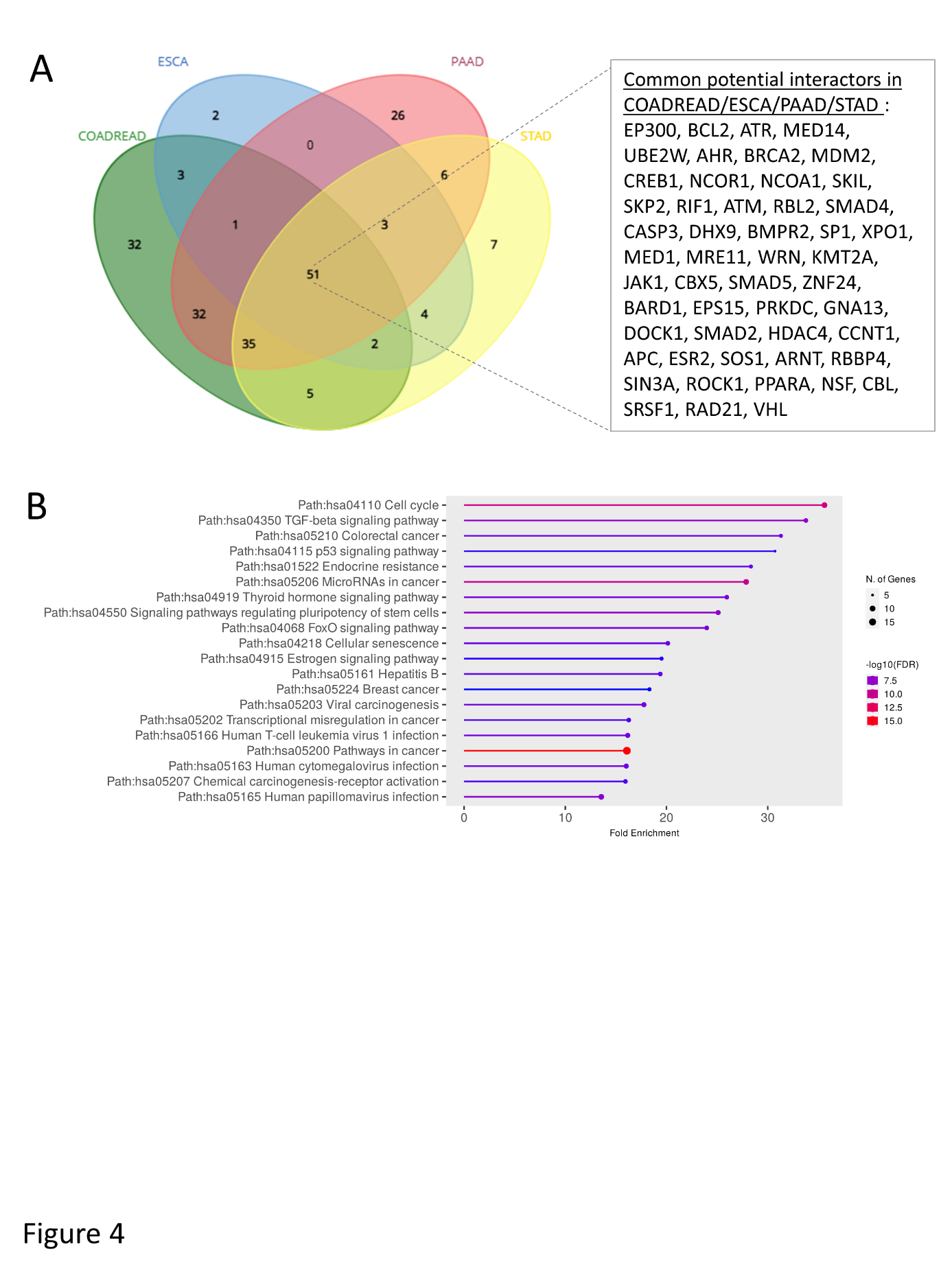


**Figure 4:** Venn diagram of *bona fide* TRPM7 interactors that are co-expressed in TCGA datasets and ontology analysis. (**A**) Venn diagram of coexpressed/predicted TRPM7 interactors in esophageal, gastric, colorectal and pancreatic cancers (ESCA, STAD, COADREAD and PAAD). Common genes are enlisted on the right panel. (**B**) Dot plot of Gene-set enrichment analysis using ShinyGO v0.81 tool.

c. Predicted interactors that also have experimental evidence: Could these be new candidates for TRPM7 interaction?

We observed 19 predicted interactors that also harbored experimental evidences (illustrated in figure 5A). Interestingly, we found 7 different GTPases and 2 phospholipase C proteins. We also highlighted the interaction between TRPM7 and two tyrosine kinase receptors (EGFR and INSR). Their downstream signaling pathways play major roles in cancer by regulating biological processes such as proliferation, survival, differentiation, and metastasis. Altogether this reinforces the mechanistic link between TRPM7 and cell signaling. Finally, TRPM7 also interacts with occludin that is a component of cell junction. Its alteration is commonly associated with increased epithelial–mesenchymal transition (EMT) and tumor progression. Surprisingly, TRPM7 was shown to interact with nuclear proteins HDAC1 and AGO2. In their previous work, Krapinvinsky *et al.* have already identified nuclear proteins as interactors of TRPM7 cleaved kinase fragments (M7CKs) [50]. HDAC1 may interact with M7CKs as we observed a fair interaction ProteinPrompt score (0.6067) with restricted amino acid sequence corresponding to the alpha kinase domain (AA1594-1824). We hypothesize that TRPM7 could act as a connector between signaling pathways and gene silencing machinery. This remains to be fully deciphered in future investigations.


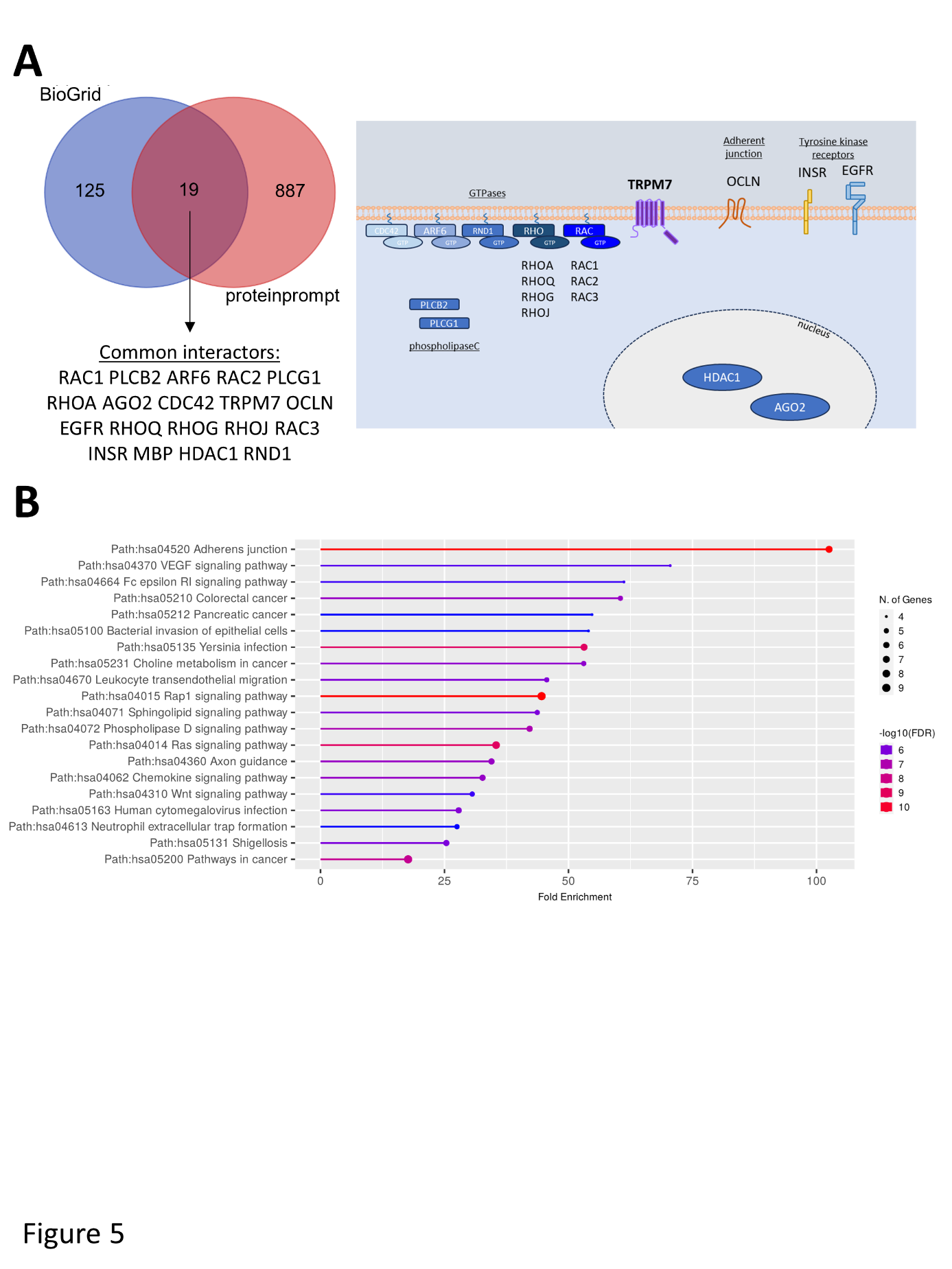


**Figure 5:** Venn diagram of *bona fide* TRPM7 interactors and ontology analysis. (**A**) Venn diagram of 144 TRPM7 interactors with experimental evidence (from BioGRID database) and 906 predicted interactors (from ProteinPrompt analysis) showed 19 common genes illustrated on the right panel. (**B**) Dot plot of Gene-set enrichment analysis using ShinyGO v0.81 tool.

**4. Perspectives in drug discovery:**

TRPM7 has been implicated in numerous diseases, including cancer. Its dual function as a cation channel and kinase, as well as its ubiquitous nature, complicate its multiple interactions with proteins involved in signal transduction and the regulation of cellular properties. Targeting the TRPM7 protein appears difficult given its essential role in regulating intracellular magnesium homeostasis and the current lack of specific pharmacological blockers. However, the anticancer agent CCT128930 has been recently identified as a potent inhibitor of TRPM7 channels [21, 109]. Many TRPM7 interactors in the BioGRID database were initially found in large scale mass spectrometry experiments and still require specific evidences. Moreover, ProteinPrompt prediction analysis may help to prioritize subset of novel interactors for experimental validation. Therefore, a better understanding of TRPM7's interaction partners and the underlying intracellular signaling mechanisms would enable the development of new therapeutic strategies based on the destabilization of these protein complexes or by use of bispecific antibodies allowing the direct targeting of particular cell types. Several GTPase-modulating drugs have been described recently. Notably KRAS drugs have been developed. Sotorasib is a potent and highly selective inhibitor of KRAS^G12C^ and is used in clinical trials for lung cancer harboring this mutation. Several works also focused on strategy of blocking interaction between KRAS and its partners. Deltarasin is blocking KRAS–PDEδ interaction and alter location of KRAS [110]). Finally, BI-2852 blocks the interaction KRAS with GEF effector [111]. Combination of such small GTPase inhibitors with TRPM7 blockers may be proposed as a promising therapeutical strategy to target the oncoprotein complexes involving TRPM7 in cancer cells.

Moreover, protein-protein interaction inhibitors disrupt critical molecular interfaces that regulate oncogenic signaling and associated cancer cell aggressiveness [112]. In order to develop such drugs (chemical compound/peptide) targeting TRPM7 interactions, further biochemical evidences, notably knowledge of the protein structure of both partner at the cell membrane as well as the interaction surface need to be obtained.

**5. Concluding remarks:**

In this work, we have compiled an inventory of the numerous interaction partners of TRPM7, revealing the diversity and complexity of the mechanisms involving this protein. The use of computational and modeling tools has enabled us to identify new potential partners, opening up new therapeutic prospects for the development of drugs targeting these intracellular signaling protein complexes.

**Acknowledgements:**

Correlation analyses are based upon data extracted from the TCGA Research Network (<http://cancergenome.nih.gov/>). We thank Pr. Staritzbichler and Pr. Hildebrand for development of the ProteinPrompt webserver that was used for protein interaction prediction.

**Funding:**

This work has been founded by The French National Cancer Institute (INCA) (PLBIO2024 grant INCa_19414), by the Cancéropôle Nord-Ouest (CNO), by the Ligue Contre le Cancer (Septentrion and Somme’s committees), and by the MOSOPS project. The MOSOPS project has received financial support from the French State, Hauts-de-France region, INSERM and A2U Alliance’s Universities. This work was also supported by grants from Contrat de Plan Etat-Région CPER Cancer 2015-2020 and Oncolille Institute.

**Conflict of interest:**

The authors declare no conflict of interest.

**Figure legends and Tables:**

Figure 1: STRING protein-protein network of TRPM7 interactors from BioGRID database. 144 TRPM7 interactors were retrieved from BioGRID database. STRING network was realized using https://string-db.org/. Three k-means clusters were separated based on their centroids. Description of the overall ontology of clusters is indicated.

Figure 2: Ontology of TRPM7 interactors from BioGRID. (**A**) Dot plot of Gene-set enrichment analysis using ShinyGO v0.81 tool. Dot points are sized by the proportion of all proteins annotated with the KEGG corresponding term and coloured by enrichment confidence (FDR). (**B**) Clustergram of 144 TRPM7 interactors from BioGRID was realized using Enrichr tool (https://maayanlab.cloud/Enrichr/enrich). Enriched Terms are displayed as columns and input genes as rows. Genes associated with a term are indicated as red boxes in the matrix.

Figure 3: Ontology of the TRPM7 predicted interactome. (**A**) The protein interactome was determined using Q96Q4T amino acid sequence (canonical sequence of TRPM7) as input. Gene-set enrichment analyses were performed using ShinyGO v0.81 tool. (**B**) Clustergram of TRPM7 predicted interactors from ProteinPrompt was realized using Enrichr tool (https://maayanlab.cloud/Enrichr/enrich). Enriched Terms are displayed as columns and input genes as rows. The genes associated with a term are indicated as red boxes in the matrix.

Figure 4: Venn diagram of bona fide TRPM7 interactors that are co-expressed in TCGA datasets and ontology analysis. (A) Venn diagram of coexpressed/predicted TRPM7 interactors in esophageal, gastric, colorectal and pancreatic cancers (ESCA, STAD, COADREAD and PAAD). Common genes are enlisted on the right panel. (B) Dot plot of Gene-set enrichment analysis using ShinyGO v0.81 tool.

Figure 5: Venn diagram of *bona fide* TRPM7 interactors and ontology analysis. (A) Venn diagram of 144 TRPM7 interactors with experimental evidence (from BioGRID database) and 906 predicted interactors (from ProteinPrompt analysis) showed 19 common genes illustrated on the right panel. (B) Dot plot of Gene-set enrichment analysis using ShinyGO v0.81 tool.

Table 1: List of TRPM7 interactors enlisted in the BioGRID database.

Table 2: List of TRPM7 known interactors and interaction prediction using ProteinPrompt.

Table S1: List of TRPM7 interactors predicted using ProteinPrompt.

Table S2: List of common genes predicted as TRPM7 interactors using proteinprompt and correlated with TRPM7 in TCGA datasets.

**Reference:**

1 Nadler MJ, Hermosura MC, Inabe K, Perraud AL, Zhu Q, Stokes AJ, Kurosaki T, Kinet JP, Penner R, Scharenberg AM, Fleig A: LTRPC7 is a Mg.ATP-regulated divalent cation channel required for cell viability. Nature 2001;411:590-595.

2 Runnels LW, Yue L, Clapham DE: TRP-PLIK, a bifunctional protein with kinase and ion channel activities. Science 2001;291:1043-1047.

3 Ryazanova LV, Dorovkov MV, Ansari A, Ryazanov AG: Characterization of the protein kinase activity of TRPM7/ChaK1, a protein kinase fused to the transient receptor potential ion channel. J Biol Chem 2004;279:3708-3716.

4 Chubanov V, Waldegger S, Mederos y Schnitzler M, Vitzthum H, Sassen MC, Seyberth HW, Konrad M, Gudermann T: Disruption of TRPM6/TRPM7 complex formation by a mutation in the TRPM6 gene causes hypomagnesemia with secondary hypocalcemia. Proc Natl Acad Sci U S A 2004;101:2894-2899.

5 Komiya Y, Runnels LW: TRPM channels and magnesium in early embryonic development. Int J Dev Biol 2015;59:281-288.

6 Schmitz C, Perraud AL, Johnson CO, Inabe K, Smith MK, Penner R, Kurosaki T, Fleig A, Scharenberg AM: Regulation of vertebrate cellular Mg2+ homeostasis by TRPM7. Cell 2003;114:191-200.

7 Jin J, Desai BN, Navarro B, Donovan A, Andrews NC, Clapham DE: Deletion of Trpm7 disrupts embryonic development and thymopoiesis without altering Mg2+ homeostasis. Science 2008;322:756-760.

8 Mittermeier L, Demirkhanyan L, Stadlbauer B, Breit A, Recordati C, Hilgendorff A, Matsushita M, Braun A, Simmons DG, Zakharian E, Gudermann T, Chubanov V: TRPM7 is the central gatekeeper of intestinal mineral absorption essential for postnatal survival. Proceedings of the National Academy of Sciences of the United States of America 2019

9 Ryazanova LV, Rondon LJ, Zierler S, Hu Z, Galli J, Yamaguchi TP, Mazur A, Fleig A, Ryazanov AG: TRPM7 is essential for Mg(2+) homeostasis in mammals. Nat Commun 2010;1:109.

10 Monteilh-Zoller MK, Hermosura MC, Nadler MJ, Scharenberg AM, Penner R, Fleig A: TRPM7 provides an ion channel mechanism for cellular entry of trace metal ions. J Gen Physiol 2003;121:49-60.

11 Li M, Jiang J, Yue L: Functional characterization of homo- and heteromeric channel kinases TRPM6 and TRPM7. J Gen Physiol 2006;127:525-537.

12 Chubanov V, Köttgen M, Touyz RM, Gudermann T: TRPM channels in health and disease. Nature Reviews Nephrology 2023

13 Meng S, Alanazi R, Ji D, Bandura J, Luo ZW, Fleig A, Feng ZP, Sun HS: Role of TRPM7 kinase in cancer. Cell Calcium 2021;96:102400.

14 Ryazanov AG, Ward MD, Mendola CE, Pavur KS, Dorovkov MV, Wiedmann M, Erdjument-Bromage H, Tempst P, Parmer TG, Prostko CR, Germino FJ, Hait WN: Identification of a new class of protein kinases represented by eukaryotic elongation factor-2 kinase. Proc Natl Acad Sci U S A 1997;94:4884-4889.

15 Middelbeek J, Clark K, Venselaar H, Huynen MA, van Leeuwen FN: The alpha-kinase family: an exceptional branch on the protein kinase tree. Cell Mol Life Sci 2010;67:875-890.

16 Ryazanov AG, Pavur KS, Dorovkov MV: Alpha-kinases: a new class of protein kinases with a novel catalytic domain. Curr Biol 1999;9:R43-45.

17 Yamaguchi H, Matsushita M, Nairn AC, Kuriyan J: Crystal structure of the atypical protein kinase domain of a TRP channel with phosphotransferase activity. Mol Cell 2001;7:1047-1057.

18 Duan J, Li Z, Li J, Hulse RE, Santa-Cruz A, Valinsky WC, Abiria SA, Krapivinsky G, Zhang J, Clapham DE: Structure of the mammalian TRPM7, a magnesium channel required during embryonic development. Proceedings of the National Academy of Sciences of the United States of America 2018;115:E8201-E8210.

19 Schmidt E, Narangoda C, Nörenberg W, Egawa M, Rössig A, Leonhardt M, Schaefer M, Zierler S, Kurnikova MG, Gudermann T, Chubanov V: Structural mechanism of TRPM7 channel regulation by intracellular magnesium. Cellular and Molecular Life Sciences 2022;79

20 Nadezhdin KD, Correia L, Narangoda C, Patel DS, Neuberger A, Gudermann T, Kurnikova MG, Chubanov V, Sobolevsky AI: Structural mechanisms of TRPM7 activation and inhibition. Nature Communications 2023;14

21 Nadezhdin KD, Correia L, Shalygin A, Aktolun M, Neuberger A, Gudermann T, Kurnikova MG, Chubanov V, Sobolevsky AI: Structural basis of selective TRPM7 inhibition by the anticancer agent CCT128930. Cell Rep 2024;43:114108.

22 Ryazanova LV, Hu Z, Suzuki S, Chubanov V, Fleig A, Ryazanov AG: Elucidating the role of the TRPM7 alpha-kinase: TRPM7 kinase inactivation leads to magnesium deprivation resistance phenotype in mice. Sci Rep 2014;4:7599.

23 Kaitsuka T, Katagiri C, Beesetty P, Nakamura K, Hourani S, Tomizawa K, Kozak JA, Matsushita M: Inactivation of TRPM7 kinase activity does not impair its channel function in mice. Scientific reports 2014;4:5718.

24 Hoeger B, Nadolni W, Hampe S, Hoelting K, Fraticelli M, Zaborsky N, Madlmayr A, Sperrer V, Fraticelli L, Addington L, Steinritz D, Chubanov V, Geisberger R, Greil R, Breit A, Boekhoff I, Gudermann T, Zierler S: Inactivation of TRPM7 Kinase Targets AKT Signaling and Cyclooxygenase-2 Expression in Human CML Cells. Function (Oxf) 2023;4:zqad053.

25 Lim LH, Pervaiz S: Annexin 1: the new face of an old molecule. FASEB J 2007;21:968-975.

26 Dorovkov MV, Ryazanov AG: Phosphorylation of annexin I by TRPM7 channel-kinase. J Biol Chem 2004;279:50643-50646.

27 Dorovkov MV, Kostyukova AS, Ryazanov AG: Phosphorylation of annexin A1 by TRPM7 kinase: a switch regulating the induction of an alpha-helix. Biochemistry 2011;50:2187-2193.

28 Bousova K, Zouharova M, Herman P, Vetyskova V, Jiraskova K, Vondrasek J: TRPM7 N-terminal region forms complexes with calcium binding proteins CaM and S100A1. Heliyon 2021;7:e08490.

29 Mishra R, Rao V, Ta R, Shobeiri N, Hill CE: Mg2+- and MgATP-inhibited and Ca2+/calmodulin-sensitive TRPM7-like current in hepatoma and hepatocytes. Am J Physiol Gastrointest Liver Physiol 2009;297:G687-694.

30 Turlova E, Wong R, Xu B, Li F, Du L, Habbous S, Horgen FD, Fleig A, Feng ZP, Sun HS: TRPM7 Mediates Neuronal Cell Death Upstream of Calcium/Calmodulin-Dependent Protein Kinase II and Calcineurin Mechanism in Neonatal Hypoxic-Ischemic Brain Injury. Transl Stroke Res 2021;12:164-184.

31 de Baaij JH, Hoenderop JG, Bindels RJ: Magnesium in man: implications for health and disease. Physiol Rev 2015;95:1-46.

32 Bai Z, Feng J, Franken GAC, Al'Saadi N, Cai N, Yu AS, Lou L, Komiya Y, Hoenderop JGJ, de Baaij JHF, Yue L, Runnels LW: CNNM proteins selectively bind to the TRPM7 channel to stimulate divalent cation entry into cells. PLoS Biol 2021;19:e3001496.

33 Kollewe A, Chubanov V, Tseung FT, Correia L, Schmidt E, Rossig A, Zierler S, Haupt A, Muller CS, Bildl W, Schulte U, Nicke A, Fakler B, Gudermann T: The molecular appearance of native TRPM7 channel complexes identified by high-resolution proteomics. Elife 2021;10

34 Mahbub L, Kozlov G, Zong P, Lee EL, Tetteh S, Nethramangalath T, Knorn C, Jiang J, Shahsavan A, Yue L, Runnels L, Gehring K: Structural insights into regulation of CNNM-TRPM7 divalent cation uptake by the small GTPase ARL15. Elife 2023;12

35 de Baaij JH, Stuiver M, Meij IC, Lainez S, Kopplin K, Venselaar H, Muller D, Bindels RJ, Hoenderop JG: Membrane topology and intracellular processing of cyclin M2 (CNNM2). J Biol Chem 2012;287:13644-13655.

36 Jolly JT, Blackburn JS: The PACT Network: PRL, ARL, CNNM, and TRPM Proteins in Magnesium Transport and Disease. Int J Mol Sci 2025;26

37 Zolotarov Y, Ma C, González-Recio I, Hardy S, Franken GAC, Uetani N, Latta F, Kostantin E, Boulais J, Thibault M-P, Côté J-F, Díaz-Moreno I, Quintana AD, Hoenderop JGJ, Martínez-Cruz LA, Tremblay ML, De Baaij JHF: ARL15 modulates magnesium homeostasis through N-glycosylation of CNNMs. Cellular and Molecular Life Sciences 2021;78:5427-5445.

38 Tetteh S, Zong P, Feng J, Lee EL, Al-Saadi N, Willekens J, Shah A, Nethramangalath T, Galeano AL, Zheng H, Gehring K, Yue L, Runnels LW: The cytoplasmic domains of the CNNM family of transmembrane proteins modulate the ion channel-kinase TRPM7. J Biol Chem 2025;301:110720.

39 Hardy S, Zolotarov Y, Coleman J, Roitman S, Khursheed H, Aubry I, Uetani N, Tremblay ML: PRL-1/2 phosphatases control TRPM7 magnesium-dependent function to regulate cellular bioenergetics. Proc Natl Acad Sci U S A 2023;120:e2221083120.

40 Funato Y, Yamazaki D, Mizukami S, Du L, Kikuchi K, Miki H: Membrane protein CNNM4-dependent Mg2+ efflux suppresses tumor progression. The Journal of clinical investigation 2014;124:5398-5410.

41 Hardy S, Wong NN, Muller WJ, Park M, Tremblay ML: Overexpression of the protein tyrosine phosphatase PRL-2 correlates with breast tumor formation and progression. Cancer Res 2010;70:8959-8967.

42 Hardy S, Uetani N, Wong N, Kostantin E, Labbe DP, Begin LR, Mes-Masson A, Miranda-Saavedra D, Tremblay ML: The protein tyrosine phosphatase PRL-2 interacts with the magnesium transporter CNNM3 to promote oncogenesis. Oncogene 2015;34:986-995.

43 Auwercx J, Rybarczyk P, Kischel P, Dhennin-Duthille I, Chatelain D, Sevestre H, Van Seuningen I, Ouadid-Ahidouch H, Jonckheere N, Gautier M: Mg(2+) Transporters in Digestive Cancers. Nutrients 2021;13

44 Song C, Bae Y, Jun J, Lee H, Kim ND, Lee KB, Hur W, Park JY, Sim T: Identification of TG100-115 as a new and potent TRPM7 kinase inhibitor, which suppresses breast cancer cell migration and invasion. Biochimica et biophysica acta 2017;1861:947-957.

45 Ogata K, Tsumuraya T, Oka K, Shin M, Okamoto F, Kajiya H, Katagiri C, Ozaki M, Matsushita M, Okabe K: The crucial role of the TRPM7 kinase domain in the early stage of amelogenesis. Sci Rep 2017;7:18099.

46 Wu W, Wang X, Liao L, Chen J, Wang Y, Yao M, Zhu L, Li J, Wang X, Chen AF, Zhang G, Zhang Z, Bai Y: The TRPM7 channel reprograms cellular glycolysis to drive tumorigenesis and angiogenesis. Cell Death Dis 2023;14:183.

47 Perraud AL, Zhao X, Ryazanov AG, Schmitz C: The channel-kinase TRPM7 regulates phosphorylation of the translational factor eEF2 via eEF2-k. Cell Signal 2011;23:586-593.

48 Zou ZG, Rios FJ, Neves KB, Alves-Lopes R, Ling J, Baillie GS, Gao X, Fuller W, Camargo LL, Gudermann T, Chubanov V, Montezano AC, Touyz RM: Epidermal growth factor signaling through transient receptor potential melastatin 7 cation channel regulates vascular smooth muscle cell function. Clin Sci (Lond) 2020;134:2019-2035.

49 Desai BN, Krapivinsky G, Navarro B, Krapivinsky L, Carter BC, Febvay S, Delling M, Penumaka A, Ramsey IS, Manasian Y, Clapham DE: Cleavage of TRPM7 releases the kinase domain from the ion channel and regulates its participation in Fas-induced apoptosis. Dev Cell 2012;22:1149-1162.

50 Krapivinsky G, Krapivinsky L, Manasian Y, Clapham DE: The TRPM7 chanzyme is cleaved to release a chromatin-modifying kinase. Cell 2014;157:1061-1072.

51 Matsushita M, Kozak JA, Shimizu Y, McLachlin DT, Yamaguchi H, Wei FY, Tomizawa K, Matsui H, Chait BT, Cahalan MD, Nairn AC: Channel function is dissociated from the intrinsic kinase activity and autophosphorylation of TRPM7/ChaK1. J Biol Chem 2005;280:20793-20803.

52 van Leeuwen FN, van Delft S, Kain HE, van der Kammen RA, Collard JG: Rac regulates phosphorylation of the myosin-II heavy chain, actinomyosin disassembly and cell spreading. Nat Cell Biol 1999;1:242-248.

53 Clark K, Langeslag M, van Leeuwen B, Ran L, Ryazanov AG, Figdor CG, Moolenaar WH, Jalink K, van Leeuwen FN: TRPM7, a novel regulator of actomyosin contractility and cell adhesion. EMBO J 2006;25:290-301.

54 Clark K, Middelbeek J, Lasonder E, Dulyaninova NG, Morrice NA, Ryazanov AG, Bresnick AR, Figdor CG, van Leeuwen FN: TRPM7 regulates myosin IIA filament stability and protein localization by heavy chain phosphorylation. J Mol Biol 2008;378:790-803.

55 Clark K, Middelbeek J, Dorovkov MV, Figdor CG, Ryazanov AG, Lasonder E, van Leeuwen FN: The alpha-kinases TRPM6 and TRPM7, but not eEF-2 kinase, phosphorylate the assembly domain of myosin IIA, IIB and IIC. FEBS Lett 2008;582:2993-2997.

56 Krapivinsky G, Mochida S, Krapivinsky L, Cibulsky SM, Clapham DE: The TRPM7 ion channel functions in cholinergic synaptic vesicles and affects transmitter release. Neuron 2006;52:485-496.

57 Auwercx J, Neve B, Vanlaeys A, Fourgeaud M, Bourrin-Reynard I, Souidi M, Brassart-Pasco S, Hague F, Guenin S, Duchene B, Gutierrez L, Destaing O, Dhennin-Duthille I, Van Seuningen I, Jonckheere N, Gautier M: The kinase domain of TRPM7 interacts with PAK1 and regulates pancreatic cancer cell epithelial-to-mesenchymal transition. Cell Death Dis 2025;16:335.

58 Runnels LW, Yue L, Clapham DE: The TRPM7 channel is inactivated by PIP(2) hydrolysis. Nat Cell Biol 2002;4:329-336.

59 Langeslag M, Clark K, Moolenaar WH, van Leeuwen FN, Jalink K: Activation of TRPM7 channels by phospholipase C-coupled receptor agonists. J Biol Chem 2007;282:232-239.

60 Gotru SK, Chen W, Kraft P, Becker IC, Wolf K, Stritt S, Zierler S, Hermanns HM, Rao D, Perraud AL, Schmitz C, Zahedi RP, Noy PJ, Tomlinson MG, Dandekar T, Matsushita M, Chubanov V, Gudermann T, Stoll G, Nieswandt B, Braun A: TRPM7 Kinase Controls Calcium Responses in Arterial Thrombosis and Stroke in Mice. Arterioscler Thromb Vasc Biol 2018;38:344-352.

61 Voringer S, Schreyer L, Nadolni W, Meier MA, Woerther K, Mittermeier C, Ferioli S, Singer S, Holzer K, Zierler S, Chubanov V, Liebl B, Gudermann T, Muehlich S: Inhibition of TRPM7 blocks MRTF/SRF-dependent transcriptional and tumorigenic activity. Oncogene 2020;39:2328-2344.

62 Romagnani A, Vettore V, Rezzonico-Jost T, Hampe S, Rottoli E, Nadolni W, Perotti M, Meier MA, Hermanns C, Geiger S, Wennemuth G, Recordati C, Matsushita M, Muehlich S, Proietti M, Chubanov V, Gudermann T, Grassi F, Zierler S: TRPM7 kinase activity is essential for T cell colonization and alloreactivity in the gut. Nature communications 2017;8:1917.

63 Faouzi M, Kilch T, Horgen FD, Fleig A, Penner R: The TRPM7 channel kinase regulates store-operated calcium entry. J Physiol 2017;595:3165-3180.

64 Dorovkov MV, Beznosov SN, Shah S, Kotlianskaia L, Kostiukova AS: [Effect of mutations imitating the phosphorylation by TRPM7 kinase on the function of the N-terminal domain of tropomodulin]. Biofizika 2008;53:943-949.

65 Chubanov V, Gudermann T: Trpm6. Handb Exp Pharmacol 2014;222:503-520.

66 Schlingmann KP, Weber S, Peters M, Niemann Nejsum L, Vitzthum H, Klingel K, Kratz M, Haddad E, Ristoff E, Dinour D, Syrrou M, Nielsen S, Sassen M, Waldegger S, Seyberth HW, Konrad M: Hypomagnesemia with secondary hypocalcemia is caused by mutations in TRPM6, a new member of the TRPM gene family. Nat Genet 2002;31:166-170.

67 Clark K, Middelbeek J, Morrice NA, Figdor CG, Lasonder E, van Leeuwen FN: Massive autophosphorylation of the Ser/Thr-rich domain controls protein kinase activity of TRPM6 and TRPM7. PLoS One 2008;3:e1876.

68 Oughtred R, Rust J, Chang C, Breitkreutz BJ, Stark C, Willems A, Boucher L, Leung G, Kolas N, Zhang F, Dolma S, Coulombe-Huntington J, Chatr-Aryamontri A, Dolinski K, Tyers M: The BioGRID database: A comprehensive biomedical resource of curated protein, genetic, and chemical interactions. Protein Sci 2021;30:187-200.

69 Roux KJ, Kim DI, Burke B, May DG: BioID: A Screen for Protein-Protein Interactions. Curr Protoc Protein Sci 2018;91:19 23 11-19 23 15.

70 Viard J, Loe-Mie Y, Daudin R, Khelfaoui M, Plancon C, Boland A, Tejedor F, Huganir RL, Kim E, Kinoshita M, Liu G, Haucke V, Moncion T, Yu E, Hindie V, Blehaut H, Mircher C, Herault Y, Deleuze JF, Rain JC, Simonneau M, Lepagnol-Bestel AM: Chr21 protein-protein interactions: enrichment in proteins involved in intellectual disability, autism, and late-onset Alzheimer's disease. Life Sci Alliance 2022;5

71 Horlbeck MA, Xu A, Wang M, Bennett NK, Park CY, Bogdanoff D, Adamson B, Chow ED, Kampmann M, Peterson TR, Nakamura K, Fischbach MA, Weissman JS, Gilbert LA: Mapping the Genetic Landscape of Human Cells. Cell 2018;174:953-967 e922.

72 Vichas A, Riley AK, Nkinsi NT, Kamlapurkar S, Parrish PCR, Lo A, Duke F, Chen J, Fung I, Watson J, Rees M, Gabel AM, Thomas JD, Bradley RK, Lee JK, Hatch EM, Baine MK, Rekhtman N, Ladanyi M, Piccioni F, Berger AH: Integrative oncogene-dependency mapping identifies RIT1 vulnerabilities and synergies in lung cancer. Nat Commun 2021;12:4789.

73 Baxter JS, Brough R, Krastev DB, Song F, Sridhar S, Gulati A, Alexander J, Roumeliotis TI, Kozik Z, Choudhary JS, Haider S, Pettitt SJ, Tutt ANJ, Lord CJ: Cancer-associated FBXW7 loss is synthetic lethal with pharmacological targeting of CDC7. Mol Oncol 2024;18:369-385.

74 Aregger M, Lawson KA, Billmann M, Costanzo M, Tong AHY, Chan K, Rahman M, Brown KR, Ross C, Usaj M, Nedyalkova L, Sizova O, Habsid A, Pawling J, Lin ZY, Abdouni H, Wong CJ, Weiss A, Mero P, Dennis JW, Gingras AC, Myers CL, Andrews BJ, Boone C, Moffat J: Systematic mapping of genetic interactions for de novo fatty acid synthesis identifies C12orf49 as a regulator of lipid metabolism. Nat Metab 2020;2:499-513.

75 Huttlin EL, Ting L, Bruckner RJ, Gebreab F, Gygi MP, Szpyt J, Tam S, Zarraga G, Colby G, Baltier K, Dong R, Guarani V, Vaites LP, Ordureau A, Rad R, Erickson BK, Wuhr M, Chick J, Zhai B, Kolippakkam D, Mintseris J, Obar RA, Harris T, Artavanis-Tsakonas S, Sowa ME, De Camilli P, Paulo JA, Harper JW, Gygi SP: The BioPlex Network: A Systematic Exploration of the Human Interactome. Cell 2015;162:425-440.

76 Gupta GD, Coyaud E, Goncalves J, Mojarad BA, Liu Y, Wu Q, Gheiratmand L, Comartin D, Tkach JM, Cheung SW, Bashkurov M, Hasegan M, Knight JD, Lin ZY, Schueler M, Hildebrandt F, Moffat J, Gingras AC, Raught B, Pelletier L: A Dynamic Protein Interaction Landscape of the Human Centrosome-Cilium Interface. Cell 2015;163:1484-1499.

77 Hein MY, Hubner NC, Poser I, Cox J, Nagaraj N, Toyoda Y, Gak IA, Weisswange I, Mansfeld J, Buchholz F, Hyman AA, Mann M: A human interactome in three quantitative dimensions organized by stoichiometries and abundances. Cell 2015;163:712-723.

78 Huttlin EL, Bruckner RJ, Paulo JA, Cannon JR, Ting L, Baltier K, Colby G, Gebreab F, Gygi MP, Parzen H, Szpyt J, Tam S, Zarraga G, Pontano-Vaites L, Swarup S, White AE, Schweppe DK, Rad R, Erickson BK, Obar RA, Guruharsha KG, Li K, Artavanis-Tsakonas S, Gygi SP, Harper JW: Architecture of the human interactome defines protein communities and disease networks. Nature 2017;545:505-509.

79 Choudhury NR, Heikel G, Trubitsyna M, Kubik P, Nowak JS, Webb S, Granneman S, Spanos C, Rappsilber J, Castello A, Michlewski G: RNA-binding activity of TRIM25 is mediated by its PRY/SPRY domain and is required for ubiquitination. BMC Biol 2017;15:105.

80 Davis ZH, Verschueren E, Jang GM, Kleffman K, Johnson JR, Park J, Von Dollen J, Maher MC, Johnson T, Newton W, Jager S, Shales M, Horner J, Hernandez RD, Krogan NJ, Glaunsinger BA: Global mapping of herpesvirus-host protein complexes reveals a transcription strategy for late genes. Mol Cell 2015;57:349-360.

81 Kovalski JR, Bhaduri A, Zehnder AM, Neela PH, Che Y, Wozniak GG, Khavari PA: The Functional Proximal Proteome of Oncogenic Ras Includes mTORC2. Mol Cell 2019;73:830-844 e812.

82 Liu X, Salokas K, Tamene F, Jiu Y, Weldatsadik RG, Ohman T, Varjosalo M: An AP-MS- and BioID-compatible MAC-tag enables comprehensive mapping of protein interactions and subcellular localizations. Nat Commun 2018;9:1188.

83 Swayampakula M, McDonald PC, Vallejo M, Coyaud E, Chafe SC, Westerback A, Venkateswaran G, Shankar J, Gao G, Laurent EMN, Lou Y, Bennewith KL, Supuran CT, Nabi IR, Raught B, Dedhar S: The interactome of metabolic enzyme carbonic anhydrase IX reveals novel roles in tumor cell migration and invadopodia/MMP14-mediated invasion. Oncogene 2017;36:6244-6261.

84 Ikeda KN, Freeman M: Spatial proteomics reveal that the protein phosphatase PTP1B interacts with and may modify tyrosine phosphorylation of the rhomboid protease RHBDL4. J Biol Chem 2019;294:11486-11497.

85 Ceccarelli DF, Ivantsiv S, Mullin AA, Coyaud E, Manczyk N, Maisonneuve P, Kurinov I, Zhao L, Go C, Gingras AC, Raught B, Cordes S, Sicheri F: FAM105A/OTULINL Is a Pseudodeubiquitinase of the OTU-Class that Localizes to the ER Membrane. Structure 2019;27:1000-1012 e1006.

86 Fasci D, van Ingen H, Scheltema RA, Heck AJR: Histone Interaction Landscapes Visualized by Crosslinking Mass Spectrometry in Intact Cell Nuclei. Mol Cell Proteomics 2018;17:2018-2033.

87 Samavarchi-Tehrani P: A SARS-CoV-2 – host proximity interactome. bioRxiv 2020

88 St-Germain JR, Astori A, Raught B: A SARS-CoV-2 Peptide Spectral Library Enables Rapid, Sensitive Identification of Virus Peptides in Complex Biological Samples. J Proteome Res 2021;20:2187-2194.

89 Youn JY, Dunham WH, Hong SJ, Knight JDR, Bashkurov M, Chen GI, Bagci H, Rathod B, MacLeod G, Eng SWM, Angers S, Morris Q, Fabian M, Cote JF, Gingras AC: High-Density Proximity Mapping Reveals the Subcellular Organization of mRNA-Associated Granules and Bodies. Mol Cell 2018;69:517-532 e511.

90 Antonicka H, Lin ZY, Janer A, Aaltonen MJ, Weraarpachai W, Gingras AC, Shoubridge EA: A High-Density Human Mitochondrial Proximity Interaction Network. Cell Metab 2020;32:479-497 e479.

91 Hannigan MM, Hoffman AM, Thompson JW, Zheng T, Nicchitta CV: Quantitative Proteomics Links the LRRC59 Interactome to mRNA Translation on the ER Membrane. Mol Cell Proteomics 2020;19:1826-1849.

92 Piette BL, Alerasool N, Lin ZY, Lacoste J, Lam MHY, Qian WW, Tran S, Larsen B, Campos E, Peng J, Gingras AC, Taipale M: Comprehensive interactome profiling of the human Hsp70 network highlights functional differentiation of J domains. Mol Cell 2021;81:2549-2565 e2548.

93 Qiu R, Yu X, Wang L, Han Z, Yao C, Cui Y, Hou G, Dai D, Jin W, Shen N: Inhibition of Glycolysis in Pathogenic T(H)17 Cells through Targeting a miR -21-Peli1-c-Rel Pathway Prevents Autoimmunity. J Immunol 2020;204:3160-3170.

94 Go CD, Knight JDR, Rajasekharan A, Rathod B, Hesketh GG, Abe KT, Youn JY, Samavarchi-Tehrani P, Zhang H, Zhu LY, Popiel E, Lambert JP, Coyaud E, Cheung SWT, Rajendran D, Wong CJ, Antonicka H, Pelletier L, Palazzo AF, Shoubridge EA, Raught B, Gingras AC: A proximity-dependent biotinylation map of a human cell. Nature 2021;595:120-124.

95 Huttlin EL, Bruckner RJ, Navarrete-Perea J, Cannon JR, Baltier K, Gebreab F, Gygi MP, Thornock A, Zarraga G, Tam S, Szpyt J, Gassaway BM, Panov A, Parzen H, Fu S, Golbazi A, Maenpaa E, Stricker K, Guha Thakurta S, Zhang T, Rad R, Pan J, Nusinow DP, Paulo JA, Schweppe DK, Vaites LP, Harper JW, Gygi SP: Dual proteome-scale networks reveal cell-specific remodeling of the human interactome. Cell 2021;184:3022-3040 e3028.

96 Liu X, Huuskonen S, Laitinen T, Redchuk T, Bogacheva M, Salokas K, Pohner I, Ohman T, Tonduru AK, Hassinen A, Gawriyski L, Keskitalo S, Vartiainen MK, Pietiainen V, Poso A, Varjosalo M: SARS-CoV-2-host proteome interactions for antiviral drug discovery. Mol Syst Biol 2021;17:e10396.

97 Bagci H, Sriskandarajah N, Robert A, Boulais J, Elkholi IE, Tran V, Lin ZY, Thibault MP, Dube N, Faubert D, Hipfner DR, Gingras AC, Cote JF: Mapping the proximity interaction network of the Rho-family GTPases reveals signalling pathways and regulatory mechanisms. Nat Cell Biol 2020;22:120-134.

98 Yan BR, Li T, Coyaud E, Laurent EMN, St-Germain J, Zhou Y, Kim PK, Raught B, Brumell JH: C5orf51 is a component of the MON1-CCZ1 complex and controls RAB7A localization and stability during mitophagy. Autophagy 2022;18:829-840.

99 Li FL, Wu Z, Gao YQ, Bowling FZ, Franklin JM, Hu C, Suhandynata RT, Frohman MA, Airola MV, Zhou H, Guan KL: Defining the proximal interaction networks of Arf GTPases reveals a mechanism for the regulation of PLD1 and PI4KB. EMBO J 2022;41:e110698.

100 Salokas K, Liu X, Ohman T, Chowdhury I, Gawriyski L, Keskitalo S, Varjosalo M: Physical and functional interactome atlas of human receptor tyrosine kinases. EMBO Rep 2022;23:e54041.

101 Segal D, Maier S, Mastromarco GJ, Qian WW, Nabeel-Shah S, Lee H, Moore G, Lacoste J, Larsen B, Lin ZY, Selvabaskaran A, Liu K, Smibert C, Zhang Z, Greenblatt J, Peng J, Lee HO, Gingras AC, Taipale M: A central chaperone-like role for 14-3-3 proteins in human cells. Mol Cell 2023;83:974-993 e915.

102 Kiss RS, Chicoine J, Khalil Y, Sladek R, Chen H, Pisaturo A, Martin C, Dale JD, Brudenell TA, Kamath A, Kyei-Boahen J, Hafiane A, Daliah G, Alecki C, Hopes TS, Heier M, Aligianis IA, Lebrun JJ, Aspden J, Paci E, Kerksiek A, Lutjohann D, Clayton P, Wills JC, von Kriegsheim A, Nilsson T, Sheridan E, Handley MT: Comparative proximity biotinylation implicates the small GTPase RAB18 in sterol mobilization and biosynthesis. J Biol Chem 2023;299:105295.

103 Janer A, Morris JL, Krols M, Antonicka H, Aaltonen MJ, Lin ZY, Anand H, Gingras AC, Prudent J, Shoubridge EA: ESYT1 tethers the ER to mitochondria and is required for mitochondrial lipid and calcium homeostasis. Life Sci Alliance 2024;7

104 Miller SG, Hoh M, Ebmeier CC, Tay JW, Ahn NG: Cooperative polarization of MCAM/CD146 and ERM family proteins in melanoma. Mol Biol Cell 2024;35:ar31.

105 Szklarczyk D, Kirsch R, Koutrouli M, Nastou K, Mehryary F, Hachilif R, Gable AL, Fang T, Doncheva NT, Pyysalo S, Bork P, Jensen LJ, von Mering C: The STRING database in 2023: protein-protein association networks and functional enrichment analyses for any sequenced genome of interest. Nucleic Acids Res 2023;51:D638-D646.

106 Ge SX, Jung D, Yao R: ShinyGO: a graphical gene-set enrichment tool for animals and plants. Bioinformatics 2020;36:2628-2629.

107 Canzler S, Fischer M, Ulbricht D, Ristic N, Hildebrand PW, Staritzbichler R: ProteinPrompt: a webserver for predicting protein-protein interactions. Bioinform Adv 2022;2:vbac059.

108 Najjar MK, Khan MS, Zhuang C, Chandra A, Lo HW: Interleukin-1 Receptor-Associated Kinase 1 in Cancer Metastasis and Therapeutic Resistance: Mechanistic Insights and Translational Advances. Cells 2024;13

109 Guan Z, Chen X, Fang S, Ji Y, Gao Z, Zheng Y: CCT128930 is a novel and potent antagonist of TRPM7 channel. Biochem Biophys Res Commun 2021;560:132-138.

110 Chen L, Zhang J, Wang X, Li Y, Zhou L, Lu X, Dong G, Sheng C: Discovery of novel KRAS‒PDEdelta inhibitors with potent activity in patient-derived human pancreatic tumor xenograft models. Acta Pharm Sin B 2022;12:274-290.

111 Kessler D, Gmachl M, Mantoulidis A, Martin LJ, Zoephel A, Mayer M, Gollner A, Covini D, Fischer S, Gerstberger T, Gmaschitz T, Goodwin C, Greb P, Haring D, Hela W, Hoffmann J, Karolyi-Oezguer J, Knesl P, Kornigg S, Koegl M, Kousek R, Lamarre L, Moser F, Munico-Martinez S, Peinsipp C, Phan J, Rinnenthal J, Sai J, Salamon C, Scherbantin Y, Schipany K, Schnitzer R, Schrenk A, Sharps B, Siszler G, Sun Q, Waterson A, Wolkerstorfer B, Zeeb M, Pearson M, Fesik SW, McConnell DB: Drugging an undruggable pocket on KRAS. Proc Natl Acad Sci U S A 2019;116:15823-15829.

112 Afonso AL, Cavaleiro CT, Castanho M, Neves V, Cavaco M: The Potential of Peptide-Based Inhibitors in Disrupting Protein-Protein Interactions for Targeted Cancer Therapy. Int J Mol Sci 2025;26

**Supplemental Data:**

**Table S1:** List of TRPM7 interactors predicted using ProteinPrompt.

| Protein | Interation score  (Interaction = 1) | Uniprot ID | | Full description |
| --- | --- | --- | --- | --- |
| TRPM7 | 1.0000 | | Q96QT4 | Transient receptor potential cation channel subfamily M member 7 |
| EF2 | 0.9907 | | P13639 | Elongation factor 2 |
| H31T | 0.9733 | | Q16695 | Histone H3.1t |
| H31 | 0.9520 | | P68431 | Histone H3.1 |
| CALM | 0.9387 | | P62158 | Calmodulin |
| TRPM6 | 0.9360 | | Q9BX84 | Transient receptor potential cation channel subfamily M member 6 |
| Q59F77 | 0.9333 | | Q59F77 | Phosphoinositide phospholipase C |
| PLCB2 | 0.9333 | | Q00722 | 1-phosphatidylinositol 4,5-bisphosphate phosphodiesterase beta-2 |
| KR412 | 0.9293 | | Q9BQ66 | Keratin-associated protein 4-12 |
| DYL1 | 0.9293 | | P63167 | Dynein light chain 1, cytoplasmic |
| Q9UFY1 | 0.9280 | | Q9UFY1 | Phosphoinositide phospholipase C |
| PLCG1 | 0.9280 | | P19174 | 1-phosphatidylinositol 4,5-bisphosphate phosphodiesterase gamma-1 |
| A4D2P0 | 0.9227 | | A4D2P0 | RAC1 {ECO:0000313\|EMBL:EAL23718.1} |
| UBB | 0.9173 | | P0CG47 | Polyubiquitin-B precursor |
| Q5U5U6 | 0.9173 | | Q5U5U6 | Epididymis secretory protein Li 50 |
| Q658J6 | 0.9120 | | Q658J6 | DKFZp762B153 {ECO:0000313\|EMBL:CAD38970.1} |
| MLP3B | 0.9120 | | Q9GZQ8 | Microtubule-associated proteins 1A/1B light chain 3B precursor |
| H32 | 0.9093 | | Q71DI3 | Histone H3.2 |
| RL40 | 0.9067 | | P62987 | Ubiquitin-60S ribosomal protein L40 precursor |
| Q6I9R8 | 0.9067 | | Q6I9R8 | FHL2 {ECO:0000313\|EMBL:CAG33718.1} |
| FHL2 | 0.9067 | | Q14192 | Four and a half LIM domains protein 2 |
| Q53YK7 | 0.9053 | | Q53YK7 | PRNP {ECO:0000313\|EMBL:AAS80162.1} |
| PRIO | 0.9053 | | P04156 | Major prion protein precursor |
| H2B2E | 0.9027 | | Q16778 | Histone H2B type 2-E |
| H33 | 0.9013 | | P84243 | Histone H3.3 |
| B2R4P9 | 0.9013 | | B2R4P9 | Histone H3 |
| RASH | 0.8973 | | P01112 | GTPase HRas precursor |
| PTN | 0.8973 | | P21246 | Pleiotrophin precursor |
| H2B1C | 0.8947 | | P62807 | Histone H2B type 1-C/E/F/G/I |
| GBRL2 | 0.8947 | | P60520 | Gamma-aminobutyric acid receptor-associated protein-like 2 precursor |
| CRK | 0.8947 | | P46108 | Adapter molecule crk |
| B2R4S9 | 0.8947 | | B2R4S9 | HIST1H2BE {ECO:0000313\|EMBL:EAW55537.1} |
| Q6IT96 | 0.8933 | | Q6IT96 | HDAC1 {ECO:0000313\|EMBL:BAG70244.1} |
| HDAC1 | 0.8933 | | Q13547 | Histone deacetylase 1 |
| GRB2 | 0.8907 | | P62993 | Growth factor receptor-bound protein 2 |
| H2AX | 0.8880 | | P16104 | Histone H2AX |
| P53 | 0.8867 | | P04637 | Cellular tumor antigen p53 |
| CDK1 | 0.8867 | | P06493 | Cyclin-dependent kinase 1 |
| CALX | 0.8853 | | P27824 | Calnexin precursor |
| PARP1 | 0.8827 | | P09874 | Poly [ADP-ribose] polymerase 1 |
| P85A | 0.8827 | | P27986 | Phosphatidylinositol 3-kinase regulatory subunit alpha |
| NCK1 | 0.8827 | | P16333 | Cytoplasmic protein NCK1 |
| H4 | 0.8827 | | P62805 | Histone H4 |
| B2R4R0 | 0.8827 | | B2R4R0 | Histone H4 |
| UBC9 | 0.8813 | | P63279 | SUMO-conjugating enzyme UBC9 |
| RAC1 | 0.8813 | | P63000 | Ras-related C3 botulinum toxin substrate 1 precursor |
| CASP3 | 0.8813 | | P42574 | Caspase-3 precursor |
| ABL1 | 0.8813 | | P00519 | Tyrosine-protein kinase ABL1 |
| Q96C32 | 0.8787 | | Q96C32 | Polyubiquitin-C |
| Q6FII3 | 0.8787 | | Q6FII3 | C14orf1 {ECO:0000313\|EMBL:CAG38474.1} |
| ERG28 | 0.8787 | | Q9UKR5 | Probable ergosterol biosynthetic protein 28 |
| CDK2 | 0.8787 | | P24941 | Cyclin-dependent kinase 2 |
| CDC42 | 0.8787 | | P60953 | Cell division control protein 42 homolog precursor |
| B0LPE5 | 0.8787 | | B0LPE5 | AKT1 {ECO:0000313\|EMBL:ABY87524.1} |
| ATX1 | 0.8787 | | P54253 | Ataxin-1 |
| AKT1 | 0.8787 | | P31749 | RAC-alpha serine/threonine-protein kinase |
| 4EBP1 | 0.8773 | | Q13541 | Eukaryotic translation initiation factor 4E-binding protein 1 |
| SUMO2 | 0.8760 | | P61956 | Small ubiquitin-related modifier 2 precursor |
| STX1A | 0.8760 | | Q16623 | Syntaxin-1A |
| RBX1 | 0.8760 | | P62877 | E3 ubiquitin-protein ligase RBX1 |
| Q75ME0 | 0.8760 | | Q75ME0 | STX1A {ECO:0000313\|EMBL:AAS07469.1} |
| D0PNI1 | 0.8760 | | D0PNI1 | Epididymis luminal protein 4 |
| ACTS | 0.8760 | | P68133 | Actin, alpha skeletal muscle precursor |
| 1433Z | 0.8760 | | P63104 | 14-3-3 protein zeta/delta |
| SYUA | 0.8747 | | P37840 | Alpha-synuclein |
| SMAD2 | 0.8747 | | Q15796 | Mothers against decapentaplegic homolog 2 |
| SAT1 | 0.8747 | | P21673 | Diamine acetyltransferase 1 |
| RHOA | 0.8747 | | P61586 | Transforming protein RhoA precursor |
| Q6ICU9 | 0.8747 | | Q6ICU9 | SAT {ECO:0000313\|EMBL:CAG29290.1} |
| H0YLN8 | 0.8747 | | H0YLN8 | TRPM7 {ECO:0000313\|EMBL:EAW77407.1, |
| FYN | 0.8747 | | P06241 | Tyrosine-protein kinase Fyn |
| V9HW88 | 0.8733 | | V9HW88 | HEL-S-99n {ECO:0000313\|EMBL:ACI46003.1} |
| SUMO1 | 0.8733 | | P63165 | Small ubiquitin-related modifier 1 precursor |
| PRAF1 | 0.8733 | | Q9UI14 | Prenylated Rab acceptor protein 1 |
| NEDD8 | 0.8733 | | Q15843 | NEDD8 precursor |
| HMGB1 | 0.8733 | | P09429 | High mobility group protein B1 |
| CALR | 0.8733 | | P27797 | Calreticulin precursor |
| ACTC | 0.8720 | | P68032 | Actin, alpha cardiac muscle 1 precursor |
| SMAD9 | 0.8707 | | O15198 | Mothers against decapentaplegic homolog 9 |
| N0E4C7 | 0.8707 | | N0E4C7 | CSNK2B {ECO:0000313\|EMBL:CCI79683.1} |
| MDFI | 0.8707 | | Q99750 | MyoD family inhibitor |
| CSK2B | 0.8707 | | P67870 | Casein kinase II subunit beta |
| Q38L21 | 0.8693 | | Q38L21 | CCR5 {ECO:0000313\|EMBL:ABB01004.1} |
| CTNB1 | 0.8693 | | P35222 | Catenin beta-1 |
| CCR5 | 0.8693 | | P51681 | C-C chemokine receptor type 5 |
| V9HWD6 | 0.8680 | | V9HWD6 | HEL-S-1 {ECO:0000313\|EMBL:ACJ13634.1} |
| MYC | 0.8680 | | P01106 | Myc proto-oncogene protein |
| KAPCA | 0.8680 | | P17612 | cAMP-dependent protein kinase catalytic subunit alpha |
| ACTB | 0.8680 | | P60709 | Actin, cytoplasmic 1 |
| 1433B | 0.8680 | | P31946 | 14-3-3 protein beta/alpha |
| RB | 0.8653 | | P06400 | Retinoblastoma-associated protein |
| GFI1B | 0.8653 | | Q5VTD9 | Zinc finger protein Gfi-1b |
| 1433G | 0.8653 | | P61981 | 14-3-3 protein gamma |
| RGS2 | 0.8627 | | P41220 | Regulator of G-protein signaling 2 |
| H2A1 | 0.8627 | | P0C0S8 | Histone H2A type 1 |
| ACTG | 0.8627 | | P63261 | Actin, cytoplasmic 2 |
| A8K7B7 | 0.8627 | | A8K7B7 | PPP2R1A {ECO:0000313\|EMBL:EAW72066.1} |
| 2AAA | 0.8627 | | P30153 | Serine/threonine-protein phosphatase 2A 65 kDa regulatory subunit A alpha isoform |
| ZBT16 | 0.8613 | | Q05516 | Zinc finger and BTB domain-containing protein 16 |
| G4XH65 | 0.8600 | | G4XH65 | ESR1 {ECO:0000313\|EMBL:AEP43755.1} |
| ESR1 | 0.8600 | | P03372 | Estrogen receptor |
| VAV | 0.8587 | | P15498 | Proto-oncogene vav |
| PROF1 | 0.8587 | | P07737 | Profilin-1 |
| H2B1K | 0.8587 | | O60814 | Histone H2B type 1-K |
| UB2D2 | 0.8573 | | P62837 | Ubiquitin-conjugating enzyme E2 D2 |
| SRC | 0.8573 | | P12931 | Proto-oncogene tyrosine-protein kinase Src |
| Q5PY61 | 0.8573 | | Q5PY61 | UBC {ECO:0000313\|EMBL:AAV68344.1, |
| MED31 | 0.8573 | | Q9Y3C7 | Mediator of RNA polymerase II transcription subunit 31 |
| H2B1J | 0.8573 | | P06899 | Histone H2B type 1-J |
| CHIP | 0.8573 | | Q9UNE7 | E3 ubiquitin-protein ligase CHIP |
| ARF1 | 0.8573 | | P84077 | ADP-ribosylation factor 1 |
| UB2D3 | 0.8560 | | P61077 | Ubiquitin-conjugating enzyme E2 D3 |
| RACK1 | 0.8560 | | P63244 | Receptor of activated protein C kinase 1 |
| Q59FK4 | 0.8560 | | Q59FK4 | Tyrosine-protein kinase |
| G5E9I4 | 0.8560 | | G5E9I4 | Breast cancer metastasis suppressor 1, isoform CRA_c |
| TBP | 0.8547 | | P20226 | TATA-box-binding protein |
| CC85B | 0.8547 | | Q15834 | Coiled-coil domain-containing protein 85B |
| PLS1 | 0.8533 | | O15162 | Phospholipid scramblase 1 |
| CSK21 | 0.8533 | | P68400 | Casein kinase II subunit alpha |
| CBP | 0.8533 | | Q92793 | CREB-binding protein |
| MK08 | 0.8520 | | P45983 | Mitogen-activated protein kinase 8 |
| H2A3 | 0.8520 | | Q7L7L0 | Histone H2A type 3 |
| TNFL6 | 0.8507 | | P48023 | Tumor necrosis factor ligand superfamily member 6 |
| RAF1 | 0.8507 | | P04049 | RAF proto-oncogene serine/threonine-protein kinase |
| Q53ZZ1 | 0.8507 | | Q53ZZ1 | TNFSF6 {ECO:0000313\|EMBL:AAO43991.1} |
| Q1HBJ4 | 0.8507 | | Q1HBJ4 | Mitogen-activated protein kinase |
| MK01 | 0.8507 | | P28482 | Mitogen-activated protein kinase 1 |
| L7RSM2 | 0.8507 | | L7RSM2 | MAPK14 {ECO:0000313\|EMBL:AGC09599.1} |
| L7RRS6 | 0.8507 | | L7RRS6 | RAF1 {ECO:0000313\|EMBL:AGC09606.1} |
| HMGA1 | 0.8507 | | P17096 | High mobility group protein HMG-I/HMG-Y |
| FHL3 | 0.8507 | | Q13643 | Four and a half LIM domains protein 3 |
| TRAF6 | 0.8493 | | Q9Y4K3 | TNF receptor-associated factor 6 |
| Q6IPS9 | 0.8493 | | Q6IPS9 | Elongation factor 1-alpha |
| Q6IAW3 | 0.8493 | | Q6IAW3 | CDK5 protein |
| Q08AJ9 | 0.8493 | | Q08AJ9 | HIST1H2AB {ECO:0000313\|EMBL:AAI25141.1} |
| H2A1B | 0.8493 | | P04908 | Histone H2A type 1-B/E |
| EZRI | 0.8493 | | P15311 | Ezrin |
| EP300 | 0.8493 | | Q09472 | Histone acetyltransferase p300 |
| EF1A1 | 0.8493 | | P68104 | Elongation factor 1-alpha 1 |
| CDK5 | 0.8493 | | Q00535 | Cyclin-dependent-like kinase 5 |
| ATN1 | 0.8493 | | P54259 | Atrophin-1 |
| ARF6 | 0.8493 | | P62330 | ADP-ribosylation factor 6 |
| YBOX1 | 0.8480 | | P67809 | Nuclease-sensitive element-binding protein 1 |
| SMAD3 | 0.8480 | | P84022 | Mothers against decapentaplegic homolog 3 |
| Q59E85 | 0.8480 | | Q59E85 | Caveolin |
| Q2TNI1 | 0.8480 | | Q2TNI1 | Caveolin |
| CD9 | 0.8480 | | P21926 | CD9 antigen |
| CAV1 | 0.8480 | | Q03135 | Caveolin-1 |
| H2B1B | 0.8467 | | P33778 | Histone H2B type 1-B |
| BCL2 | 0.8467 | | P10415 | Apoptosis regulator Bcl-2 |
| MLP3A | 0.8453 | | Q9H492 | Microtubule-associated proteins 1A/1B light chain 3A precursor |
| CASP1 | 0.8453 | | P29466 | Caspase-1 precursor |
| TR10D | 0.8440 | | Q9UBN6 | Tumor necrosis factor receptor superfamily member 10D precursor |
| TF65 | 0.8440 | | Q04206 | Transcription factor p65 |
| SMAD7 | 0.8440 | | O15105 | Mothers against decapentaplegic homolog 7 |
| RAC3 | 0.8440 | | P60763 | Ras-related C3 botulinum toxin substrate 3 precursor |
| RAB1A | 0.8440 | | P62820 | Ras-related protein Rab-1A |
| Q5U0I6 | 0.8440 | | Q5U0I6 | RAB1A {ECO:0000313\|EMBL:CAG38727.1} |
| PLK1 | 0.8440 | | P53350 | Serine/threonine-protein kinase PLK1 |
| IKKE | 0.8440 | | Q14164 | Inhibitor of nuclear factor kappa-B kinase subunit epsilon |
| GA45G | 0.8440 | | O95257 | Growth arrest and DNA damage-inducible protein GADD45 gamma |
| TBB2A | 0.8427 | | Q13885 | Tubulin beta-2A chain |
| TAF9 | 0.8427 | | Q16594 | Transcription initiation factor TFIID subunit 9 |
| RNF11 | 0.8427 | | Q9Y3C5 | RING finger protein 11 |
| Q6FG85 | 0.8427 | | Q6FG85 | GC20 {ECO:0000313\|EMBL:CAG47019.1} |
| INS | 0.8427 | | P01308 | Insulin precursor |
| I3WAC9 | 0.8427 | | I3WAC9 | INS {ECO:0000313\|EMBL:AFK93533.1} |
| HD | 0.8427 | | P42858 | Huntingtin |
| EIF1B | 0.8427 | | O60739 | Eukaryotic translation initiation factor 1b |
| TRAF2 | 0.8413 | | Q12933 | TNF receptor-associated factor 2 |
| SKP1 | 0.8413 | | P63208 | S-phase kinase-associated protein 1 |
| NUCL | 0.8413 | | P19338 | Nucleolin |
| NHRF1 | 0.8413 | | O14745 | Na(+)/H(+) exchange regulatory cofactor NHE-RF1 |
| MDM2 | 0.8413 | | Q00987 | E3 ubiquitin-protein ligase Mdm2 |
| MAGAB | 0.8413 | | P43364 | Melanoma-associated antigen 11 |
| KSYK | 0.8413 | | P43405 | Tyrosine-protein kinase SYK |
| IKKA | 0.8413 | | O15111 | Inhibitor of nuclear factor kappa-B kinase subunit alpha |
| V9HW41 | 0.8400 | | V9HW41 | HEL-S-71 {ECO:0000313\|EMBL:ACI46046.1} |
| UBE2N | 0.8400 | | P61088 | Ubiquitin-conjugating enzyme E2 N |
| Q7LCB3 | 0.8400 | | Q7LCB3 | 5p152 |
| HS71B | 0.8400 | | P0DMV9 | Heat shock 70 kDa protein 1B |
| HS71A | 0.8400 | | P0DMV8 | Heat shock 70 kDa protein 1A |
| H7C2I1 | 0.8400 | | H7C2I1 | PRMT1 {ECO:0000313\|EMBL:EAW52519.1, |
| ESR2 | 0.8400 | | Q92731 | Estrogen receptor beta |
| CO2A1 | 0.8400 | | P02458 | Collagen alpha-1(II) chain precursor |
| CDK9 | 0.8400 | | P50750 | Cyclin-dependent kinase 9 |
| A8K5I0 | 0.8400 | | A8K5I0 | Epididymis secretory protein Li 103 |
| 1433T | 0.8400 | | P27348 | 14-3-3 protein theta |
| UB2D4 | 0.8387 | | Q9Y2X8 | Ubiquitin-conjugating enzyme E2 D4 |
| LRIF1 | 0.8387 | | Q5T3J3 | Ligand-dependent nuclear receptor-interacting factor 1 |
| LMO3 | 0.8387 | | Q8TAP4 | LIM domain only protein 3 |
| HDAC3 | 0.8387 | | O15379 | Histone deacetylase 3 |
| UB2V2 | 0.8373 | | Q15819 | Ubiquitin-conjugating enzyme E2 variant 2 |
| TBB3 | 0.8373 | | Q13509 | Tubulin beta-3 chain |
| S10A4 | 0.8373 | | P26447 | Protein S100-A4 |
| NPM | 0.8373 | | P06748 | Nucleophosmin |
| NMDZ1 | 0.8373 | | Q05586 | Glutamate receptor ionotropic, NMDA 1 precursor |
| NDRG1 | 0.8373 | | Q92597 | Protein NDRG1 |
| LNX1 | 0.8373 | | Q8TBB1 | E3 ubiquitin-protein ligase LNX |
| A4 | 0.8373 | | P05067 | Amyloid beta A4 protein precursor |
| A0M8W4 | 0.8373 | | A0M8W4 | Ubiquitin-conjugating enzyme E2 variant 2 |
| SRC8 | 0.8360 | | Q14247 | Src substrate cortactin |
| Q53SS8 | 0.8360 | | Q53SS8 | PCBP1 {ECO:0000313\|EMBL:AAX93191.1} |
| PKHF2 | 0.8360 | | Q9H8W4 | Pleckstrin homology domain-containing family F member 2 |
| PCBP1 | 0.8360 | | Q15365 | Poly(rC)-binding protein 1 |
| MK | 0.8360 | | P21741 | Midkine precursor |
| H2A1D | 0.8360 | | P20671 | Histone H2A type 1-D |
| GNAI1 | 0.8360 | | P63096 | Guanine nucleotide-binding protein G(i) subunit alpha-1 |
| B2CL1 | 0.8360 | | Q07817 | Bcl-2-like protein 1 |
| TGFR1 | 0.8347 | | P36897 | TGF-beta receptor type-1 precursor |
| Q5T7S2 | 0.8347 | | Q5T7S2 | TGFBR1 {ECO:0000313\|EMBL:ACZ58375.1} |
| D3DPA4 | 0.8347 | | D3DPA4 | ACVR1 {ECO:0000313\|EMBL:EAX11440.1} |
| CD44 | 0.8347 | | P16070 | CD44 antigen precursor |
| ACVR1 | 0.8347 | | Q04771 | Activin receptor type-1 precursor |
| 1433S | 0.8347 | | P31947 | 14-3-3 protein sigma |
| Q9BSD8 | 0.8333 | | Q9BSD8 | Similar to E2F transcription factor 1 |
| Q6FG41 | 0.8333 | | Q6FG41 | FOS {ECO:0000313\|EMBL:CAG47063.1} |
| FOS | 0.8333 | | P01100 | Proto-oncogene c-Fos |
| E2F1 | 0.8333 | | Q01094 | Transcription factor E2F1 |
| AP2A | 0.8333 | | P05549 | Transcription factor AP-2-alpha |
| TOM20 | 0.8320 | | Q15388 | Mitochondrial import receptor subunit TOM20 homolog |
| PHOCN | 0.8320 | | Q9Y3A3 | MOB-like protein phocein |
| PAK2 | 0.8320 | | Q13177 | Serine/threonine-protein kinase PAK 2 |
| H2A1C | 0.8320 | | Q93077 | Histone H2A type 1-C |
| GRAB | 0.8320 | | P10144 | Granzyme B precursor |
| GASP1 | 0.8320 | | Q5JY77 | G-protein coupled receptor-associated sorting protein 1 |
| CENPA | 0.8320 | | P49450 | Histone H3-like centromeric protein A |
| 1B42 | 0.8320 | | P30480 | HLA class I histocompatibility antigen, B-42 alpha chain precursor |
| PP2AA | 0.8307 | | P67775 | Serine/threonine-protein phosphatase 2A catalytic subunit alpha isoform |
| PIN1 | 0.8307 | | Q13526 | Peptidyl-prolyl cis-trans isomerase NIMA-interacting 1 |
| VIME | 0.8293 | | P08670 | Vimentin |
| V9HWE1 | 0.8293 | | V9HWE1 | HEL113 {ECO:0000313\|EMBL:ACJ13726.1} |
| V9H0H7 | 0.8293 | | V9H0H7 | HSPC022 |
| TP4A3 | 0.8293 | | O75365 | Protein tyrosine phosphatase type IVA 3 precursor |
| STAT3 | 0.8293 | | P40763 | Signal transducer and activator of transcription 3 |
| RAC2 | 0.8293 | | P15153 | Ras-related C3 botulinum toxin substrate 2 precursor |
| MK03 | 0.8293 | | P27361 | Mitogen-activated protein kinase 3 |
| IMA1 | 0.8293 | | P52292 | Importin subunit alpha-1 |
| SMAD1 | 0.8280 | | Q15797 | Mothers against decapentaplegic homolog 1 |
| PSN1 | 0.8280 | | P49768 | Presenilin-1 |
| KITH | 0.8280 | | P04183 | Thymidine kinase, cytosolic |
| B4E1C1 | 0.8280 | | B4E1C1 | cDNA FLJ55943 |
| AAKB1 | 0.8280 | | Q9Y478 | 5&#039;-AMP-activated protein kinase subunit beta-1 |
| SKIL | 0.8267 | | P12757 | Ski-like protein |
| SET | 0.8267 | | Q01105 | Protein SET |
| Q5VXV3 | 0.8267 | | Q5VXV3 | SET {ECO:0000313\|EMBL:EAW87818.1} |
| Q2TAZ1 | 0.8267 | | Q2TAZ1 | CHD3 protein |
| MTR1B | 0.8267 | | P49286 | Melatonin receptor type 1B |
| FAK2 | 0.8267 | | Q14289 | Protein-tyrosine kinase 2-beta |
| D2JYH4 | 0.8267 | | D2JYH4 | ACTA2 {ECO:0000313\|EMBL:ACZ58370.1} |
| CDK8 | 0.8267 | | P49336 | Cyclin-dependent kinase 8 |
| CBL | 0.8267 | | P22681 | E3 ubiquitin-protein ligase CBL |
| ACTA | 0.8267 | | P62736 | Actin, aortic smooth muscle precursor |
| THIO | 0.8253 | | P10599 | Thioredoxin |
| SRSF2 | 0.8253 | | Q01130 | Serine/arginine-rich splicing factor 2 |
| MAX | 0.8253 | | P61244 | Protein max |
| JAK2 | 0.8253 | | O60674 | Tyrosine-protein kinase JAK2 |
| H9ZYJ2 | 0.8253 | | H9ZYJ2 | TXN {ECO:0000313\|EMBL:AFH41799.1} |
| GTR4 | 0.8253 | | P14672 | Solute carrier family 2, facilitated glucose transporter member 4 |
| GNAI2 | 0.8253 | | P04899 | Guanine nucleotide-binding protein G(i) subunit alpha-2 |
| ZN250 | 0.8240 | | P15622 | Zinc finger protein 250 |
| V9HWB4 | 0.8240 | | V9HWB4 | HEL-S-89n {ECO:0000313\|EMBL:ACJ13671.1} |
| NUPR1 | 0.8240 | | O60356 | Nuclear protein 1 |
| H2B1N | 0.8240 | | Q99877 | Histone H2B type 1-N |
| GRP78 | 0.8240 | | P11021 | 78 kDa glucose-regulated protein precursor |
| FEZ1 | 0.8240 | | Q99689 | Fasciculation and elongation protein zeta-1 |
| CTBP1 | 0.8240 | | Q13363 | C-terminal-binding protein 1 |
| Q6NXF2 | 0.8227 | | Q6NXF2 | FLNA protein |
| FLNA | 0.8227 | | P21333 | Filamin-A |
| E5KQF6 | 0.8227 | | E5KQF6 | NR3C1 {ECO:0000313\|EMBL:EAW61870.1} |
| TBB4B | 0.8213 | | P68371 | Tubulin beta-4B chain |
| KAP0 | 0.8213 | | P10644 | cAMP-dependent protein kinase type I-alpha regulatory subunit |
| CAN1 | 0.8213 | | P07384 | Calpain-1 catalytic subunit |
| B2R5T5 | 0.8213 | | B2R5T5 | PRKAR1A {ECO:0000313\|EMBL:EAW89060.1} |
| Q59FY1 | 0.8200 | | Q59FY1 | Synapse-associated protein 102 variant |
| HAP1 | 0.8200 | | P54257 | Huntingtin-associated protein 1 |
| DLG3 | 0.8200 | | Q92796 | Disks large homolog 3 |
| CIB1 | 0.8200 | | Q99828 | Calcium and integrin-binding protein 1 |
| CD28 | 0.8200 | | P10747 | T-cell-specific surface glycoprotein CD28 precursor |
| ZNF24 | 0.8187 | | P17028 | Zinc finger protein 24 |
| NCOA1 | 0.8187 | | Q15788 | Nuclear receptor coactivator 1 |
| LCK | 0.8187 | | P06239 | Tyrosine-protein kinase Lck |
| H2B1H | 0.8187 | | Q93079 | Histone H2B type 1-H |
| E2AK2 | 0.8187 | | P19525 | Interferon-induced, double-stranded RNA-activated protein kinase |
| CD5R1 | 0.8187 | | Q15078 | Cyclin-dependent kinase 5 activator 1 precursor |
| CASP4 | 0.8187 | | P49662 | Caspase-4 precursor |
| XPO1 | 0.8173 | | O14980 | Exportin-1 |
| V9HW98 | 0.8173 | | V9HW98 | HEL2 {ECO:0000313\|EMBL:ACJ13635.1} |
| TS101 | 0.8173 | | Q99816 | Tumor susceptibility gene 101 protein |
| TBB5 | 0.8173 | | P07437 | Tubulin beta chain |
| Q5SU16 | 0.8173 | | Q5SU16 | TUBB {ECO:0000313\|EMBL:BAF31267.1} |
| JUN | 0.8173 | | P05412 | Transcription factor AP-1 |
| EGFR | 0.8173 | | P00533 | Epidermal growth factor receptor precursor |
| CSK | 0.8173 | | P41240 | Tyrosine-protein kinase CSK |
| BTK | 0.8173 | | Q06187 | Tyrosine-protein kinase BTK |
| BEX3 | 0.8173 | | Q00994 | Protein BEX3 |
| B2R6Q4 | 0.8173 | | B2R6Q4 | CSK {ECO:0000313\|EMBL:EAW99311.1} |
| 1433E | 0.8173 | | P62258 | 14-3-3 protein epsilon |
| SOS1 | 0.8160 | | Q07889 | Son of sevenless homolog 1 |
| RSMB | 0.8160 | | P14678 | Small nuclear ribonucleoprotein-associated proteins B and B&#039; |
| KPCA | 0.8160 | | P17252 | Protein kinase C alpha type |
| IF4E | 0.8160 | | P06730 | Eukaryotic translation initiation factor 4E |
| CRCT1 | 0.8160 | | Q9UGL9 | Cysteine-rich C-terminal protein 1 |
| ANDR | 0.8160 | | P10275 | Androgen receptor |
| V9HW44 | 0.8147 | | V9HW44 | HEL-S-303 {ECO:0000313\|EMBL:ACI46020.1} |
| TNR14 | 0.8147 | | Q92956 | Tumor necrosis factor receptor superfamily member 14 precursor |
| TBA4A | 0.8147 | | P68366 | Tubulin alpha-4A chain |
| SMAD4 | 0.8147 | | Q13485 | Mothers against decapentaplegic homolog 4 |
| SIN3A | 0.8147 | | Q96ST3 | Paired amphipathic helix protein Sin3a |
| RXRA | 0.8147 | | P19793 | Retinoic acid receptor RXR-alpha |
| Q6P3U7 | 0.8147 | | Q6P3U7 | RXRA protein |
| Q53YD7 | 0.8147 | | Q53YD7 | EEF1G {ECO:0000313\|EMBL:CAG28553.1} |
| PTMA | 0.8147 | | P06454 | Prothymosin alpha |
| PSN2 | 0.8147 | | P49810 | Presenilin-2 |
| PA1B2 | 0.8147 | | P68402 | Platelet-activating factor acetylhydrolase IB subunit beta |
| KPCD | 0.8147 | | Q05655 | Protein kinase C delta type |
| F1D8Q5 | 0.8147 | | F1D8Q5 | NR2B1 {ECO:0000313\|EMBL:ADZ17354.1} |
| EF1G | 0.8147 | | P26641 | Elongation factor 1-gamma |
| D0EI67 | 0.8147 | | D0EI67 | C-C motif chemokine |
| CCL5 | 0.8147 | | P13501 | C-C motif chemokine 5 precursor |
| BCAR1 | 0.8147 | | P56945 | Breast cancer anti-estrogen resistance protein 1 |
| V9HVZ4 | 0.8133 | | V9HVZ4 | HEL-S-162eP {ECO:0000313\|EMBL:ACF94474.1} |
| TFE2 | 0.8133 | | P15923 | Transcription factor E2-alpha |
| SOMA | 0.8133 | | P01241 | Somatotropin precursor |
| RBBP4 | 0.8133 | | Q09028 | Histone-binding protein RBBP4 |
| IRS1 | 0.8133 | | P35568 | Insulin receptor substrate 1 |
| GNAQ | 0.8133 | | P50148 | Guanine nucleotide-binding protein G(q) subunit alpha |
| G3P | 0.8133 | | P04406 | Glyceraldehyde-3-phosphate dehydrogenase |
| B1A4G6 | 0.8133 | | B1A4G6 | GH1 {ECO:0000313\|EMBL:ABZ88711.1} |
| ATM | 0.8133 | | Q13315 | Serine-protein kinase ATM |
| ASCC2 | 0.8133 | | Q9H1I8 | Activating signal cointegrator 1 complex subunit 2 |
| AN32A | 0.8133 | | P39687 | Acidic leucine-rich nuclear phosphoprotein 32 family member A |
| STAT1 | 0.8120 | | P42224 | Signal transducer and activator of transcription 1-alpha/beta |
| CSTF2 | 0.8120 | | P33240 | Cleavage stimulation factor subunit 2 |
| BARD1 | 0.8120 | | Q99728 | BRCA1-associated RING domain protein 1 |
| VGFR2 | 0.8107 | | P35968 | Vascular endothelial growth factor receptor 2 precursor |
| UBE2W | 0.8107 | | Q96B02 | Ubiquitin-conjugating enzyme E2 W |
| Q86V38 | 0.8107 | | Q86V38 | Atrophin 1 |
| PPARG | 0.8107 | | P37231 | Peroxisome proliferator-activated receptor gamma |
| HIPK2 | 0.8107 | | Q9H2X6 | Homeodomain-interacting protein kinase 2 |
| ETS1 | 0.8107 | | P14921 | Protein C-ets-1 |
| ABCA1 | 0.8107 | | O95477 | ATP-binding cassette sub-family A member 1 |
| VAV2 | 0.8093 | | P52735 | Guanine nucleotide exchange factor VAV2 |
| THB | 0.8093 | | P10828 | Thyroid hormone receptor beta |
| RFA2 | 0.8093 | | P15927 | Replication protein A 32 kDa subunit |
| Q9UII7 | 0.8093 | | Q9UII7 | E-cadherin |
| Q6NUK7 | 0.8093 | | Q6NUK7 | Tyrosine-protein kinase |
| Q658W2 | 0.8093 | | Q658W2 | Putative uncharacterized protein DKFZp666O0110 |
| NCOR1 | 0.8093 | | O75376 | Nuclear receptor corepressor 1 |
| LYN | 0.8093 | | P07948 | Tyrosine-protein kinase Lyn |
| GCR | 0.8093 | | P04150 | Glucocorticoid receptor |
| F1D8N4 | 0.8093 | | F1D8N4 | NR3C1 {ECO:0000313\|EMBL:ADZ17333.1} |
| CAV3 | 0.8093 | | P56539 | Caveolin-3 |
| ATPG | 0.8093 | | P36542 | ATP synthase subunit gamma, mitochondrial precursor |
| UBC | 0.8080 | | P0CG48 | Polyubiquitin-C precursor |
| RENT2 | 0.8080 | | Q9HAU5 | Regulator of nonsense transcripts 2 |
| RARA | 0.8080 | | P10276 | Retinoic acid receptor alpha |
| Q6I9R7 | 0.8080 | | Q6I9R7 | RARA {ECO:0000313\|EMBL:CAG33719.1} |
| MP2K1 | 0.8080 | | Q02750 | Dual specificity mitogen-activated protein kinase kinase 1 |
| MCM2 | 0.8080 | | P49736 | DNA replication licensing factor MCM2 |
| IMB1 | 0.8080 | | Q14974 | Importin subunit beta-1 |
| A4QPA9 | 0.8080 | | A4QPA9 | MAP2K1 {ECO:0000313\|EMBL:AAI39730.1} |
| 1433F | 0.8080 | | Q04917 | 14-3-3 protein eta |
| UBQL4 | 0.8067 | | Q9NRR5 | Ubiquilin-4 |
| MRE11 | 0.8067 | | P49959 | Double-strand break repair protein MRE11A |
| ITK | 0.8067 | | Q08881 | Tyrosine-protein kinase ITK/TSK |
| CFLAR | 0.8067 | | O15519 | CASP8 and FADD-like apoptosis regulator precursor |
| V9HW22 | 0.8053 | | V9HW22 | HEL-S-72p {ECO:0000313\|EMBL:ACI45986.1} |
| Q9HBD4 | 0.8053 | | Q9HBD4 | SMARCA4 isoform 2 |
| Q6FII1 | 0.8053 | | Q6FII1 | Glutathione S-transferase kappa 1 |
| Q59FC3 | 0.8053 | | Q59FC3 | G protein-coupled receptor kinase interactor 1 variant |
| PABP1 | 0.8053 | | P11940 | Polyadenylate-binding protein 1 |
| KAP2 | 0.8053 | | P13861 | cAMP-dependent protein kinase type II-alpha regulatory subunit |
| HSP7C | 0.8053 | | P11142 | Heat shock cognate 71 kDa protein |
| GSTK1 | 0.8053 | | Q9Y2Q3 | Glutathione S-transferase kappa 1 |
| CRKL | 0.8053 | | P46109 | Crk-like protein |
| SP1 | 0.8040 | | P08047 | Transcription factor Sp1 |
| SMUF2 | 0.8040 | | Q9HAU4 | E3 ubiquitin-protein ligase SMURF2 |
| SKP2 | 0.8040 | | Q13309 | S-phase kinase-associated protein 2 |
| Q8N6N5 | 0.8040 | | Q8N6N5 | TUBB2C {ECO:0000313\|EMBL:AAH29529.1} |
| MK12 | 0.8040 | | P53778 | Mitogen-activated protein kinase 12 |
| H2B1D | 0.8040 | | P58876 | Histone H2B type 1-D |
| BMPR2 | 0.8040 | | Q13873 | Bone morphogenetic protein receptor type-2 precursor |
| XRCC6 | 0.8027 | | P12956 | X-ray repair cross-complementing protein 6 |
| UB2V1 | 0.8027 | | Q13404 | Ubiquitin-conjugating enzyme E2 variant 1 |
| RHOG | 0.8027 | | P84095 | Rho-related GTP-binding protein RhoG precursor |
| Q6ICQ8 | 0.8027 | | Q6ICQ8 | ARHG {ECO:0000313\|EMBL:CAG29331.1} |
| NFKB2 | 0.8027 | | Q00653 | Nuclear factor NF-kappa-B p100 subunit |
| KIT | 0.8027 | | P10721 | Mast/stem cell growth factor receptor Kit precursor |
| CO1A1 | 0.8027 | | P02452 | Collagen alpha-1(I) chain precursor |
| V9HWP2 | 0.8013 | | V9HWP2 | HEL-S-125m {ECO:0000313\|EMBL:ACS44652.1} |
| SMAD5 | 0.8013 | | Q99717 | Mothers against decapentaplegic homolog 5 |
| RGS12 | 0.8013 | | O14924 | Regulator of G-protein signaling 12 |
| RAP2A | 0.8013 | | P10114 | Ras-related protein Rap-2a precursor |
| RAN | 0.8013 | | P62826 | GTP-binding nuclear protein Ran |
| Q68DB7 | 0.8013 | | Q68DB7 | Mothers against decapentaplegic homolog |
| PCNA | 0.8013 | | P12004 | Proliferating cell nuclear antigen |
| ENPL | 0.8013 | | P14625 | Endoplasmin precursor |
| SETB1 | 0.8000 | | Q15047 | Histone-lysine N-methyltransferase SETDB1 |
| RHG01 | 0.8000 | | Q07960 | Rho GTPase-activating protein 1 |
| PHS | 0.8000 | | P61457 | Pterin-4-alpha-carbinolamine dehydratase |
| PHB | 0.8000 | | P35232 | Prohibitin |
| NCF1 | 0.8000 | | P14598 | Neutrophil cytosol factor 1 |
| HS90A | 0.8000 | | P07900 | Heat shock protein HSP 90-alpha |
| EF1D | 0.8000 | | P29692 | Elongation factor 1-delta |
| ANM1 | 0.8000 | | Q99873 | Protein arginine N-methyltransferase 1 |
| A8K401 | 0.8000 | | A8K401 | PHB {ECO:0000313\|EMBL:EAW94680.1} |
| TTHY | 0.7987 | | P02766 | Transthyretin precursor |
| TRI27 | 0.7987 | | P14373 | Zinc finger protein RFP |
| MERL | 0.7987 | | P35240 | Merlin |
| LIMK1 | 0.7987 | | P53667 | LIM domain kinase 1 |
| KHDR1 | 0.7987 | | Q07666 | KH domain-containing, RNA-binding, signal transduction-associated protein 1 |
| H2B1O | 0.7987 | | P23527 | Histone H2B type 1-O |
| FBLN4 | 0.7987 | | O95967 | EGF-containing fibulin-like extracellular matrix protein 2 precursor |
| ERBB2 | 0.7987 | | P04626 | Receptor tyrosine-protein kinase erbB-2 precursor |
| EPS15 | 0.7987 | | P42566 | Epidermal growth factor receptor substrate 15 |
| E9KL36 | 0.7987 | | E9KL36 | HEL111 {ECO:0000313\|EMBL:ACJ13724.1} |
| CDK4 | 0.7987 | | P11802 | Cyclin-dependent kinase 4 |
| B2R5B6 | 0.7987 | | B2R5B6 | . |
| ZFYV9 | 0.7973 | | O95405 | Zinc finger FYVE domain-containing protein 9 |
| VDAC1 | 0.7973 | | P21796 | Voltage-dependent anion-selective channel protein 1 |
| V9HW27 | 0.7973 | | V9HW27 | HEL-S-101 {ECO:0000313\|EMBL:ACI46006.1} |
| SPB9 | 0.7973 | | P50453 | Serpin B9 |
| SNAA | 0.7973 | | P54920 | Alpha-soluble NSF attachment protein |
| Q9UFQ0 | 0.7973 | | Q9UFQ0 | Putative uncharacterized protein DKFZp434K0435 |
| Q32MN7 | 0.7973 | | Q32MN7 | TBP {ECO:0000313\|EMBL:AAI09054.1} |
| NCOR2 | 0.7973 | | Q9Y618 | Nuclear receptor corepressor 2 |
| MCAF1 | 0.7973 | | Q6VMQ6 | Activating transcription factor 7-interacting protein 1 |
| H2A2A | 0.7973 | | Q6FI13 | Histone H2A type 2-A |
| CRYAB | 0.7973 | | P02511 | Alpha-crystallin B chain |
| CO4A2 | 0.7973 | | P08572 | Collagen alpha-2(IV) chain precursor |
| CEBPB | 0.7973 | | P17676 | CCAAT/enhancer-binding protein beta |
| TOPB1 | 0.7960 | | Q92547 | DNA topoisomerase 2-binding protein 1 |
| S10A1 | 0.7960 | | P23297 | Protein S100-A1 |
| RENT1 | 0.7960 | | Q92900 | Regulator of nonsense transcripts 1 |
| RASF1 | 0.7960 | | Q9NS23 | Ras association domain-containing protein 1 |
| PAIRB | 0.7960 | | Q8NC51 | Plasminogen activator inhibitor 1 RNA-binding protein |
| JAK1 | 0.7960 | | P23458 | Tyrosine-protein kinase JAK1 |
| DLGP1 | 0.7960 | | O14490 | Disks large-associated protein 1 |
| ARL3 | 0.7960 | | P36405 | ADP-ribosylation factor-like protein 3 |
| ADT1 | 0.7960 | | P12235 | ADP/ATP translocase 1 |
| 41 | 0.7960 | | P11171 | Protein 4.1 |
| TBB4A | 0.7947 | | P04350 | Tubulin beta-4A chain |
| RUVB1 | 0.7947 | | Q9Y265 | RuvB-like 1 |
| RAB4A | 0.7947 | | P20338 | Ras-related protein Rab-4A |
| PP2AB | 0.7947 | | P62714 | Serine/threonine-protein phosphatase 2A catalytic subunit beta isoform |
| PLMN | 0.7947 | | P00747 | Plasminogen precursor |
| IBP5 | 0.7947 | | P24593 | Insulin-like growth factor-binding protein 5 precursor |
| HS90B | 0.7947 | | P08238 | Heat shock protein HSP 90-beta |
| HOME1 | 0.7947 | | Q86YM7 | Homer protein homolog 1 |
| ERBB4 | 0.7947 | | Q15303 | Receptor tyrosine-protein kinase erbB-4 precursor |
| D2JYI1 | 0.7947 | | D2JYI1 | TGFBR2 {ECO:0000313\|EMBL:ACZ58377.1} |
| CUL1 | 0.7947 | | Q13616 | Cullin-1 |
| BRCA2 | 0.7947 | | P51587 | Breast cancer type 2 susceptibility protein |
| Q5U045 | 0.7933 | | Q5U045 | CSNK1E {ECO:0000313\|EMBL:ABM64212.1} |
| LCP2 | 0.7933 | | Q13094 | Lymphocyte cytosolic protein 2 |
| KC1E | 0.7933 | | P49674 | Casein kinase I isoform epsilon |
| VASP | 0.7920 | | P50552 | Vasodilator-stimulated phosphoprotein |
| VAMP3 | 0.7920 | | Q15836 | Vesicle-associated membrane protein 3 |
| V9HW04 | 0.7920 | | V9HW04 | HEL-S-80p {ECO:0000313\|EMBL:ACF94492.1} |
| TRI63 | 0.7920 | | Q969Q1 | E3 ubiquitin-protein ligase TRIM63 |
| TM1L1 | 0.7920 | | O75674 | TOM1-like protein 1 |
| TERF1 | 0.7920 | | P54274 | Telomeric repeat-binding factor 1 |
| RHG32 | 0.7920 | | A7KAX9 | Rho GTPase-activating protein 32 |
| Q6FGG2 | 0.7920 | | Q6FGG2 | VAMP3 protein |
| PP1B | 0.7920 | | P62140 | Serine/threonine-protein phosphatase PP1-beta catalytic subunit |
| PML | 0.7920 | | P29590 | Protein PML |
| ID3 | 0.7920 | | Q02535 | DNA-binding protein inhibitor ID-3 |
| H2B3B | 0.7920 | | Q8N257 | Histone H2B type 3-B |
| CC90B | 0.7920 | | Q9GZT6 | Coiled-coil domain-containing protein 90B, mitochondrial precursor |
| CACO2 | 0.7920 | | Q13137 | Calcium-binding and coiled-coil domain-containing protein 2 |
| TNR1A | 0.7907 | | P19438 | Tumor necrosis factor receptor superfamily member 1A precursor |
| SRSF3 | 0.7907 | | P84103 | Serine/arginine-rich splicing factor 3 |
| PCH2 | 0.7907 | | Q15645 | Pachytene checkpoint protein 2 homolog |
| MEG10 | 0.7907 | | Q96KG7 | Multiple epidermal growth factor-like domains protein 10 precursor |
| KPCT | 0.7907 | | Q04759 | Protein kinase C theta type |
| IKKB | 0.7907 | | O14920 | Inhibitor of nuclear factor kappa-B kinase subunit beta |
| CADH2 | 0.7907 | | P19022 | Cadherin-2 precursor |
| B2R6F3 | 0.7907 | | B2R6F3 | SFRS3 {ECO:0000313\|EMBL:BAG70043.1} |
| UBP7 | 0.7893 | | Q93009 | Ubiquitin carboxyl-terminal hydrolase 7 |
| UB2D1 | 0.7893 | | P51668 | Ubiquitin-conjugating enzyme E2 D1 |
| TOP2A | 0.7893 | | P11388 | DNA topoisomerase 2-alpha |
| SPOP | 0.7893 | | O43791 | Speckle-type POZ protein |
| RAB5A | 0.7893 | | P20339 | Ras-related protein Rab-5A |
| Q8IWE0 | 0.7893 | | Q8IWE0 | Calcium/calmodulin-dependent protein kinase II alpha |
| Q6U8A4 | 0.7893 | | Q6U8A4 | Ubiquitin-specific protease 7 isoform |
| NOTC1 | 0.7893 | | P46531 | Neurogenic locus notch homolog protein 1 precursor |
| MBP | 0.7893 | | P02686 | Myelin basic protein |
| M3K3 | 0.7893 | | Q99759 | Mitogen-activated protein kinase kinase kinase 3 |
| LEU1 | 0.7893 | | O43261 | Leukemia-associated protein 1 |
| FGFR1 | 0.7893 | | P11362 | Fibroblast growth factor receptor 1 precursor |
| EPOR | 0.7893 | | P19235 | Erythropoietin receptor precursor |
| ATX2 | 0.7893 | | Q99700 | Ataxin-2 |
| A8K161 | 0.7893 | | A8K161 | CAMK2A {ECO:0000313\|EMBL:EAW61742.1} |
| WASP | 0.7880 | | P42768 | Wiskott-Aldrich syndrome protein |
| TAF7 | 0.7880 | | Q15545 | Transcription initiation factor TFIID subunit 7 |
| S10AA | 0.7880 | | P60903 | Protein S100-A10 |
| RASK | 0.7880 | | P01116 | GTPase KRas precursor |
| RAB8A | 0.7880 | | P61006 | Ras-related protein Rab-8A precursor |
| NCOA6 | 0.7880 | | Q14686 | Nuclear receptor coactivator 6 |
| MT2 | 0.7880 | | P02795 | Metallothionein-2 |
| L7RSL8 | 0.7880 | | L7RSL8 | KRAS {ECO:0000313\|EMBL:AGC09594.1} |
| CO1A2 | 0.7880 | | P08123 | Collagen alpha-2(I) chain precursor |
| CCNA1 | 0.7880 | | P78396 | Cyclin-A1 |
| UB2R1 | 0.7867 | | P49427 | Ubiquitin-conjugating enzyme E2 R1 |
| PSA7 | 0.7867 | | O14818 | Proteasome subunit alpha type-7 |
| P73 | 0.7867 | | O15350 | Tumor protein p73 |
| OCLN | 0.7867 | | Q16625 | Occludin |
| MK09 | 0.7867 | | P45984 | Mitogen-activated protein kinase 9 |
| HOOK2 | 0.7867 | | Q96ED9 | Protein Hook homolog 2 |
| 2A5A | 0.7867 | | Q15172 | Serine/threonine-protein phosphatase 2A 56 kDa regulatory subunit alpha isoform |
| VHL | 0.7853 | | P40337 | Von Hippel-Lindau disease tumor suppressor |
| Q6I9V5 | 0.7853 | | Q6I9V5 | SLC25A6 protein |
| NRIP1 | 0.7853 | | P48552 | Nuclear receptor-interacting protein 1 |
| MMP2 | 0.7853 | | P08253 | 72 kDa type IV collagenase precursor |
| KNG1 | 0.7853 | | P01042 | Kininogen-1 precursor |
| K2C8 | 0.7853 | | P05787 | Keratin, type II cytoskeletal 8 |
| APBP2 | 0.7853 | | Q92624 | Amyloid protein-binding protein 2 |
| ADT3 | 0.7853 | | P12236 | ADP/ATP translocase 3 |
| A8K3H8 | 0.7853 | | A8K3H8 | . |
| V9HW80 | 0.7840 | | V9HW80 | HEL-S-70 {ECO:0000313\|EMBL:ACI46044.1} |
| TNNT1 | 0.7840 | | P13805 | Troponin T, slow skeletal muscle |
| TERA | 0.7840 | | P55072 | Transitional endoplasmic reticulum ATPase |
| S10A8 | 0.7840 | | P05109 | Protein S100-A8 |
| RRAS2 | 0.7840 | | P62070 | Ras-related protein R-Ras2 precursor |
| Q59HG2 | 0.7840 | | Q59HG2 | TEK tyrosine kinase variant |
| Q0VDC6 | 0.7840 | | Q0VDC6 | Peptidylprolyl isomerase |
| PR15A | 0.7840 | | O75807 | Protein phosphatase 1 regulatory subunit 15A |
| M3K7 | 0.7840 | | O43318 | Mitogen-activated protein kinase kinase kinase 7 |
| HSP74 | 0.7840 | | P34932 | Heat shock 70 kDa protein 4 |
| FKB1A | 0.7840 | | P62942 | Peptidyl-prolyl cis-trans isomerase FKBP1A |
| DAB2 | 0.7840 | | P98082 | Disabled homolog 2 |
| CLH1 | 0.7840 | | Q00610 | Clathrin heavy chain 1 |
| CHK1 | 0.7840 | | O14757 | Serine/threonine-protein kinase Chk1 |
| V9HWI5 | 0.7827 | | V9HWI5 | HEL-S-15 {ECO:0000313\|EMBL:ACJ13657.1} |
| TBA3C | 0.7827 | | Q13748 | Tubulin alpha-3C/D chain |
| SNX3 | 0.7827 | | O60493 | Sorting nexin-3 |
| SNTA1 | 0.7827 | | Q13424 | Alpha-1-syntrophin |
| RIF1 | 0.7827 | | Q5UIP0 | Telomere-associated protein RIF1 |
| Q6IAM1 | 0.7827 | | Q6IAM1 | Family with sequence similarity 107 member A transcript variant |
| Q1ZYQ1 | 0.7827 | | Q1ZYQ1 | TUBA2 {ECO:0000313\|EMBL:ABD72607.1} |
| PTEN | 0.7827 | | P60484 | Phosphatidylinositol 3,4,5-trisphosphate 3-phosphatase and dual-specificity protein phosphatase PTEN |
| PLAK | 0.7827 | | P14923 | Junction plakoglobin |
| NFKB1 | 0.7827 | | P19838 | Nuclear factor NF-kappa-B p105 subunit |
| MTOR | 0.7827 | | P42345 | Serine/threonine-protein kinase mTOR |
| KLH20 | 0.7827 | | Q9Y2M5 | Kelch-like protein 20 |
| F107A | 0.7827 | | O95990 | Protein FAM107A |
| DVL2 | 0.7827 | | O14641 | Segment polarity protein dishevelled homolog DVL-2 |
| COF1 | 0.7827 | | P23528 | Cofilin-1 |
| B1PKY1 | 0.7827 | | B1PKY1 | MHC Class I Antigen |
| 1A03 | 0.7827 | | P04439 | HLA class I histocompatibility antigen, A-3 alpha chain precursor |
| V9HWI3 | 0.7813 | | V9HWI3 | HEL-S-130P {ECO:0000313\|EMBL:ACJ13652.1} |
| TLE1 | 0.7813 | | Q04724 | Transducin-like enhancer protein 1 |
| TBB2B | 0.7813 | | Q9BVA1 | Tubulin beta-2B chain |
| S10A6 | 0.7813 | | P06703 | Protein S100-A6 |
| PPARA | 0.7813 | | Q07869 | Peroxisome proliferator-activated receptor alpha |
| NTRK1 | 0.7813 | | P04629 | High affinity nerve growth factor receptor precursor |
| MPP6 | 0.7813 | | Q9NZW5 | MAGUK p55 subfamily member 6 |
| JAK3 | 0.7813 | | P52333 | Tyrosine-protein kinase JAK3 |
| F1D8S4 | 0.7813 | | F1D8S4 | NR1C1 {ECO:0000313\|EMBL:ADZ17373.1} |
| F16P1 | 0.7813 | | P09467 | Fructose-1,6-bisphosphatase 1 |
| CATD | 0.7813 | | P07339 | Cathepsin D precursor |
| ACTN2 | 0.7813 | | P35609 | Alpha-actinin-2 |
| ZN417 | 0.7800 | | Q8TAU3 | Zinc finger protein 417 |
| VINC | 0.7800 | | P18206 | Vinculin |
| V9HWK2 | 0.7800 | | V9HWK2 | HEL114 {ECO:0000313\|EMBL:ACJ13727.1} |
| PTN12 | 0.7800 | | Q05209 | Tyrosine-protein phosphatase non-receptor type 12 |
| PPIB | 0.7800 | | P23284 | Peptidyl-prolyl cis-trans isomerase B precursor |
| MYL9 | 0.7800 | | P24844 | Myosin regulatory light polypeptide 9 |
| M3K8 | 0.7800 | | P41279 | Mitogen-activated protein kinase kinase kinase 8 |
| KPCZ | 0.7800 | | Q05513 | Protein kinase C zeta type |
| IKBA | 0.7800 | | P25963 | NF-kappa-B inhibitor alpha |
| IGF1R | 0.7800 | | P08069 | Insulin-like growth factor 1 receptor precursor |
| HNRPU | 0.7800 | | Q00839 | Heterogeneous nuclear ribonucleoprotein U |
| FSCN1 | 0.7800 | | Q16658 | Fascin |
| FAK1 | 0.7800 | | Q05397 | Focal adhesion kinase 1 |
| CASL | 0.7800 | | Q14511 | Enhancer of filamentation 1 |
| BMR1B | 0.7800 | | O00238 | Bone morphogenetic protein receptor type-1B precursor |
| 3BP2 | 0.7800 | | P78314 | SH3 domain-binding protein 2 |
| V9HWF5 | 0.7787 | | V9HWF5 | HEL-S-69p {ECO:0000313\|EMBL:ACJ13704.1} |
| TCP4 | 0.7787 | | P53999 | Activated RNA polymerase II transcriptional coactivator p15 |
| SNW1 | 0.7787 | | Q13573 | SNW domain-containing protein 1 |
| RHOJ | 0.7787 | | Q9H4E5 | Rho-related GTP-binding protein RhoJ precursor |
| Q6IBQ5 | 0.7787 | | Q6IBQ5 | FUS protein |
| Q6IBA2 | 0.7787 | | Q6IBA2 | PC4 {ECO:0000313\|EMBL:CAG33183.1} |
| Q59G17 | 0.7787 | | Q59G17 | N-methyl-D-aspartate receptor subunit 2D variant |
| PRGC1 | 0.7787 | | Q9UBK2 | Peroxisome proliferator-activated receptor gamma coactivator 1-alpha |
| PPIA | 0.7787 | | P62937 | Peptidyl-prolyl cis-trans isomerase A |
| NMDE4 | 0.7787 | | O15399 | Glutamate receptor ionotropic, NMDA 2D precursor |
| L7RTI5 | 0.7787 | | L7RTI5 | PRKCE {ECO:0000313\|EMBL:AGC09605.1} |
| KPCE | 0.7787 | | Q02156 | Protein kinase C epsilon type |
| KAT2B | 0.7787 | | Q92831 | Histone acetyltransferase KAT2B |
| ITB3 | 0.7787 | | P05106 | Integrin beta-3 precursor |
| ITB2 | 0.7787 | | P05107 | Integrin beta-2 precursor |
| HCK | 0.7787 | | P08631 | Tyrosine-protein kinase HCK |
| GNA13 | 0.7787 | | Q14344 | Guanine nucleotide-binding protein subunit alpha-13 |
| GATA4 | 0.7787 | | P43694 | Transcription factor GATA-4 |
| FXR2 | 0.7787 | | P51116 | Fragile X mental retardation syndrome-related protein 2 |
| FUS | 0.7787 | | P35637 | RNA-binding protein FUS |
| CDN1A | 0.7787 | | P38936 | Cyclin-dependent kinase inhibitor 1 |
| CDK3 | 0.7787 | | Q00526 | Cyclin-dependent kinase 3 |
| ZAP70 | 0.7773 | | P43403 | Tyrosine-protein kinase ZAP-70 |
| XRCC5 | 0.7773 | | P13010 | X-ray repair cross-complementing protein 5 |
| WASL | 0.7773 | | O00401 | Neural Wiskott-Aldrich syndrome protein |
| V9HWD0 | 0.7773 | | V9HWD0 | HEL-S-42 {ECO:0000313\|EMBL:ACJ13711.1} |
| U119A | 0.7773 | | Q13432 | Protein unc-119 homolog A |
| T2FA | 0.7773 | | P35269 | General transcription factor IIF subunit 1 |
| RHOQ | 0.7773 | | P17081 | Rho-related GTP-binding protein RhoQ precursor |
| RECQ5 | 0.7773 | | O94762 | ATP-dependent DNA helicase Q5 |
| RD23A | 0.7773 | | P54725 | UV excision repair protein RAD23 homolog A |
| RALA | 0.7773 | | P11233 | Ras-related protein Ral-A precursor |
| PLD2 | 0.7773 | | O14939 | Phospholipase D2 |
| ODO2 | 0.7773 | | P36957 | Dihydrolipoyllysine-residue succinyltransferase component of 2-oxoglutarate dehydrogenase complex, mitochondrial precursor |
| INSR | 0.7773 | | P06213 | Insulin receptor precursor |
| HXC8 | 0.7773 | | P31273 | Homeobox protein Hox-C8 |
| HIF1A | 0.7773 | | Q16665 | Hypoxia-inducible factor 1-alpha |
| D0VY79 | 0.7773 | | D0VY79 | HIF1A {ECO:0000313\|EMBL:BAI49183.1} |
| A8K4G2 | 0.7773 | | A8K4G2 | Tyrosine-protein kinase |
| TPA | 0.7760 | | P00750 | Tissue-type plasminogen activator precursor |
| SNAPN | 0.7760 | | O95295 | SNARE-associated protein Snapin |
| SH3G3 | 0.7760 | | Q99963 | Endophilin-A3 |
| RUVB2 | 0.7760 | | Q9Y230 | RuvB-like 2 |
| RBL2 | 0.7760 | | Q08999 | Retinoblastoma-like protein 2 |
| NR4A1 | 0.7760 | | P22736 | Nuclear receptor subfamily 4 group A member 1 |
| MCM3 | 0.7760 | | P25205 | DNA replication licensing factor MCM3 |
| L7RT22 | 0.7760 | | L7RT22 | ITGB5 {ECO:0000313\|EMBL:AGC09593.1} |
| K1C15 | 0.7760 | | P19012 | Keratin, type I cytoskeletal 15 |
| ITPR1 | 0.7760 | | Q14643 | Inositol 1,4,5-trisphosphate receptor type 1 |
| ITB5 | 0.7760 | | P18084 | Integrin beta-5 precursor |
| IKBB | 0.7760 | | Q15653 | NF-kappa-B inhibitor beta |
| IF4A1 | 0.7760 | | P60842 | Eukaryotic initiation factor 4A-I |
| FRIL | 0.7760 | | P02792 | Ferritin light chain |
| EXOSX | 0.7760 | | Q01780 | Exosome component 10 |
| DNJA1 | 0.7760 | | P31689 | DnaJ homolog subfamily A member 1 precursor |
| DHX9 | 0.7760 | | Q08211 | ATP-dependent RNA helicase A |
| CD82 | 0.7760 | | P27701 | CD82 antigen |
| ARRB2 | 0.7760 | | P32121 | Beta-arrestin-2 |
| AHR | 0.7760 | | P35869 | Aryl hydrocarbon receptor precursor |
| X5DNI1 | 0.7747 | | X5DNI1 | Collapsin response mediator protein 1 isoform A |
| TBA1B | 0.7747 | | P68363 | Tubulin alpha-1B chain |
| STF1 | 0.7747 | | Q13285 | Steroidogenic factor 1 |
| STA5A | 0.7747 | | P42229 | Signal transducer and activator of transcription 5A |
| SRF | 0.7747 | | P11831 | Serum response factor |
| SMN | 0.7747 | | Q16637 | Survival motor neuron protein |
| RL5 | 0.7747 | | P46777 | 60S ribosomal protein L5 |
| Q13280 | 0.7747 | | Q13280 | Calcium/calmodulin-dependent protein kinase II |
| PP1A | 0.7747 | | P62136 | Serine/threonine-protein phosphatase PP1-alpha catalytic subunit |
| MEN1 | 0.7747 | | O00255 | Menin |
| ITAV | 0.7747 | | P06756 | Integrin alpha-V precursor |
| IRS2 | 0.7747 | | Q9Y4H2 | Insulin receptor substrate 2 |
| GNA12 | 0.7747 | | Q03113 | Guanine nucleotide-binding protein subunit alpha-12 |
| F1D8R8 | 0.7747 | | F1D8R8 | NR5A1 {ECO:0000313\|EMBL:ADZ17367.1} |
| COX17 | 0.7747 | | Q14061 | Cytochrome c oxidase copper chaperone |
| CO3A1 | 0.7747 | | P02461 | Collagen alpha-1(III) chain precursor |
| CATG | 0.7747 | | P08311 | Cathepsin G precursor |
| C1QA | 0.7747 | | P02745 | Complement C1q subcomponent subunit A precursor |
| A8K840 | 0.7747 | | A8K840 | . |
| A2RUM7 | 0.7747 | | A2RUM7 | RPL5 {ECO:0000313\|EMBL:AAI32971.1} |
| WDR5 | 0.7733 | | P61964 | WD repeat-containing protein 5 |
| SRSF1 | 0.7733 | | Q07955 | Serine/arginine-rich splicing factor 1 |
| RB11A | 0.7733 | | P62491 | Ras-related protein Rab-11A precursor |
| Q7Z726 | 0.7733 | | Q7Z726 | KPNA2 {ECO:0000313\|EMBL:AAH53343.1} |
| Q6IBN1 | 0.7733 | | Q6IBN1 | HNRPK protein |
| PNMA1 | 0.7733 | | Q8ND90 | Paraneoplastic antigen Ma1 |
| LRP1 | 0.7733 | | Q07954 | Prolow-density lipoprotein receptor-related protein 1 precursor |
| IRAK1 | 0.7733 | | P51617 | Interleukin-1 receptor-associated kinase 1 |
| FXR1 | 0.7733 | | P51114 | Fragile X mental retardation syndrome-related protein 1 |
| CT024 | 0.7733 | | Q9BUV8 | Uncharacterized protein C20orf24 |
| VDR | 0.7720 | | P11473 | Vitamin D3 receptor |
| SRBP2 | 0.7720 | | Q12772 | Sterol regulatory element-binding protein 2 |
| Q5U0J5 | 0.7720 | | Q5U0J5 | CREB1 {ECO:0000313\|EMBL:EAW70405.1} |
| PHYIP | 0.7720 | | Q92561 | Phytanoyl-CoA hydroxylase-interacting protein |
| FGF1 | 0.7720 | | P05230 | Fibroblast growth factor 1 precursor |
| F1D8P8 | 0.7720 | | F1D8P8 | NR1i1 {ECO:0000313\|EMBL:ADZ17347.1} |
| CREB1 | 0.7720 | | P16220 | Cyclic AMP-responsive element-binding protein 1 |
| BAG6 | 0.7720 | | P46379 | Large proline-rich protein BAG6 |
| THRB | 0.7707 | | P00734 | Prothrombin precursor |
| ST1E1 | 0.7707 | | P49888 | Estrogen sulfotransferase |
| SNIP1 | 0.7707 | | Q8TAD8 | Smad nuclear-interacting protein 1 |
| RFA1 | 0.7707 | | P27694 | Replication protein A 70 kDa DNA-binding subunit |
| Q6NVW1 | 0.7707 | | Q6NVW1 | EPHB2 protein |
| Q6IBN6 | 0.7707 | | Q6IBN6 | CBX1 {ECO:0000313\|EMBL:CAG33047.1} |
| Q53X91 | 0.7707 | | Q53X91 | Sulfotransferase |
| HMGN2 | 0.7707 | | P05204 | Non-histone chromosomal protein HMG-17 |
| CD5 | 0.7707 | | P06127 | T-cell surface glycoprotein CD5 precursor |
| CBX1 | 0.7707 | | P83916 | Chromobox protein homolog 1 |
| B1AK66 | 0.7707 | | B1AK66 | SNIP1 {ECO:0000313\|EMBL:EAX07338.1} |
| SVIL | 0.7693 | | O95425 | Supervillin |
| Q569J5 | 0.7693 | | Q569J5 | SVIL protein |
| Q569H8 | 0.7693 | | Q569H8 | ESRRA {ECO:0000313\|EMBL:AAH92470.1} |
| PDPK1 | 0.7693 | | O15530 | 3-phosphoinositide-dependent protein kinase 1 |
| MIC60 | 0.7693 | | Q16891 | MICOS complex subunit MIC60 precursor |
| M4K1 | 0.7693 | | Q92918 | Mitogen-activated protein kinase kinase kinase kinase 1 |
| ERR1 | 0.7693 | | P11474 | Steroid hormone receptor ERR1 |
| ERBB3 | 0.7693 | | P21860 | Receptor tyrosine-protein kinase erbB-3 precursor |
| CDC6 | 0.7693 | | Q99741 | Cell division control protein 6 homolog |
| APBB1 | 0.7693 | | O00213 | Amyloid beta A4 precursor protein-binding family B member 1 |
| XPA | 0.7680 | | P23025 | DNA repair protein complementing XP-A cells |
| WNT3A | 0.7680 | | P56704 | Protein Wnt-3a precursor |
| PTN1 | 0.7680 | | P18031 | Tyrosine-protein phosphatase non-receptor type 1 |
| PSD11 | 0.7680 | | O00231 | 26S proteasome non-ATPase regulatory subunit 11 |
| PRKDC | 0.7680 | | P78527 | DNA-dependent protein kinase catalytic subunit |
| PR40A | 0.7680 | | O75400 | Pre-mRNA-processing factor 40 homolog A |
| MED1 | 0.7680 | | Q15648 | Mediator of RNA polymerase II transcription subunit 1 |
| GRB14 | 0.7680 | | Q14449 | Growth factor receptor-bound protein 14 |
| FBW1A | 0.7680 | | Q9Y297 | F-box/WD repeat-containing protein 1A |
| CCNT1 | 0.7680 | | O60563 | Cyclin-T1 |
| C1QBP | 0.7680 | | Q07021 | Complement component 1 Q subcomponent-binding protein, mitochondrial precursor |
| A8K3M3 | 0.7680 | | A8K3M3 | Tyrosine-protein phosphatase non-receptor type |
| STX8 | 0.7667 | | Q9UNK0 | Syntaxin-8 |
| STABP | 0.7667 | | O95630 | STAM-binding protein |
| RGS19 | 0.7667 | | P49795 | Regulator of G-protein signaling 19 |
| PIAS4 | 0.7667 | | Q8N2W9 | E3 SUMO-protein ligase PIAS4 |
| FRIH | 0.7667 | | P02794 | Ferritin heavy chain |
| ATR | 0.7667 | | Q13535 | Serine/threonine-protein kinase ATR |
| ASF1B | 0.7667 | | Q9NVP2 | Histone chaperone ASF1B |
| A8K5M4 | 0.7667 | | A8K5M4 | . |
| UBE2U | 0.7653 | | Q5VVX9 | Ubiquitin-conjugating enzyme E2 U |
| TRIP6 | 0.7653 | | Q15654 | Thyroid receptor-interacting protein 6 |
| TRIB3 | 0.7653 | | Q96RU7 | Tribbles homolog 3 |
| TNR1B | 0.7653 | | P20333 | Tumor necrosis factor receptor superfamily member 1B precursor |
| SOCS1 | 0.7653 | | O15524 | Suppressor of cytokine signaling 1 |
| RNPS1 | 0.7653 | | Q15287 | RNA-binding protein with serine-rich domain 1 |
| Q53HG7 | 0.7653 | | Q53HG7 | Cortactin isoform a variant |
| Q53G92 | 0.7653 | | Q53G92 | . |
| Q4JHT5 | 0.7653 | | Q4JHT5 | SOCS1 {ECO:0000313\|EMBL:AAY87931.1} |
| PEBP1 | 0.7653 | | P30086 | Phosphatidylethanolamine-binding protein 1 |
| MYOD1 | 0.7653 | | P15172 | Myoblast determination protein 1 |
| DVL3 | 0.7653 | | Q92997 | Segment polarity protein dishevelled homolog DVL-3 |
| DCTN1 | 0.7653 | | Q14203 | Dynactin subunit 1 |
| D9IAI1 | 0.7653 | | D9IAI1 | PEBP1 {ECO:0000313\|EMBL:EAW98123.1} |
| D3DU92 | 0.7653 | | D3DU92 | RNPS1 {ECO:0000313\|EMBL:EAW85517.1} |
| CSN6 | 0.7653 | | Q7L5N1 | COP9 signalosome complex subunit 6 |
| CO4A1 | 0.7653 | | P02462 | Collagen alpha-1(IV) chain precursor |
| ZBT17 | 0.7640 | | Q13105 | Zinc finger and BTB domain-containing protein 17 |
| Z512B | 0.7640 | | Q96KM6 | Zinc finger protein 512B |
| VINEX | 0.7640 | | O60504 | Vinexin |
| VGFR1 | 0.7640 | | P17948 | Vascular endothelial growth factor receptor 1 precursor |
| VAMP2 | 0.7640 | | P63027 | Vesicle-associated membrane protein 2 |
| RPB1 | 0.7640 | | P24928 | DNA-directed RNA polymerase II subunit RPB1 |
| NOS1 | 0.7640 | | P29475 | Nitric oxide synthase, brain |
| L7RSL3 | 0.7640 | | L7RSL3 | FLT1 {ECO:0000313\|EMBL:AGC09589.1} |
| KLF6 | 0.7640 | | Q99612 | Krueppel-like factor 6 |
| GRB7 | 0.7640 | | Q14451 | Growth factor receptor-bound protein 7 |
| CTNA1 | 0.7640 | | P35221 | Catenin alpha-1 |
| B3VK56 | 0.7640 | | B3VK56 | NOS1 {ECO:0000313\|EMBL:EAW98110.1} |
| V9HWF0 | 0.7627 | | V9HWF0 | HEL-S-28 {ECO:0000313\|EMBL:ACJ13679.1} |
| UFO | 0.7627 | | P30530 | Tyrosine-protein kinase receptor UFO precursor |
| UB2E1 | 0.7627 | | P51965 | Ubiquitin-conjugating enzyme E2 E1 |
| SHIP1 | 0.7627 | | Q92835 | Phosphatidylinositol 3,4,5-trisphosphate 5-phosphatase 1 |
| ROCK1 | 0.7627 | | Q13464 | Rho-associated protein kinase 1 |
| RASM | 0.7627 | | O14807 | Ras-related protein M-Ras precursor |
| Q8WVM9 | 0.7627 | | Q8WVM9 | Putative uncharacterized protein |
| Q6FGP0 | 0.7627 | | Q6FGP0 | MRAS {ECO:0000313\|EMBL:CAG46864.1} |
| PROF2 | 0.7627 | | P35080 | Profilin-2 |
| PAK4 | 0.7627 | | O96013 | Serine/threonine-protein kinase PAK 4 |
| M3K1 | 0.7627 | | Q13233 | Mitogen-activated protein kinase kinase kinase 1 |
| ILK | 0.7627 | | Q13418 | Integrin-linked protein kinase |
| DEDD | 0.7627 | | O75618 | Death effector domain-containing protein |
| CDC20 | 0.7627 | | Q12834 | Cell division cycle protein 20 homolog |
| CD81 | 0.7627 | | P60033 | CD81 antigen |
| ARHG7 | 0.7627 | | Q14155 | Rho guanine nucleotide exchange factor 7 |
| USBP1 | 0.7613 | | Q8N6Y0 | Usher syndrome type-1C protein-binding protein 1 |
| SSR2 | 0.7613 | | P30874 | Somatostatin receptor type 2 |
| RUXG | 0.7613 | | P62308 | Small nuclear ribonucleoprotein G |
| ITB7 | 0.7613 | | P26010 | Integrin beta-7 precursor |
| ITB1 | 0.7613 | | P05556 | Integrin beta-1 precursor |
| EXOS8 | 0.7613 | | Q96B26 | Exosome complex component RRP43 |
| COIL | 0.7613 | | P38432 | Coilin |
| B3KNS9 | 0.7613 | | B3KNS9 | . |
| B2RUU2 | 0.7613 | | B2RUU2 | ABCA1 {ECO:0000313\|EMBL:AAI46857.1} |
| TYK2 | 0.7600 | | P29597 | Non-receptor tyrosine-protein kinase TYK2 |
| TRAF1 | 0.7600 | | Q13077 | TNF receptor-associated factor 1 |
| TIAM1 | 0.7600 | | Q13009 | T-lymphoma invasion and metastasis-inducing protein 1 |
| TCEA1 | 0.7600 | | P23193 | Transcription elongation factor A protein 1 |
| RUNX2 | 0.7600 | | Q13950 | Runt-related transcription factor 2 |
| RAP1A | 0.7600 | | P62834 | Ras-related protein Rap-1A precursor |
| NINL | 0.7600 | | Q9Y2I6 | Ninein-like protein |
| MYH9 | 0.7600 | | P35579 | Myosin-9 |
| FURIN | 0.7600 | | P09958 | Furin precursor |
| FLNC | 0.7600 | | Q14315 | Filamin-C |
| FADD | 0.7600 | | Q13158 | FAS-associated death domain protein |
| DDX20 | 0.7600 | | Q9UHI6 | Probable ATP-dependent RNA helicase DDX20 |
| CBPE | 0.7600 | | P16870 | Carboxypeptidase E precursor |
| ZN107 | 0.7587 | | Q9UII5 | Zinc finger protein 107 |
| V9HWG0 | 0.7587 | | V9HWG0 | HEL25 {ECO:0000313\|EMBL:ACJ13653.1} |
| TSP1 | 0.7587 | | P07996 | Thrombospondin-1 precursor |
| SQSTM | 0.7587 | | Q13501 | Sequestosome-1 |
| SH3K1 | 0.7587 | | Q96B97 | SH3 domain-containing kinase-binding protein 1 |
| RASA1 | 0.7587 | | P20936 | Ras GTPase-activating protein 1 |
| Q9UQS6 | 0.7587 | | Q9UQS6 | Fibronectin |
| Q86X89 | 0.7587 | | Q86X89 | MAP1B protein |
| Q659G9 | 0.7587 | | Q659G9 | Putative uncharacterized protein DKFZp586H0919 |
| Q59GK3 | 0.7587 | | Q59GK3 | RAS p21 protein activator 1 isoform 1 variant |
| Q0EAF5 | 0.7587 | | Q0EAF5 | CD22 {ECO:0000313\|EMBL:BAF32758.1} |
| PUF60 | 0.7587 | | Q9UHX1 | Poly(U)-binding-splicing factor PUF60 |
| NOS3 | 0.7587 | | P29474 | Nitric oxide synthase, endothelial |
| MED14 | 0.7587 | | O60244 | Mediator of RNA polymerase II transcription subunit 14 |
| MAP1B | 0.7587 | | P46821 | Microtubule-associated protein 1B |
| LRP2 | 0.7587 | | P98164 | Low-density lipoprotein receptor-related protein 2 precursor |
| LEF1 | 0.7587 | | Q9UJU2 | Lymphoid enhancer-binding factor 1 |
| F1D8R0 | 0.7587 | | F1D8R0 | NR2F2 {ECO:0000313\|EMBL:ADZ17359.1} |
| EXOS4 | 0.7587 | | Q9NPD3 | Exosome complex component RRP41 |
| EPHA2 | 0.7587 | | P29317 | Ephrin type-A receptor 2 precursor |
| COT2 | 0.7587 | | P24468 | COUP transcription factor 2 |
| CD22 | 0.7587 | | P20273 | B-cell receptor CD22 precursor |
| CBX5 | 0.7587 | | P45973 | Chromobox protein homolog 5 |
| CASP9 | 0.7587 | | P55211 | Caspase-9 precursor |
| B2RUU3 | 0.7587 | | B2RUU3 | DOCK1 {ECO:0000313\|EMBL:AAI46858.1} |
| T22D1 | 0.7573 | | Q15714 | TSC22 domain family protein 1 |
| Q6IB83 | 0.7573 | | Q6IB83 | BHLHB2 protein |
| Q60FE5 | 0.7573 | | Q60FE5 | FLNA {ECO:0000313\|EMBL:BAD52436.1, |
| Q59F94 | 0.7573 | | Q59F94 | Ataxin-1 ubiquitin-like interacting protein variant |
| Q53GZ6 | 0.7573 | | Q53GZ6 | . |
| PP2BA | 0.7573 | | Q08209 | Serine/threonine-protein phosphatase 2B catalytic subunit alpha isoform |
| PHLA3 | 0.7573 | | Q9Y5J5 | Pleckstrin homology-like domain family A member 3 |
| PGFRA | 0.7573 | | P16234 | Platelet-derived growth factor receptor alpha precursor |
| IL6RB | 0.7573 | | P40189 | Interleukin-6 receptor subunit beta precursor |
| IF6 | 0.7573 | | P56537 | Eukaryotic translation initiation factor 6 |
| HOME3 | 0.7573 | | Q9NSC5 | Homer protein homolog 3 |
| CCG2 | 0.7573 | | Q9Y698 | Voltage-dependent calcium channel gamma-2 subunit |
| BHE40 | 0.7573 | | O14503 | Class E basic helix-loop-helix protein 40 |
| TIF1A | 0.7560 | | O15164 | Transcription intermediary factor 1-alpha |
| TEC | 0.7560 | | P42680 | Tyrosine-protein kinase Tec |
| SHIP2 | 0.7560 | | O15357 | Phosphatidylinositol 3,4,5-trisphosphate 5-phosphatase 2 |
| SCAM1 | 0.7560 | | O15126 | Secretory carrier-associated membrane protein 1 |
| RBM23 | 0.7560 | | Q86U06 | Probable RNA-binding protein 23 |
| RAD21 | 0.7560 | | O60216 | Double-strand-break repair protein rad21 homolog |
| Q05CZ1 | 0.7560 | | Q05CZ1 | SYNJ1 protein |
| PKN1 | 0.7560 | | Q16512 | Serine/threonine-protein kinase N1 |
| MYH10 | 0.7560 | | P35580 | Myosin-10 |
| KPCG | 0.7560 | | P05129 | Protein kinase C gamma type |
| K1549 | 0.7560 | | Q9HCM3 | UPF0606 protein KIAA1549 |
| J3KQV8 | 0.7560 | | J3KQV8 | SYNJ1 {ECO:0000313\|Ensembl:ENSP00000409667} |
| EHMT2 | 0.7560 | | Q96KQ7 | Histone-lysine N-methyltransferase EHMT2 |
| DAG1 | 0.7560 | | Q14118 | Dystroglycan precursor |
| ZMY11 | 0.7547 | | Q15326 | Zinc finger MYND domain-containing protein 11 |
| UTRO | 0.7547 | | P46939 | Utrophin |
| TRAF5 | 0.7547 | | O00463 | TNF receptor-associated factor 5 |
| TNR5 | 0.7547 | | P25942 | Tumor necrosis factor receptor superfamily member 5 precursor |
| TERF2 | 0.7547 | | Q15554 | Telomeric repeat-binding factor 2 |
| SDC3 | 0.7547 | | O75056 | Syndecan-3 |
| Q7KZS0 | 0.7547 | | Q7KZS0 | SUMO-conjugating enzyme |
| Q6LBS5 | 0.7547 | | Q6LBS5 | Dystrophin-related protein (490 AA) |
| PTPRS | 0.7547 | | Q13332 | Receptor-type tyrosine-protein phosphatase S precursor |
| PRS8 | 0.7547 | | P62195 | 26S protease regulatory subunit 8 |
| NCOA3 | 0.7547 | | Q9Y6Q9 | Nuclear receptor coactivator 3 |
| NCALD | 0.7547 | | P61601 | Neurocalcin-delta |
| MTG8 | 0.7547 | | Q06455 | Protein CBFA2T1 |
| KMT2A | 0.7547 | | Q03164 | Histone-lysine N-methyltransferase 2A |
| HDAC2 | 0.7547 | | Q92769 | Histone deacetylase 2 |
| B3KSH4 | 0.7547 | | B3KSH4 | Huntingtin interacting protein 2, isoform CRA_a |
| B2RB70 | 0.7547 | | B2RB70 | NCALD {ECO:0000313\|EMBL:EAW91835.1} |
| ARNT | 0.7547 | | P27540 | Aryl hydrocarbon receptor nuclear translocator |
| TBG1 | 0.7533 | | P23258 | Tubulin gamma-1 chain |
| SUH | 0.7533 | | Q06330 | Recombining binding protein suppressor of hairless |
| STA13 | 0.7533 | | Q9Y3M8 | StAR-related lipid transfer protein 13 |
| RON | 0.7533 | | Q04912 | Macrophage-stimulating protein receptor precursor |
| RGAP1 | 0.7533 | | Q9H0H5 | Rac GTPase-activating protein 1 |
| Q32Q75 | 0.7533 | | Q32Q75 | Eukaryotic translation initiation factor 4E |
| PLD1 | 0.7533 | | Q13393 | Phospholipase D1 |
| MMP7 | 0.7533 | | P09237 | Matrilysin precursor |
| MAST2 | 0.7533 | | Q6P0Q8 | Microtubule-associated serine/threonine-protein kinase 2 |
| M3K14 | 0.7533 | | Q99558 | Mitogen-activated protein kinase kinase kinase 14 |
| LAMA4 | 0.7533 | | Q16363 | Laminin subunit alpha-4 precursor |
| KS6B1 | 0.7533 | | P23443 | Ribosomal protein S6 kinase beta-1 |
| FGFR3 | 0.7533 | | P22607 | Fibroblast growth factor receptor 3 precursor |
| EXOS7 | 0.7533 | | Q15024 | Exosome complex component RRP42 |
| DOCK1 | 0.7533 | | Q14185 | Dedicator of cytokinesis protein 1 |
| CO9A2 | 0.7533 | | Q14055 | Collagen alpha-2(IX) chain precursor |
| AGO2 | 0.7533 | | Q9UKV8 | Protein argonaute-2 |
| V9HWE0 | 0.7520 | | V9HWE0 | HEL-S-7 {ECO:0000313\|EMBL:ACJ13644.1} |
| SPTB2 | 0.7520 | | Q01082 | Spectrin beta chain, non-erythrocytic 1 |
| RND1 | 0.7520 | | Q92730 | Rho-related GTP-binding protein Rho6 precursor |
| RENI | 0.7520 | | P00797 | Renin precursor |
| NMDE2 | 0.7520 | | Q13224 | Glutamate receptor ionotropic, NMDA 2B precursor |
| LC7L2 | 0.7520 | | Q9Y383 | Putative RNA-binding protein Luc7-like 2 |
| H2A1J | 0.7520 | | Q99878 | Histone H2A type 1-J |
| CHM4B | 0.7520 | | Q9H444 | Charged multivesicular body protein 4b |
| BCL3 | 0.7520 | | P20749 | B-cell lymphoma 3 protein |
| B2ZZ89 | 0.7520 | | B2ZZ89 | SPTBN1 {ECO:0000313\|EMBL:BAG48315.1} |
| B2R6D7 | 0.7520 | | B2R6D7 | . |
| ATG12 | 0.7520 | | O94817 | Ubiquitin-like protein ATG12 |
| ANXA5 | 0.7520 | | P08758 | Annexin A5 |
| WRN | 0.7507 | | Q14191 | Werner syndrome ATP-dependent helicase |
| UB2L3 | 0.7507 | | P68036 | Ubiquitin-conjugating enzyme E2 L3 |
| TRADD | 0.7507 | | Q15628 | Tumor necrosis factor receptor type 1-associated DEATH domain protein |
| RFXK | 0.7507 | | O14593 | DNA-binding protein RFXANK |
| Q8IY97 | 0.7507 | | Q8IY97 | Adaptor-related protein complex 1, gamma 1 subunit |
| Q6FI00 | 0.7507 | | Q6FI00 | CCND1 protein |
| Q5U003 | 0.7507 | | Q5U003 | CCR1 {ECO:0000313\|EMBL:EAW64756.1} |
| Q59F09 | 0.7507 | | Q59F09 | Werner syndrome protein variant |
| Q4LE70 | 0.7507 | | Q4LE70 | APC variant protein |
| PSB5 | 0.7507 | | P28074 | Proteasome subunit beta type-5 precursor |
| OPSD | 0.7507 | | P08100 | Rhodopsin |
| NSF | 0.7507 | | P46459 | Vesicle-fusing ATPase |
| LDB1 | 0.7507 | | Q86U70 | LIM domain-binding protein 1 |
| HDAC4 | 0.7507 | | P56524 | Histone deacetylase 4 |
| GRB10 | 0.7507 | | Q13322 | Growth factor receptor-bound protein 10 |
| CSF1R | 0.7507 | | P07333 | Macrophage colony-stimulating factor 1 receptor precursor |
| CO4A4 | 0.7507 | | P53420 | Collagen alpha-4(IV) chain precursor |
| CCR1 | 0.7507 | | P32246 | C-C chemokine receptor type 1 |
| CCND1 | 0.7507 | | P24385 | G1/S-specific cyclin-D1 |
| B7XCW9 | 0.7507 | | B7XCW9 | ABCA1 {ECO:0000313\|EMBL:BAH10664.1} |
| APC | 0.7507 | | P25054 | Adenomatous polyposis coli protein |
| ABI1 | 0.7507 | | Q8IZP0 | Abl interactor 1 |

**Table S2:** List of common genes predicted as TRPM7 interactors using proteinprompt and correlated with TRPM7 in TCGA datasets.

| Dataset | Number | List of Potential interactors (ProteinPrompt + correlated in TCGA dataset) |
| --- | --- | --- |
| Adrenocortical Cancer (ACC) | 7 | EP300 SMAD3 COX17 MED14 SIN3A KMT2A CRKL |
| Acute Myeloid Leukemia (LAML) | 101 | EP300 TRAF5 CDK8 CDK1 BCL2 ATR MED14 UBE2W SVIL SET NCOA6 TRAF6 BRCA2 OCLN GNAI1 EGFR CREB1 NCOA1 KNG1 RAP2A NCK1 RASA1 MBP ATG12 TERF1 DDX20 HMGB1 SKP2 PLS1 RIF1 ITPR1 ATM RBL2 SMAD4 FXR1 CASP3 CUL1 DHX9 GRB10 LRIF1 BMPR2 SP1 XPO1 MED1 MRE11 TOP2A TEC HIF1A WRN KMT2A JAK1 CBX5 CRKL PAK2 SMAD5 ZNF24 SMAD9 NCOA3 AGO2 KIT BARD1 EPS15 PRKDC ESR1 GNA13 STAT1 DOCK1 SMAD2 SMAD1 CCNT1 LRP2 TCEA1 XRCC5 APC ESR2 LNX1 KAT2B WASL SOS1 PTEN TAF7 COIL NRIP1 GFI1B RBBP4 MYH10 SIN3A PARP1 ROCK1 DAB2 PPARA CBL ABI1 RALA GNAQ XPA SRSF1 HDAC2 MAP1B RAD21 LAMA4 |
| Bladder urothelial carcinoma (BLCA) | 123 | EP300 TRAF5 CDK8 THRB IRS1 ATR MED14 UBE2W AHR SVIL RAB5A NCOA6 TRAF6 BRCA2 OCLN MDM2 EGFR ERBB2 CREB1 RAB4A NCOR1 NCOA1 RHOQ CDK2 RAP2A RASA1 MBP SKIL TERF1 PEBP1 DDX20 ERBB4 RXRA NOS1 SNIP1 SMAD3 PLCG1 SKP2 PLS1 RIF1 ATN1 ITPR1 ATM CD9 RBL2 SMAD4 CASP3 DHX9 GRB10 LRIF1 BMPR2 SP1 ERBB3 XPO1 MED1 MRE11 TOP2A TEC WRN KMT2A LMO3 INSR JAK1 HIPK2 CRKL PAK2 GATA4 SMAD5 ZNF24 SMAD9 FGFR3 TIAM1 NCOA3 CAV3 KIT BARD1 EPS15 PRKDC IRS2 DAG1 GNA13 DOCK1 SMAD2 CCNT1 TCEA1 SRC CASP9 LDB1 RNF11 APC ESR2 LNX1 CDK3 KAT2B WASL SOS1 PTEN DVL2 ARNT ABCA1 COIL NRIP1 PPARG GFI1B MTOR RAF1 RBBP4 PDPK1 SIN3A ROCK1 PTPRS DAB2 PPARA DVL3 NSF CBL ABI1 GNAQ HOOK2 SRSF1 CRK RAD21 VHL |
| Breast invasive carcinoma (BRCA) | 156 | EP300 CDK8 UBE2W BTK JAK2 RAB5A TRAF6 LRP1 MDM2 EGFR CREB1 CD28 ABL1 NCOR1 RUNX2 RAP2A RASA1 MBP TERF1 DDX20 ERBB4 NOS1 SMAD3 SKP1 KLF6 LEF1 STAT3 RBL2 FXR1 CASP3 LRIF1 CCR1 SP1 MRE11 WRN CRKL PAK2 SMAD5 FGFR1 ZNF24 SMAD9 NCOA3 EPS15 ESR1 DAG1 GNA13 STAT1 HDAC4 CCNT1 ACVR1 TCEA1 LCP2 CD22 XRCC5 NFKB1 RNF11 LNX1 KAT2B COIL PPARG SMAD7 PDPK1 DAB2 NSF CBL ABI1 GNAQ ETS1 VAMP3 CRK CCR5 RAD21 LAMA4 VHL CD44 TRAF5 BCL2 THRB IRS1 ATR FGF1 MED14 AHR SVIL TERF2 ITK NCOA6 BRCA2 OCLN NCOA1 RHOQ RHOJ NCK1 ATG12 SKIL RAP1A SNIP1 RBM23 SKP2 PLS1 RIF1 ITPR1 CDC42 ATM SMAD4 DHX9 BMPR2 ERBB3 XPO1 MED1 TOP2A IGF1R CAV1 TEC HIF1A KMT2A INSR JAK1 HIPK2 CBX5 CSF1R ARF6 PLD1 TIAM1 BARD1 CCND1 PRKDC IRS2 DOCK1 SMAD2 SPOP SMAD1 LRP2 CFLAR APC GNA12 VAMP2 WASL SOS1 PTEN TAF7 ARNT ABCA1 NRIP1 RHOA MTOR RAF1 RBBP4 MYH10 SIN3A ROCK1 PPARA EIF1B XPA SRSF1 MAP1B |
| Cervical squamous cell carcinoma and endocervical adenocarcinoma (CESC) | 110 | MMP2 EP300 NINL NCOR2 CDK8 CDK1 BCL2 ATR MED14 UBE2W SVIL JAK2 NCOA6 TRAF6 BRCA2 OCLN MDM2 EGFR CREB1 ABL1 NCOR1 NCOA1 RHOQ CDK2 RHOJ RAP2A RASA1 MBP SKIL TERF1 DDX20 ERBB4 NOS1 SNIP1 SMAD3 PLCG1 SKP2 LEF1 RIF1 ATN1 ITPR1 ATM RBL2 SMAD4 DHX9 BMPR2 SP1 XPO1 MED1 MRE11 TOP2A IGF1R WRN KMT2A JAK1 HIPK2 CBX5 CRKL SMAD5 FGFR1 ZNF24 SMAD9 TIAM1 NCOA3 PCBP1 AGO2 KIT BARD1 EPS15 CBX1 PRKDC DAG1 GNA13 DOCK1 SMAD2 HDAC4 SMAD1 CCNT1 ACVR1 TCEA1 PIAS4 NFKB1 LDB1 APC ESR2 KAT2B WASL SOS1 PTEN MAST2 ARNT COIL NRIP1 MTOR RBBP4 PDPK1 SIN3A ROCK1 PPARA NSF CBL ABI1 GNAQ SRSF1 ETS1 SRSF2 MAP1B CRK RAD21 VHL |
| Cholangiocarcinoma (CHOL) | 21 | WASL SOS1 EP300 MED14 RIF1 PDPK1 PRKDC ATM SIN3A RBL2 NCOA6 DOCK1 TRAF6 PPARA NSF EGFR MED1 CCNT1 RAB4A NCOR1 CRK |
| Colorectal adenocarcinoma (COADREAD) | 161 | CD44 SUMO1 EP300 TRAF5 CDK1 BCL2 ATR FGF1 MED14 UBE2W AHR UBE2N BTK SVIL SET JAK2 TERF2 RAB5A ITK SRSF3 SNW1 TRAF6 BRCA2 OCLN MDM2 GNAI1 EGFR CREB1 CD28 RAB4A NCOR1 NCOA1 RHOQ CDK2 RUNX2 MED31 RAP2A NCK1 RASA1 MBP ATG12 SKIL TERF1 DDX20 HMGB1 ERBB4 PTN RAP1A SNIP1 SMAD3 SKP1 SKP2 XRCC6 KLF6 RRAS2 LEF1 PLS1 RIF1 STAT3 ITPR1 CDC42 ATM CENPA RBL2 SMAD4 FXR1 CASP3 CUL1 DHX9 LRIF1 CCR1 LYN BMPR2 SUMO2 SP1 XPO1 MED1 MRE11 TOP2A IGF1R CAV1 TEC HIF1A WRN KMT2A LMO3 JAK1 HIPK2 CBX5 CRKL PAK2 SMAD5 ARF6 ZNF24 SMAD9 CDC6 PLD1 TIAM1 NCOA3 KIT BARD1 EPS15 CBX1 PRKDC ESR1 GNA13 STAT1 DOCK1 SMAD2 HDAC4 SPOP SMAD1 CCNT1 ACVR1 TCEA1 LCP2 CFLAR XRCC5 RAB1A NFKB1 RNF11 APC ESR2 KAT2B WASL SOS1 PTEN TAF7 ARNT ABCA1 COIL NRIP1 PPARG RHOA RAF1 RBBP4 MYH10 SIN3A GRB14 ANXA5 PARP1 ROCK1 DAB2 PPARA NSF EIF1B CBL ABI1 RALA GNAQ SRSF1 HDAC2 ETS1 VAMP3 SNX3 MAP1B CRK CCR5 RAD21 LAMA4 VHL |
| Esophagus carcinoma (ESCA) | 66 | EP300 TRAF5 NCOR2 CDK1 BCL2 ZNF24 ATR MED14 UBE2W AHR AGO2 BARD1 EPS15 PRKDC DAG1 GNA13 NCOA6 DOCK1 SMAD2 BRCA2 HDAC4 MDM2 CREB1 CCNT1 NCOR1 NCOA1 MCM2 CDK2 XRCC5 SKIL APC ESR2 SOS1 MAST2 ARNT SKP2 MTOR RIF1 RBBP4 PDPK1 ATN1 ATM SIN3A RBL2 SMAD4 CASP3 PARP1 DHX9 ROCK1 PPARA NSF BMPR2 CBL SP1 XPO1 MED1 SRSF1 MRE11 TOP2A WRN KMT2A JAK1 RAD21 CBX5 VHL SMAD5 |
| Glioblastoma (GBM) | 70 | EP300 FGFR1 TRAF5 NCOR2 NFKB2 BCL2 ATR MED14 WNT3A NCOA3 SVIL AGO2 JAK2 BARD1 EPS15 PRKDC GNA13 TRIP6 DOCK1 PLD2 BRCA2 LRP1 OCLN ERBB2 CREB1 CCNT1 NCOR1 NCOA1 RUNX2 RASA1 HDAC1 NFKB1 SKIL DDX20 WASL SOS1 SNIP1 EPHA2 SMAD3 PLCG1 ARNT ABCA1 SKP2 NRIP1 KLF6 RIF1 PDPK1 ATM SIN3A RBL2 SMAD4 FXR1 CUL1 ROCK1 LRIF1 PML PPARA BMPR2 CBL SP1 XPO1 MED1 SRSF1 MRE11 IGF1R TRAF1 WRN KMT2A FLNA SMAD5 |
| Head and Neck Squamous Cell Carcinoma (HNSC) | 170 | EP300 NINL CDK8 CDK1 UBE2W BTK JAK2 RAB5A TRAF6 LRP1 MDM2 EGFR CREB1 CD28 ABL1 NCOR1 KNG1 RUNX2 RAP2A RASA1 MBP TERF1 DDX20 ERBB4 NOS1 SMAD3 APBB1 LEF1 STAT3 ATN1 RBL2 FXR1 CASP3 LRIF1 CCR1 LYN SP1 MRE11 NOS3 WRN CRKL PAK2 GATA4 SMAD5 FGFR1 ZNF24 SMAD9 NCOA3 EPS15 CBX1 DAG1 GNA13 HDAC4 CCNT1 ACVR1 TCEA1 LCP2 CD22 XRCC5 NFKB1 LDB1 LNX1 KAT2B DVL2 COIL PPARG MCM3 PDPK1 PARP1 DAB2 NSF CSTF2 CBL ABI1 GNAQ ETS1 TRAF1 CD5 SRSF2 CRK RAD21 LAMA4 VHL TRAF5 NCOR2 BCL2 THRB ATR MED14 AHR SVIL ITK NCOA6 BRCA2 OCLN NCOA1 MCM2 RHOQ CDK2 RHOJ NCK1 SKIL PTN SNIP1 PLCG1 SKP2 DLG3 PLS1 RIF1 ITPR1 ATM CD9 SMAD4 CUL1 DHX9 GRB10 BMPR2 ERBB3 XPO1 MED1 TOP2A IGF1R HIF1A KMT2A LMO3 INSR JAK1 HIPK2 CBX5 PLD1 CDC6 TIAM1 FYN AGO2 KIT BARD1 PRKDC IRS2 DOCK1 SMAD2 SPOP TYK2 SMAD1 LRP2 APC GNA12 ESR2 WASL SOS1 ARNT ABCA1 ASF1B GFI1B MTOR RAF1 RBBP4 SIN3A ROCK1 PTPRS PPARA DVL3 SRSF1 MAP1B NCALD |
| Kidney Chromophobe (KICH) | 67 | CDK8 ZNF24 SMAD9 PLD1 UBE2W C1QBP AGO2 JAK2 RAB5A BARD1 EPS15 PRKDC GNA13 STAT1 SNW1 SMAD2 TRAF6 BRCA2 OCLN SPOP CREB1 CCNT1 RAB4A NCOR1 NCOA1 TCEA1 RASA1 CFLAR XRCC5 TERF1 RNF11 APC DDX20 KAT2B ERBB4 WASL SOS1 SKP2 COIL NRIP1 MTOR PLS1 RIF1 RBBP4 CDC42 ATM RBL2 VDAC1 PARP1 DHX9 ROCK1 NSF CSTF2 BMPR2 SP1 ABI1 XPA MED1 MRE11 HDAC2 HIF1A WRN CRK JAK1 RAD21 CBX5 SMAD5 |
| Kidney Clear Cell Carcinoma (KIRC) | 110 | EP300 NINL CDK8 BCL2 THRB IRS1 ATR MED14 UBE2W AHR SVIL JAK2 TERF2 NCOA6 TRAF6 BRCA2 OCLN MDM2 EGFR CREB1 RAB4A NCOR1 NCOA1 RAP2A NCK1 RASA1 MBP SKIL TERF1 DDX20 NOS1 SNIP1 SMAD3 SKP2 DLG3 KLF6 PLS1 RIF1 ITPR1 ATM RBL2 SMAD4 DHX9 BMPR2 SP1 ERBB3 XPO1 MED1 MRE11 IGF1R TEC HIF1A WRN KMT2A JAK1 HIPK2 CBX5 PAK2 SMAD5 ZNF24 SMAD9 PLD1 NCOA3 AGO2 BARD1 EPS15 PRKDC ESR1 GNA13 STAT1 DOCK1 SMAD2 HDAC4 CCNT1 ACVR1 LRP2 CFLAR NFKB1 LDB1 RNF11 APC ESR2 KAT2B WASL SOS1 PTEN ARNT ABCA1 COIL NRIP1 MTOR RBBP4 PDPK1 SIN3A GRB14 PARP1 ROCK1 DAB2 PPARA NSF CSTF2 CBL ABI1 GNAQ XPA HOOK2 SRSF1 CRK RAD21 VHL |
| Kidney renal papillary cell carcinoma (KIRP) | 134 | PNMA1 EP300 NINL TRAF5 CDK8 THRB IRS1 ATR MED14 UBE2W SVIL JAK2 TERF2 NCOA6 SNW1 TRAF6 BRCA2 OCLN MDM2 EGFR CREB1 RAB4A ABL1 NCOR1 NCOA1 KNG1 RAP2A NCK1 RASA1 MBP ATG12 SKIL TERF1 DDX20 ERBB4 RAP1A NOS1 SNIP1 SMAD3 RBM23 SKP2 KLF6 PLS1 RIF1 ITPR1 ATM RBL2 SMAD4 FXR1 CUL1 DHX9 LRIF1 BMPR2 SP1 XPO1 MED1 MRE11 TOP2A IGF1R HIF1A WRN KMT2A INSR JAK1 HIPK2 CBX5 CRKL PAK2 SMAD5 ZNF24 SMAD9 CDC6 PLD1 NCOA3 AGO2 BARD1 EPS15 CCND1 PRKDC ESR1 DAG1 GNA13 STAT1 DOCK1 SMAD2 HDAC4 SMAD1 CCNT1 ACVR1 LRP2 TCEA1 PIAS4 CFLAR XRCC5 NFKB1 LDB1 RNF11 APC HAP1 LNX1 KAT2B WASL SOS1 PTEN TAF7 ARNT ABCA1 MYH9 COIL NRIP1 MTOR RBBP4 PDPK1 MYH10 SIN3A VDAC1 TBP PARP1 ROCK1 PTPRS DAB2 PPARA NSF CSTF2 CBL ABI1 GNAQ SRSF1 HDAC2 ETS1 MAP1B CRK RAD21 VHL |
| Liver Hepatocarcinoma Cancer (LIHC) | 117 | EP300 CDK8 CD82 THRB IRS1 ATR FGF1 MED14 UBE2W AHR SVIL JAK2 TERF2 RAB5A NCOA6 TRAF6 BRCA2 LRP1 OCLN MDM2 GNAI1 EGFR CREB1 ABL1 NCOR1 NCOA1 KNG1 CDK2 RHOJ RAP2A NCK1 RASA1 MBP SKIL DDX20 RAP1A NOS1 SNIP1 SMAD3 PLCG1 RBM23 SKP2 KLF6 PLS1 RIF1 ITPR1 ATM RBL2 SMAD4 CUL1 DHX9 GRB10 LRIF1 BMPR2 SP1 XPO1 MED1 MRE11 TEC HIF1A WRN KMT2A JAK1 HIPK2 JUN CRKL PAK2 SMAD5 ARF6 ZNF24 SMAD9 NCOA3 KIT BARD1 EPS15 CCND1 ESR1 IRS2 DAG1 GNA13 DOCK1 HDAC4 SMAD1 CCNT1 ACVR1 CFLAR NFKB1 LDB1 RNF11 APC LNX1 KAT2B WASL SOS1 PTEN ARNT ABCA1 MYH9 NRIP1 MTOR RAF1 SMAD7 RBBP4 PDPK1 MYH10 SIN3A VAV2 ROCK1 PPARA DVL3 NSF CBL ABI1 GNAQ ETS1 CRK VHL |
| Brain Lower Grade Glioma (LGG) | 153 | MMP2 CD44 NTRK1 MMP7 EP300 TRAF5 NCOR2 CDK8 CDK1 IRS1 ATR MED14 UBE2W AHR BTK SVIL JAK2 RAB5A CASP1 ITK NCOA6 TRAF6 BRCA2 LRP1 OCLN MDM2 EGFR CREB1 CD28 ABL1 NCOR1 NCOA1 MCM2 RHOQ CDK2 RHOJ RUNX2 NCK1 RASA1 HDAC1 ATG12 SKIL TERF1 CASP4 DDX20 ZAP70 RAP1A SNIP1 SMAD3 PLCG1 SKP2 KLF6 LEF1 PLS1 RIF1 STAT3 ATM CENPA RBL2 CDC20 SMAD4 FXR1 CASP3 CUL1 DHX9 LRIF1 CCR1 LYN BMPR2 SP1 XPO1 MED1 MRE11 TOP2A IGF1R CAV1 HIF1A WRN KMT2A INSR JAK1 CBX5 CRKL PAK2 SMAD5 ARF6 ZNF24 SMAD9 CDC6 WNT3A NCOA3 AGO2 BARD1 CCND1 PRKDC ESR1 IRS2 DAG1 GNA13 STAT1 SMAD2 TYK2 SMAD1 CCNT1 ACVR1 TCEA1 LCP2 CFLAR XRCC5 NFKB1 APC ESR2 CDK3 KAT2B SOS1 PLCB2 TAF7 EPHA2 PLK1 DVL2 ARNT ABCA1 MYH9 ASF1B COIL NRIP1 FLNC MTOR MCM3 RAF1 PCNA RBBP4 PDPK1 MYH10 SIN3A VAV2 ROCK1 DAB2 DVL3 CSTF2 CBL RALA HOOK2 SRSF1 ETS1 TRAF1 SRSF2 CRK CCR5 RAD21 LAMA4 FLNA VHL |
| Lung Adenocarcinoma (LUAD) | 111 | CD44 EP300 TRPM6 CD82 BCL2 THRB ATR MED14 BTK JAK2 RAB5A CASP1 ITK NCOA6 TRAF6 BRCA2 LRP1 OCLN MDM2 GNAI1 EGFR CREB1 CD28 NCOR1 NCOA1 KNG1 RHOJ NCK1 RASA1 MBP SKIL DDX20 ERBB4 RAP1A NOS1 SNIP1 SMAD3 APBB1 RRAS2 PLS1 RIF1 STAT3 ITPR1 ATM RBL2 SMAD4 ACTN2 DHX9 CCR1 BMPR2 SP1 ERBB3 XPO1 MED1 MRE11 IGF1R CAV1 TEC WRN KMT2A JAK1 HIPK2 CBX5 CRKL PAK2 SMAD5 ZNF24 SMAD9 FGFR3 TIAM1 NCOA3 CAV3 BARD1 EPS15 PRKDC DOCK1 SMAD2 HDAC4 CCNT1 FEZ1 NFKB1 LDB1 RNF11 APC ESR2 LNX1 KAT2B VAMP2 SOS1 PTEN ARNT ABCA1 GFI1B MTOR RBBP4 PDPK1 MYH10 SIN3A ROCK1 DAB2 PPARA CBL ABI1 GNAQ XPA SRSF1 ETS1 CD5 CRK CCR5 VHL |
| Lung Squamous Adenocarcinoma (LUSC) | 108 | EP300 NINL TRPM6 CDK8 BCL2 ATR MED14 SVIL SET NCOA6 TRAF6 BRCA2 MDM2 EGFR CREB1 NCOR1 NCOA1 MCM2 RHOQ CDK2 SKIL TERF1 PTN NOS1 SMAD3 PLCG1 SKP2 XRCC6 LEF1 RIF1 ATN1 ATM CD9 RBL2 SMAD4 FXR1 CUL1 DHX9 SP1 XPO1 MED1 MRE11 TOP2A IGF1R WRN KMT2A WDR5 INSR HIPK2 CBX5 CRKL PAK2 GATA4 SMAD5 FGFR1 ZNF24 SMAD9 CDC6 PLD1 TIAM1 MYOD1 HCK NCOA3 AGO2 BARD1 CBX1 PRKDC IRS2 DAG1 GNA13 SMAD2 HDAC4 FXR2 SMAD1 CCNT1 FEZ1 PIAS4 LDB1 APC HAP1 LNX1 CDK3 SOS1 DVL2 ABCA1 COIL PPARG MCM3 CSK PDPK1 MYH10 SIN3A PARP1 ROCK1 PTPRS PPARA DVL3 NSF CSTF2 CBL XPA HOOK2 SRSF1 HDAC2 MAP1B CRK RAD21 VHL |
| Diffuse Large B Cell Lymphoma (DBLC) | 112 | EP300 CDK8 IRS1 ATR MED14 UBE2W AHR UBE2N SVIL JAK2 RAB5A ITK NCOA6 TRAF6 BRCA2 OCLN MDM2 EGFR CREB1 CD28 ABL1 NCOR1 NCOA1 RHOQ CDK2 RAP2A NCK1 RASA1 MBP SKIL TERF1 DDX20 RAP1A SNIP1 SMAD3 SKP2 KLF6 RIF1 STAT3 ATN1 ITPR1 ATM RBL2 SMAD4 DHX9 LRIF1 CCR1 LYN BMPR2 SP1 XPO1 MED1 MRE11 TOP2A IGF1R HIF1A WRN KMT2A JAK1 HIPK2 JUN CRKL PAK2 SMAD5 ZNF24 SMAD9 TIAM1 NCOA3 PCBP1 AGO2 KIT BARD1 PRKDC DAG1 GNA13 STAT1 DOCK1 HDAC4 CCNT1 ACVR1 LCP2 PIAS4 NFKB1 LDB1 RNF11 APC GNA12 LNX1 KAT2B SOS1 ARNT ABCA1 MYH9 COIL MTOR RAF1 RBBP4 PDPK1 SIN3A ROCK1 DAB2 PPARA DVL3 CBL ABI1 GNAQ SRSF1 ETS1 CRK CCR5 RAD21 VHL |
| Ovarian Serous Cystadenocarcinoma (OV) | 169 | MMP7 EP300 NINL CDK1 CD82 UBE2W UBE2N BTK JAK2 RAB5A CASP1 TRAF6 LRP1 MDM2 EGFR CREB1 CD28 NCOR1 KNG1 RUNX2 RAP2A RGS12 RASA1 MBP HDAC1 TERF1 CASP4 DDX20 ZAP70 NOS1 SMAD3 EHMT2 KLF6 RRAS2 LEF1 ATN1 PPIB RBL2 FXR1 LRIF1 LYN SP1 FOS MRE11 WRN CIB1 RND1 SMAD5 ZNF24 NCOA3 CAV3 EPS15 ESR1 GNA13 PLD2 HDAC4 CCNT1 CD22 XRCC5 UBE2U NFKB1 LDB1 RNF11 KAT2B COIL MCM3 CSK FURIN SMAD7 PDPK1 VAV2 MEN1 PML CSTF2 CBL HOOK2 TRAF1 CD5 CRK CCR5 RAD21 VHL CD44 TRIB3 TRAF5 NCOR2 BCL2 IRS1 ATR MED14 AHR SVIL TAF9 ITK MYC NCOA6 BRCA2 OCLN RAB4A NCOA1 ATG12 SKIL PEBP1 RAP1A SNIP1 RAC2 DLG3 RIF1 ITPR1 ATM SMAD4 EPOR DHX9 VDR BMPR2 ERBB3 XPO1 IGF1R TEC HIF1A KMT2A INSR JAK1 HIPK2 CBX5 JUN CSF1R UBC NFKB2 TIAM1 TRAF2 AGO2 BARD1 CCND1 PRKDC IRS2 TRIP6 DOCK1 TYK2 SMAD1 LRP2 CFLAR APC ESR2 CDK3 VAMP2 WASL SOS1 PTEN PLCB2 TAF7 ABCA1 NRIP1 GFI1B MTOR RAF1 RBBP4 MYH10 SIN3A VDAC1 ROCK1 PTPRS PPARA DVL3 RALA XPA SRSF1 JAK3 RAB8A |
| Pancreatic adenocarcinoma (PAAD) | 154 | MMP2 EP300 TRPM6 CDK8 GRB2 BCL2 THRB ATR MED14 UBE2W AHR BTK SVIL JAK2 TERF2 CASP1 ITK NCOA6 TRAF6 BRCA2 LRP1 OCLN MDM2 GNAI1 EGFR CREB1 CD28 ABL1 NCOR1 NCOA1 RHOQ RHOJ RUNX2 SDC3 RAP2A NCK1 RASA1 MBP ATG12 SKIL TERF1 DDX20 ERBB4 ZAP70 RAP1A NOS1 SNIP1 PLCG1 RBM23 SKP2 LEF1 RIF1 STAT3 ITPR1 ATM RBL2 SMAD4 CASP3 CUL1 DHX9 GRB10 LRIF1 CCR1 LYN BMPR2 SP1 XPO1 MED1 MRE11 IGF1R HIF1A WRN KMT2A LMO3 JAK1 HIPK2 CBX5 CSF1R CRKL PAK2 SMAD5 FGFR1 ZNF24 SMAD9 PLD1 TIAM1 HCK NCOA3 FYN KIT BARD1 EPS15 CBX1 PRKDC ESR1 GNA13 STAT1 DOCK1 SMAD2 HDAC4 SPOP SMAD1 CCNT1 ACVR1 TCEA1 LCP2 CCL5 CFLAR CD22 RAB1A UBE2U NFKB1 LDB1 RNF11 APC GNA12 ESR2 KAT2B WASL SOS1 PTEN PLCB2 ARNT ABCA1 MYH9 COIL NRIP1 GFI1B MTOR SMAD7 RBBP4 PDPK1 MYH10 SIN3A TBP PARP1 ROCK1 DAB2 PPARA NSF CBL ABI1 GNAQ SRSF1 ETS1 TRAF1 CD5 MAP1B NCALD CRK CCR5 RAD21 LAMA4 VHL |
| Pheochromocytoma and Paraganglioma (PCPG) | 99 | PNMA1 CD44 EP300 NINL NCOR2 CDK8 ATR MED14 UBE2W SVIL JAK2 TAF9 TERF2 NCOA6 SNW1 TRAF6 BRCA2 OCLN MDM2 GNAI1 CREB1 NCOR1 NCOA1 MCM2 RASA1 MBP ATG12 SKIL TERF1 DDX20 CCNA1 NOS1 SMAD3 PLCG1 SKP2 PLS1 RIF1 ITPR1 ATM RBL2 SMAD4 EPOR CUL1 DHX9 LRIF1 LYN BMPR2 XPO1 MED1 MRE11 TOP2A IGF1R WRN KMT2A HIPK2 CBX5 SMAD5 ZNF24 SMAD9 AGO2 BARD1 CBX1 PRKDC IRS2 DAG1 GNA13 SMAD2 HDAC4 CCNT1 FEZ1 PIAS4 XRCC5 LDB1 APC GNA12 ESR2 KAT2B WASL SOS1 PTEN TAF7 ARNT COIL PDPK1 MYH10 SIN3A PARP1 ROCK1 PTPRS PPARA NSF CSTF2 CBL ABI1 GNAQ XPA MAP1B RAD21 VHL |
| Prostate adenocarcinoma (PRAD) | 145 | CD44 EP300 NCOR2 CDK8 CDK1 THRB IRS1 ATR MED14 UBE2W AHR JAK2 TERF2 RAB5A ITK MYC NCOA6 TRAF6 BRCA2 LRP1 OCLN MDM2 GNAI1 EGFR NDRG1 CREB1 CD28 ABL1 NCOR1 NCOA1 MCM2 CDK2 RUNX2 RAP2A NCK1 RASA1 MBP ATG12 SKIL TERF1 DDX20 ERBB4 NOS1 SNIP1 PLCG1 SKP2 KLF6 PLS1 RIF1 STAT3 ITPR1 ATM RBL2 SMAD4 FXR1 CASP3 DHX9 VDR LRIF1 CCR1 LYN BMPR2 SP1 ERBB3 XPO1 MED1 MRE11 TOP2A IGF1R TEC HIF1A WRN KMT2A INSR JAK1 HIPK2 CBX5 CRKL PAK2 SMAD5 ARF6 ZNF24 SMAD9 CDC6 NCOA3 AGO2 KIT BARD1 EPS15 PRKDC DAG1 GNA13 STAT1 DOCK1 SMAD2 HDAC4 SMAD1 CCNT1 ACVR1 LRP2 PIAS4 CFLAR XRCC5 RAB1A NFKB1 LDB1 RNF11 APC GNA12 LNX1 KAT2B WASL SOS1 PTEN ARNT ABCA1 MYH9 COIL NRIP1 MTOR RBBP4 PDPK1 MYH10 SIN3A ANXA5 PARP1 ROCK1 PTPRS DAB2 TLE1 PPARA NSF CSTF2 CBL ABI1 GNAQ SRSF1 HDAC2 ETS1 MAP1B NCALD CRK CCR5 RAD21 VHL |
| Sarcoma (SARC) | 110 | EP300 NCOR2 CDK8 BCL2 THRB IRS1 ATR MED14 UBE2W JAK2 RAB5A NCOA6 TRAF6 BRCA2 OCLN EGFR CREB1 ABL1 NCOR1 NCOA1 CDK2 RAP2A NCK1 RASA1 ATG12 SKIL TERF1 DDX20 ERBB4 NOS1 SNIP1 SMAD3 PLCG1 SKP2 PLS1 RIF1 STAT3 ITPR1 ATM RBL2 SMAD4 CUL1 DHX9 LRIF1 BMPR2 SP1 XPO1 MED1 MRE11 TOP2A IGF1R WRN KMT2A JAK1 HIPK2 CBX5 CRKL PAK2 SMAD5 ZNF24 SMAD9 CDC6 NCOA3 AGO2 BARD1 EPS15 CBX1 PRKDC DAG1 GNA13 DOCK1 SMAD2 HDAC4 CCNT1 ACVR1 TCEA1 PIAS4 LDB1 RNF11 APC GNA12 KAT2B WASL SOS1 TAF7 DVL2 ARNT ABCA1 COIL NRIP1 FLNC MTOR RAF1 RBBP4 PDPK1 MYH10 SIN3A ROCK1 PPARA DVL3 NSF CSTF2 CBL ABI1 GNAQ SRSF1 MAP1B CRK RAD21 VHL |
| Skin Cutaneous Melanoma (SKCM) | 123 | CD44 EP300 TRAF5 CDK8 CDK1 BCL2 THRB IRS1 ATR MED14 UBE2W AHR SVIL JAK2 TAF9 RAB5A MYC SRSF3 SNW1 TRAF6 BRCA2 OCLN MDM2 GNAI1 CREB1 RAB4A NCOR1 NCOA1 RHOQ RUNX2 RAP2A NCK1 RASA1 ATG12 SKIL TERF1 DDX20 HMGB1 RAP1A SNIP1 SKP1 SKP2 KLF6 RRAS2 PLS1 RIF1 ITPR1 CDC42 ATM RBL2 SMAD4 FXR1 CASP3 CUL1 DHX9 LRIF1 BMPR2 SP1 ERBB3 XPO1 MED1 MRE11 TOP2A IGF1R CAV1 TEC HIF1A WRN KMT2A CBX5 PAK2 SMAD5 ZNF24 NCOA3 BARD1 EPS15 PRKDC ESR1 IRS2 GNA13 STAT1 DOCK1 SMAD2 HDAC4 SPOP SMAD1 CCNT1 ACVR1 TCEA1 NFKB1 RNF11 APC LNX1 CDK3 KAT2B WASL SOS1 PTEN TAF7 ARNT COIL NRIP1 MTOR SMAD7 RBBP4 PDPK1 SIN3A ROCK1 PPARA NSF EIF1B CBL ABI1 RALA GNAQ XPA SRSF1 HDAC2 ETS1 CRK RAD21 LAMA4 VHL |
| Stomach Adenocarcinoma (STAD) | 113 | CD44 EP300 TRAF5 NCOR2 CDK8 BCL2 ATR MED14 UBE2W AHR JAK2 CASP1 ITK NCOA6 TRAF6 BRCA2 OCLN MDM2 EGFR CREB1 CD28 NCOR1 NCOA1 RUNX2 RAP2A RASA1 MBP SKIL DDX20 ZAP70 SNIP1 SMAD3 PLCG1 SKP2 DLG3 PLS1 RIF1 ITPR1 ATM RBL2 SMAD4 CASP3 CUL1 DHX9 LYN BMPR2 SP1 ERBB3 XPO1 MED1 MRE11 TOP2A IGF1R TEC HIF1A WRN KMT2A JAK1 HIPK2 CBX5 CRKL PAK2 SMAD5 ZNF24 PLD1 TIAM1 NCOA3 CAV3 AGO2 BARD1 EPS15 PRKDC DAG1 GNA13 DOCK1 SMAD2 HDAC4 TYK2 SMAD1 CCNT1 CFLAR NFKB1 APC ESR2 CDK3 KAT2B WASL SOS1 PTEN MAST2 PLCB2 ARNT ABCA1 NRIP1 MTOR RAF1 RBBP4 PDPK1 SIN3A ROCK1 PPARA NSF CBL ABI1 GNAQ HOOK2 SRSF1 ETS1 CD5 CRK JAK3 RAD21 VHL |
| Testicular Germ Cell Tumors (TGCT) | 109 | PNMA1 MMP2 EP300 TRPM6 CDK8 ATR UBE2N DCTN1 JAK2 NCF1 ITK NCOA6 SNW1 TRAF6 BRCA2 OCLN NR4A1 CREB1 CD28 ABL1 NCOR1 NCOA1 CDK2 NCK1 RASA1 MBP SKIL DDX20 ZAP70 SNIP1 RAC2 SMAD3 PLCG1 RBM23 XRCC6 DLG3 RIF1 ITPR1 ATM CENPA RBL2 DHX9 GRB10 LRIF1 SP1 XPO1 MRE11 IGF1R TEC HIF1A WRN KMT2A WDR5 JAK1 GATA4 SMAD5 ZNF24 SMAD9 FGFR3 TIAM1 NCOA3 KIT BARD1 EPS15 PRKDC ESR1 GNA13 STAT1 SMAD2 TYK2 CCNT1 ACVR1 LCP2 PIAS4 CFLAR XRCC5 UBE2U LDB1 RNF11 APC GNA12 LIMK1 ESR2 KAT2B WASL SOS1 PTEN MAST2 TAF7 ARNT ABCA1 PPARG MTOR CSK RAF1 SIN3A VAV2 ROCK1 PTPRS DVL3 NSF CBL ABI1 GNAQ CD5 CRK CCR5 RAD21 VHL |
| Thymona (Thym) | 168 | PNMA1 MMP2 EP300 CDK8 CD82 UBE2W JAK2 RAB5A CASP1 TRAF6 LRP1 MDM2 GNAI1 EGFR NDRG1 ERBB2 CREB1 ABL1 NCOR1 RUNX2 SDC3 RAP2A RASA1 MBP TERF1 DDX20 ERBB4 NOS1 SMAD3 GRB7 KLF6 STAT3 ATN1 RBL2 FXR1 LRIF1 CCR1 LYN SP1 FOS MRE11 NOS3 WRN CRKL GATA4 SMAD5 ZNF24 SMAD9 NCOA3 CAV3 EPS15 DAG1 GNA13 STAT1 PLD2 CCNT1 ACVR1 SRC RAB1A NFKB1 LDB1 RNF11 HAP1 LNX1 KAT2B EPHA2 COIL FLNC FURIN SMAD7 PDPK1 VAV2 GRB14 ANXA5 RARA TLE1 NSF CBL ABI1 GNAQ VAMP3 CRK CCR5 RAD21 LAMA4 VHL NCOR2 BCL2 THRB IRS1 ATR MED14 AHR SVIL NCOA6 BRCA2 OCLN RAB4A NCOA1 RHOQ RHOJ NCK1 PAK4 ATG12 SKIL CCNA1 RAP1A RXRA SNIP1 SKP2 DLG3 PLS1 RIF1 ITPR1 BCAR1 ATM CD9 SMAD4 EPOR CUL1 DHX9 VDR GRB10 BMPR2 ERBB3 XPO1 MED1 IGF1R CAV1 HIF1A KMT2A INSR JAK1 HIPK2 CBX5 JUN CSF1R FGFR3 PLD1 TIAM1 KIT BARD1 CCND1 PRKDC IRS2 DOCK1 SMAD2 SMAD1 LRP2 CFLAR APC GNA12 LIMK1 WASL SOS1 ARNT ABCA1 MYH9 NRIP1 MTOR RBBP4 MYH10 SIN3A ROCK1 PPARA DVL3 MAP1B FLNA |
| Thyroid carcinoma (THCA) | 134 | IRAK1 EP300 TRIB3 NINL TRPM6 CDK8 BCL2 THRB IRS1 ATR MED14 UBE2W SVIL JAK2 TERF2 RAB5A SNTA1 NCOA6 TRAF6 BRCA2 OCLN MDM2 GNAI1 EGFR CREB1 RAB4A ABL1 NCOR1 NCOA1 RHOQ RHOJ RAP2A NCK1 RASA1 MBP ATG12 SKIL TERF1 DDX20 ERBB4 RXRA NOS1 SNIP1 PLCG1 RBM23 SKP2 NUPR1 RRAS2 PLS1 RIF1 STAT3 ITPR1 ATM RBL2 SMAD4 FXR1 CUL1 DHX9 GRB10 LRIF1 BMPR2 SP1 XPO1 MED1 MRE11 IGF1R WRN KMT2A JAK1 HIPK2 CBX5 CRKL PAK2 SMAD5 FGFR1 ZNF24 SMAD9 PLD1 NCOA3 AGO2 KIT BARD1 EPS15 CBX1 PRKDC DAG1 GNA13 DOCK1 SMAD2 HDAC4 SPOP SMAD1 CCNT1 LRP2 TCEA1 PIAS4 RAB1A NFKB1 LDB1 BEX3 RNF11 APC HAP1 ESR2 LNX1 KAT2B WASL SOS1 PTEN ARNT ABCA1 COIL MTOR RAF1 RBBP4 PDPK1 SIN3A TBP GRB14 PARP1 ROCK1 TLE1 PPARA DVL3 NSF CSTF2 CBL GNAQ HOOK2 SRSF1 MAP1B CRK RAD21 VHL |
| Uterine Carcinosarcoma (UCS) | 9 | ATR JAK2 BARD1 RBL2 CCNT1 NCOR1 RASA1 APC VHL |
| Uterine Corpus Endometrial Carcinoma (UCEC) | 151 | CD44 EP300 TRPM6 TRAF5 CDK8 CDK1 BCL2 ATR MED14 UBE2W AHR UBE2N SVIL SET JAK2 TAF9 TERF2 RAB5A ITK SRSF3 NCOA6 SNW1 TRAF6 BRCA2 OCLN MDM2 GNAI1 EGFR CREB1 CD28 RAB4A ABL1 NCOR1 NCOA1 RHOQ KNG1 CDK2 RUNX2 MED31 RAP2A NCK1 RASA1 MBP ATG12 SKIL TERF1 DDX20 HMGB1 RAP1A SNIP1 SKP2 KLF6 RRAS2 LEF1 PLS1 RIF1 STAT3 ITPR1 CDC42 ATM RBL2 SMAD4 CASP3 DHX9 PTMA GRB10 LRIF1 LYN BMPR2 SP1 XPO1 MED1 MRE11 TOP2A CAV1 TEC HIF1A WRN KMT2A JAK1 HIPK2 CBX5 CRKL PAK2 SMAD5 ARF6 ZNF24 SMAD9 CDC6 PLD1 NCOA3 KIT BARD1 EPS15 PRKDC ESR1 GNA13 DOCK1 SMAD2 SPOP SMAD1 CCNT1 ACVR1 TCEA1 LCP2 XRCC5 RAB1A NFKB1 RNF11 APC ESR2 LNX1 KAT2B WASL SOS1 PTEN TAF7 ARNT ABCA1 COIL NRIP1 PPARG RHOA MTOR RAF1 RBBP4 PDPK1 SIN3A VDAC1 GRB14 ANXA5 ROCK1 DAB2 PPARA NSF EIF1B CBL ABI1 GNAQ SRSF1 HDAC2 ETS1 SNX3 CD5 SRSF2 MAP1B NCALD CRK RAD21 LAMA4 VHL |
| Uveal Melanoma (UVM) | 142 | PNMA1 SUMO1 EP300 NINL TRPM6 TRAF5 CDK8 CDK1 BCL2 THRB ATR FGF1 MED14 UBE2W AHR UBE2N SET JAK2 TAF9 RAB5A CASP1 MYC SRSF3 SNW1 TRAF6 BRCA2 OCLN MDM2 GNAI1 CREB1 NCOR1 NCOA1 RHOQ RUNX2 MED31 RAP2A NCK1 RASA1 MBP ATG12 SKIL TERF1 CASP4 DDX20 HMGB1 ERBB4 RAP1A SKP1 SKP2 DLG3 KLF6 RRAS2 PLS1 RIF1 ITPR1 CDC42 ATM CD9 RBL2 SMAD4 ACTN2 FXR1 CASP3 CUL1 DHX9 RGS2 LRIF1 LYN BMPR2 SUMO2 SP1 XPO1 MED1 MRE11 TOP2A TEC HIF1A WRN KMT2A PAK2 SMAD5 ARF6 ZNF24 SMAD9 CDC6 PLD1 NCOA3 BARD1 EPS15 CBX1 PRKDC ESR1 GNA13 STAT1 DOCK1 SMAD2 HDAC4 SPOP SMAD1 CCNT1 TCEA1 CFLAR XRCC5 RAB1A NFKB1 RNF11 APC ESR2 LNX1 CDK3 KAT2B WASL SOS1 PTEN TAF7 ABCA1 COIL NRIP1 SMAD7 PCNA RBBP4 PDPK1 SIN3A TBP ROCK1 PPARA NSF CSTF2 CBL ABI1 RALA GNAQ XPA HOOK2 SRSF1 HDAC2 ETS1 TRAF1 SRSF2 CRK RAD21 VHL |
